# Selective Isolation of Multidrug-Resistant *Pedobacter* spp., Producers of Novel Antibacterial Peptides

**DOI:** 10.3389/fmicb.2021.642829

**Published:** 2021-02-25

**Authors:** Joakim Bjerketorp, Jolanta J. Levenfors, Christina Nord, Bengt Guss, Bo Öberg, Anders Broberg

**Affiliations:** ^1^Department of Molecular Sciences, Uppsala BioCentrum, Swedish University of Agricultural Sciences, Uppsala, Sweden; ^2^Ultupharma AB, Uppsala, Sweden; ^3^Department of Biomedical Sciences and Veterinary Public Health, Swedish University of Agricultural Sciences, Uppsala, Sweden; ^4^Department of Medicinal Chemistry, Uppsala University, Uppsala, Sweden

**Keywords:** *Pedobacter*, self-resistance, selective isolation, multidrug-resistant bacteria, environmental bacteria, antibacterial cyclic peptides, Gram-negative antibiotics

## Abstract

Twenty-eight multidrug-resistant bacterial strains closely related or identical to *Pedobacter cryoconitis*, *Pedobacter lusitanus* and *Pedobacter steynii* were isolated from soil samples by selection for multidrug-resistance. Approximately 3–30% of the selected isolates were identified as *Pedobacter*, whereas isolation without antibiotics did not yield any isolates of this genus. Next generation sequencing data showed *Pedobacter* to be on 69th place among the bacterial genera (0.32% of bacterial sequences). The *Pedobacter* isolates produced a wide array of novel compounds when screened by UHPLC-MS/MSMS, and hierarchical cluster analysis resulted in several distinct clusters of compounds produced by specific isolates of *Pedobacter*, and most of these compounds were found to be peptides. The *Pedobacter* strain UP508 produced isopedopeptins, whereas another set of strains produced pedopeptins, which both are known cyclic lipodepsipeptides produced by *Pedobacter* sp. Other *Pedobacter* strains produced analogous peptides with a sequence variation. Further strains of *Pedobacter* produced additional novel antibacterial cyclic lipopeptides (ca 800 or 1400 Da in size) and/or linear lipopeptides (ca 700–960 Da in size). A 16S rRNA phylogenetic tree for the *Pedobacter* isolates revealed several distinct clades and subclades of isolates. One of the subclades comprised isolates producing isopedopeptin analogs, but the isopedopeptin producing isolate UP508 was clearly placed on a separate branch. We suggest that the non-ribosomal peptide synthases producing pedopeptins, isopedopeptins, and the analogous peptides, may derive from a common ancestral non-ribosomal peptide synthase gene cluster, which may have been subjected to a mutation leading to changed specificity in one of the modules and then to a modular rearrangement leading to the changed sequence found in the isopedopeptins produced by isolate UP508.

## Introduction

The discovery and use of antibiotics in human medicine have saved millions of lives, helped to increase our life expectancy and made possible many of the medical practices that are now standard in modern medicine. Most of all clinically relevant antibiotics originate from natural products made by microorganisms, and mainly soil bacteria (Demain, 2014). These natural products stem from the ancient struggle for existence during which an intricate web of antibiotic production and factors for defense or self-protection have evolved. Accordingly, it is quite common for soil bacteria to both carry genes coding for antibiotics as well as the corresponding antibiotic resistance genes (Omura et al., 2001; Bentley et al., 2002; D’Costa et al., 2006; Walsh and Duffy, 2013).

The use and overuse of antibiotics exert a strong selection pressure on affected microorganisms and as a result, many clinically essential antibacterial drugs against important pathogens are becoming less useful. The current situation with increasing antimicrobial resistance against antibiotics and an accelerated spread of antibiotic resistance genes is indeed of critical concern. To make bad even worse, the antibacterial pipeline has been insufficient for many years to keep resistant pathogenic bacteria and especially the Gram-negatives at bay (Theuretzbacher et al., 2019). Therefore, in order to tackle the most critical clinical needs more novel antibiotic leads must urgently be fed into the pipeline (World Health Organization [WHO], 2019a, b).

However, there are numerous obstacles to overcome when searching for novel antibiotics among natural products. The major part of the microorganisms known to be present in soils and other natural environments, as detected by metagenome studies, will not grow easily in the laboratory, which makes these potentially useful microbes difficult to access. On the other hand, many cultivable microorganisms often produce already known compounds that in turn prompts the need for efficient de-replication. Consequently, it can be difficult to isolate producers of novel and promising antibiotics. In order to challenge these obstacles, some researchers have pursued *in situ* isolation methods to expand the cultivable part of the soil microbiota and successfully found novel and promising antibiotic lead molecules (Ling et al., 2015). Others have focused on the inherent need for antibiotics producing bacteria to be self-resistant and thus used selected single antibiotics during the isolation of bacteria, with the aim to increase the proportion of bacterial isolates that can produce compounds similar to the supplemented antibiotic (Thaker et al., 2013, 2014).

For clinically relevant pathogenic bacteria, the definition of antibiotic resistance is based on their clinical breakpoint values, but for environmental bacteria the definition is less straightforward. A couple of studies on soil microorganisms have simply used the definition of antibiotic resistant bacteria as those that can grow in the presence of 20 mg/L of the tested antibiotic (D’Costa et al., 2006; Walsh and Duffy, 2013). Examples of environmental antibiotic resistant bacteria are members of the Gram-negative genus *Pedobacter*, that have been found to be resistant against numerous antibiotics from several classes (Viana et al., 2018). These included the facultative psychrophilic bacterium *P. cryoconitis*, which was first described after being isolated from the dark windblown debris called cryoconite on a glacier in Austria (Margesin et al., 2003), *Pedobacter lusitanus*, which is a close relative to *P. cryoconitis*, first isolated from the sludge of a deactivated uranium mine in Portugal (Covas et al., 2017) and *Pedobacter steynii* that was first obtained from the creek Westerhöfer Bach in Germany (Muurholm et al., 2007). Further, in a recent study the antibiotic resistance of *P. cryoconitis*, *P. lusitanus* and a few more closely related *Pedobacter* species, were investigated in more detail (Viana et al., 2018). Several of the *Pedobacter* species in this study were found resistant to antibiotics belonging to three or more different classes of antibiotics, which categorized them as multidrug-resistant (MDR) environmental superbugs (Magiorakos et al., 2012). The antibiotic resistance of the *Pedobacter* members has been shown to depend on chromosomal genes and not genes on mobile genetic elements and approximately 6–8% of their protein coding genes have been estimated to be involved in providing antibiotic resistance (Viana et al., 2018). The genus *Pedobacter* has been rapidly expanding since the description of the first four species some 20 years ago (Steyn et al., 1998) and currently includes 87 species.^1^

We have recently adapted and extended the antibiotic resistance approach by Thaker et al. (2013, 2014) with the goal to find bacterial isolates talented in production of antibiotic compounds. We have reasoned that MDR environmental bacteria may have the corresponding capacity to produce more than one type of antibacterial compound. Thus, when isolating soil bacteria for subsequent screening for new antibiotic compounds, we have combined several antibiotics in order to specifically select for MDR bacteria. The use of this approach resulted in the isolation of *P. cryoconitis* UP508, which was found to produce isopedopeptins, which are cyclic lipodepsipeptides with potent activity against WHO top-priority bacterial pathogens (Nord et al., 2020). In the present paper, we describe the application of this methodology to isolate several MDR strains of *Pedobacter*, along with UHPLC-MS based hierarchical cluster analysis (HCA) of the metabolites produced by the isolates. Based on UHPLC-MSMS, we also characterize a number of peptides, of which only a few are previously known whereas the large majority are new chemical entities. Additionally, most of these novel peptides were indicated to have activity against important human Gram-negative and/or Gram-positive bacterial pathogens.

## Materials and Methods

### Soil Sample Preparation

Twelve soil samples (≥10 g) were collected from several different locations in Sweden (Table 1). Seven soil samples (1–7) were from Uppsala County along The Linnaeus Trails (Herbationes Upsalienses), one sample (8) was from Gävleborg County north of Uppsala, three samples (9–11) were from Norrbotten County, in the mountains above the Arctic Circle, and the last soil sample (12) came from a sandy beach in Gotland County, an island in the Baltic Sea. Samples were taken by pressing or digging a sterile 50 mL centrifuge tube into the soil surface. The samples were kept cold until the time of dilution and plating. Aliquots of the individual soil samples were deep frozen and selected samples were later sent in a frozen state to BaseClear BV (Leiden, Netherlands) for DNA extraction, NGS (next generation sequencing) and analysis of microbial community composition (Supplementary Material).

**TABLE 1:** Isolated *Pedobacter* strains, antibiotics used for their selection, closest established identity according to 16S rRNA gene sequences (GenBank accession numbers MW332355 to MW332382), and sample collection coordinates.

#^*a*^	Soil sample number; (geographical coordinates)	Isolate name	Antibiotics used for selection	Closest hit (BLAST^®^), ≥ 99% identity
**1**	1; (59°49′07.2″ N 17°39′55.6″ E)	UP508^*b*^	NAL, AMP, KAN	*P. cryoconitis* A37
		UP509	NAL, AMP, KAN	*P. cryoconitis* A37
	2; (59°48′58.8″ N 17°39′51.1″ E)	UP579	NAL, AMP, KAN	*P. steynii* WB2.3-45
	3; (59°48′58.9″ N 17°38′44.7″ E)	UP621	LIN, ERY, KAN	*P. cryoconitis* A37
		UP640	NAL, AMP, KAN	*P. cryoconitis* A37
	4; (59°49′4.3″ N 17°46′36.9″ E)	UP696	NAL, AMP, KAN	*P. lusitanus* NL19
	9; (68°3′15.0″ N 19°26′48.6″ E)	UP742	NAL, AMP, KAN	*P. cryoconitis* A37
		UP751	NAL, AMP, KAN	*P. cryoconitis* A37
**2**	6; (59°48′7.5″ N 17°41′17.7″ E)	UP1634	NAL, ERY	*P. cryoconitis* A37
		UP1637	NAL, VAN	*P. lusitanus* NL19
		UP1642	NAL, ERY, KAN, STR	*P. cryoconitis* A37
	7; (59°49′8.9″ N 17°39′19.3″ E)	UP1729	NAL, ERY, KAN, STR	*P. cryoconitis* A37
	8; (60°40′14.1″ N 16°48′58.2″ E)	UP1478	CIP, AMP, KAN, POL	*P. cryoconitis* A37
		UP1479	VAN, KAN	*P. cryoconitis* A37
	11; (68°28′55.0″ N 18°49′21.6″ E)	UP1184	NAL, ERY, GEN	*P. cryoconitis* A37
		UP1189	CIP, LIN, GEN	*P. cryoconitis* A37
		UP1400	NAL, VAN	*P. cryoconitis* A37
**3**	5; (59°49′8.1″ N 17°40′35.7″ E)	UP1440	NAL, AMP, KAN	*P. steynii* WB2.3-45
		UP1426	NAL, CIP, LIN, ERY, AMP, KAN, GEN	*P. steynii* WB2.3-45
	10; (68°28′24.7″ N 18°49′5.0″ E)	UP1427	NAL, AMP, KAN	*P. cryoconitis* A37
		UP1428	NAL, AMP, GEN	*P. cryoconitis* A37
		UP1429	NAL, AMP, GEN	*P. cryoconitis* A37
		UP1430	CIP, AMP, KAN	*P. cryoconitis* A37
		UP1431	CIP, AMP, GEN	*P. cryoconitis* A37
	12; (57°19′47.9″ N 18°42′43.0″ E)	UP1435	NAL, AMP, GEN	*P. cryoconitis* A37
		UP1436	NAL, AMP, KAN	*P. cryoconitis* A37
		UP1437	CIP, AMP, GEN	*P. lusitanus* NL19
		UP1439	NAL, LIN, GEN	*P. cryoconitis* A37

*The concentrations of antibiotics used for isolation were: 20 mg/L for NAL, AMP, KAN, STR, and ERY; 10 mg/L for LIN; 5 mg/L for GEN; 2 mg/L for VAN and POL; 1 mg/L for CIP. The standard settings found at (https://blast.ncbi.nlm.nih.gov/Blast.cgi) were used for the sequence alignment: Nucleotide BLAST against 16S RNA sequences (Bacteria and Archaea) optimized for highly similar sequences (Megablast). ^a^Isolation round 1–3. ^b^Deposited as LMG P-30868 at the Belgian Coordinated Collections of Micro-organisms.*

### Isolation of MDR *Pedobacter* spp.

As growth medium base for isolation of bacteria, a modified CN medium was used (Gavrish et al., 2008). The modified medium base was denoted as NBCA (Nutrient Broth/Casamino Acid agar medium) and contained: 1 g Difco Nutrient Broth (BD Difco Ltd., Detroit, MI, Untied States), 1 g Casamino Acids (BD Difco Ltd.), 15 g Bacto agar (Saveen & Werner, Limhamn, Sweden) in 1 L of deionized water. Fungicides and various antibiotics were added to NBCA after autoclaving and cooling the medium to 50°C. To reduce the growth of molds, two fungicides, cycloheximide, and nystatin, were added to the final concentration of (100 mg/L) and (10 mg/L), respectively. The NBCA was also supplemented with varying combinations of antibiotics to provide the selection conditions for the isolation of antibiotic resistant bacteria. Selected antibiotics covered different antibiotic classes and mechanisms of action and when supplemented to the NBCA the following final concentrations were used: ampicillin (AMP, 20 mg/L), kanamycin (KAN, 20 mg/L), nalidixic acid (NAL, 20 mg/L), streptomycin (STR, 20 mg/L) and erythromycin (ERY, 20 mg/L), lincomycin (LIN 10 mg/L) was 10 mg/L, gentamicin (GEN, 5 mg/L), vancomycin (VAN, 2 mg/L), polymyxin B (POL, 2 mg/L), and ciprofloxacin (CIP, 1 mg/L).

Approximately 4 g of each individual soil sample was thoroughly mixed by vortexing with 40 mL of sterile phosphate-buffered saline (PBS). The mixture was allowed to settle for at least 10 min and then 1 mL of the supernatant was mixed with 9 mL of sterile PBS in a 15 mL sterile centrifuge tube. From this dilution, 0.1 mL was spread onto the NBCA isolation plates without (control) or with different combinations of antibiotics. The plates were then incubated at 20°C for approximately one week in darkness to avoid degradation of light sensitive antibiotics.

Morphologically different bacterial colonies were afterward re-streaked on Vegetable Peptone Broth Agar plates (VPA), containing 10 g Vegetable Peptone Broth (VPB; Oxoid Ltd., Basingstoke, Hampshire, United Kingdom), and 15 g Bacto Agar (Saveen & Werner) in 1 L deionized water.

### Antibiotic Resistance Pattern of *P. cryoconitis* UP508

The antibiotic resistance pattern of isolate UP508 was established using NBCA agar base (as above) supplemented with the following individual antibiotics at 20 mg/L: AMP, CIP, ERY, GEN, KAN, LIN, NAL, POL, STR, and VAN. The isolate was inoculated on the plates, which were incubated for 2–3 days in darkness at room temperature and inspected for bacterial growth.

### Identification and Phylogeny

Twenty-eight bacterial strains isolated from soil samples were selected for identification by means of sequencing of the 16S rRNA genes. Sequencing was also performed for the five type isolates of different *Pedobacter* species purchased from the Belgian Co-ordinated Collections of Micro-organisms [BCCM/LMG, *Pedobacter cryoconitis* A37 (LMG 21415^T^) and *P. lusitanus* NL19 (LMG 29220^T^)], the German Collection of Microorganisms and Cell Cultures [DSMZ, *Pedobacter hartonius* WB 3.3-3 (DSM 19033^T^) and *Pedobacter westerhofensis* WB 3.3-22 (DSM 19036^T^)] as well as from the Japan Collection of Microorganisms [JCM, *Pedobacter himalayensis* HHS22 (JCM 12171^T^)]. Briefly, colony PCR amplification of the 16S rRNA gene was performed with the universal primers 27F (AGAGTTTGATCMTGGCTCAG) and 1492R (TACGGYTACCTTGTTACGACTT) followed by purification of amplified PCR-products (QIAquick PCR purifications kit, QIAGEN). Sequencing was then performed by Macrogen Europe (EZ-sequencing, Amsterdam, Netherlands). The resulting DNA sequencing chromatograms were assembled using the Geneious R8 software and manually inspected (Biomatters Ltd., Auckland, New Zealand). The resulting 16S rRNA sequences were deposited at GenBank (accession numbers MW332350 to MW332382). The BLAST database for 16S rRNA sequences (Altschul et al., 1997) of the National Center of Biotechnology Information (NCBI^2^) was then used to retrieve the closest possible identity of the isolates. For aligning the sequences, the online MAFFT version 7 multiple alignment program for amino acid or nucleotide sequences^3^ was used and Neighbor Joining (NJ) phylogenetic trees were obtained after estimating the robustness of the internal tree branches by bootstrap analysis (Jukes–Cantor/bootstrap 1000) using 1000 replications (Katoh et al., 2019).

### Culturing, Collection, and Extraction of Metabolites

Bacterial strains isolated from soil samples and the reference *Pedobacter* species, were grown in liquid cultures (150 mL in 500-mL Erlenmeyer flasks) using half strength VPB [15 g VPB (Oxoid Ltd.) in 1 L deionized water]. Cultures were either started by transferring a loop (10 μL) of 24–48 h-old bacterial colonies grown on VPA plates or by transferring 100 μL of deep-frozen isolate stock per 150 mL VPB. The cultures were incubated on a rotary shaker (130 rpm) for 96–120 h at 20°C in darkness. To collect extracellular metabolites, one sterile nylon mesh bag containing a polymeric resin, Sepabeads^®^ SP850 (Sigma-Aldrich), was submerged in each actively growing culture, approximately 16–24 h after inoculation (approximately 8 g of resin per bag). At harvest, each adsorbent resin bag was washed with deionized water to remove bacterial cells and culture resins. Each bag was subsequently extracted with 2 × 20 mL MeOH and 2 × 20 mL MeCN, and the extracts were pooled and analyzed by UHPLC-MS and -MSMS (below), and the residues were dried in a vacuum centrifuge for subsequent fractionation by preparative HPLC (below).

### Analysis of Culture Extracts by UHPLC-MS and Hierarchical Cluster Analysis

Adsorbent extracts were centrifuged (5 min, 13,000 rpm) and analyzed (injection volume 1 μL) by UHPLC-MS on a reversed phase column (2.1 × 50 mm, 1.5 μm, Thermo Accucore Vanquish RP-MS, Thermo Fisher Scientific) on an Agilent 1290 Infinity II instrument connected to a maXis Impact ESI-QTOF MS (Bruker), using a gradient of MeCN in water (20–55% MeCN in 3 min, 95% MeCN for 1.2 min, at 0.9 mL/min, with 0.2% formic acid). The MS was operated in positive mode with scanning of *m/z* 50–1500, and calibration of the mass spectra was obtained by a sodium formate clusters. The software Compass DataAnalysis 4.3 (Bruker Daltonics) was used for calibration and data analysis. Isopedopeptins were identified by comparison with authentic compounds from previous work (Nord et al., 2020), and identification of other compounds was attempted by comparison of HRMS-data with databases (Antibase and Combined Chemical Dictionary). UHPLC-MSMS was run on the same instrument, column and UHPLC-conditions, on extracts or on extracts treated with 2% NaOMe in MeOH for 30 min at room temperature, using the auto MSMS function (1+, 2+, and 3+ precursor ions, *m/z* 200–1100, ramped fragmentation energies: 1+: 30/40/45 eV for *m/z* 100/500/1000; 2+: 15/25/30 eV for *m/z* 100/500/1000; 3+: 10/20/25 eV for *m/z* 100/500/1000). Following conversion of UHPLC-MS data to mzXML format using DataAnalysis 4.3, ion-chromatogram peak picking in the range 20–200 s was done by the program XCMS in the software environment R using the centWave method (peakwidth 3–20 s, *m/z* tolerance 5 ppm, noise 10,000) (Smith et al., 2006; Tautenhahn and Böttcher, 2008). XCMS was used for subsequent peak grouping and missing peak filling. The resulting peak-areas of the molecular features of each sample were normalized against the sum of peak-areas, and the resulting relative peak-areas were then 10-logarithmized. To reduce the complexity of the data set, only the dominant molecular feature for each molecule was used, and features with peak-areas <200,000 were not included. The data were used to construct a heat map with hierarchical clustering of both strains and molecular features (distance: Euclidean, clustering: complete).

### Fractionation of Culture Extracts by Preparative HPLC

Based on the UHPLC-MS analyses and the heat map data, representative isolates were selected for fractionation and subsequent bioassays. The dried culture extracts were dissolved in 1 mL 50% MeCN and following centrifugation (13,000 rpm for 5 min), 1-mL samples were fractionated on a reversed phase preparative HPLC column (Luna Omega PS C18, 21.2 × 100 mm, 5 μm), eluted with a gradient of MeCN in water (15–50% MeCN in 15 min, 50–95% MeCN in 2 min, and a hold at 95% MeCN for 8 min, at 10 mL/min) with 0.2% formic acid. The eluent was monitored at 210 nm and fractions (2 mL) were collected in deep-well plates (start after 5 min). Aliquots (100 μL) of all fractions were transferred to 96-well microtiter plates, dried over-night in a fume-hood, and subsequently analyzed for antibacterial activity (below).

### *In vitro* Bioassay of Antibacterial Activity

Chromatographic fractions of the extracts of selected isolates (above) were assayed for antibacterial activity using an earlier developed protocol (Thaning et al., 2001; Nord et al., 2019) with the following human pathogenic bacteria or bacteria commonly used in such bioassays: *Escherichia coli* LMG 15862, *Acinetobacter baumannii* LMG 1041^T^, *Enterobacter cloacae* LMG 2783^T^, *Klebsiella pneumoniae* LMG 20218, *Pseudomonas aeruginosa* LMG 6395, and *Staphylococcus aureus* LMG 15975, all purchased from BCCM/LMG and maintained as advised by supplier. Briefly, cell suspensions (0.1 mL/well) at a concentration of 10^4^ cells per mL half strength VBP (Oxoid Ltd.) were added to the microtiter plates with dried aliquots of HPLC fractions (above), and the plates were incubated at 37°C in darkness for 16–24 h. The effect on the bacterial growth was estimated visually according to the following scale: 3/red – full growth inhibition, 2/orange – intermediate growth inhibition, 1/yellow – weak growth inhibition, 0 – no growth inhibition. Positive controls were wells with cell suspensions in VPB only, and sterile VPB was used as negative control.

## Results

### Isolation of MDR *Pedobacter* Strains

A total of 28 MDR strains of the genus *Pedobacter* were isolated from twelve soil samples collected across Sweden (Table 1), using an antibiotics resistance-based approach. Twenty-two of the isolates were closest related to *P. cryoconitis*, whereas three were closest to *P. lusitanus* and three to *P. steynii*.

First, seven MDR *Pedobacter* strains were isolated from soil samples 1–4 and 9, mainly using a triple combination of the antibiotics NAL (quinolone/DNA gyrase inhibitor), AMP (β-lactam/cell wall synthesis inhibitor) and KAN (aminoglycoside/protein synthesis inhibitor) (Table 1, #1). These isolates were closest related to *P. cryoconitis* (five strains), *P. steynii* (one strain), and *P*. *lusitanus* (one strain) and corresponded to approximately 3% of the isolates obtained in this isolation round. *P. cryoconitis* UP508, isolated from soil sample 1, was found to be able to grow in the presence of the following ten antibiotics, one at the time, at the concentration of 20 mg/L: AMP, CIP, ERY, GEN, KAN, LIN, NAL, POL, STR, and VAN.

Nine additional strains of *Pedobacter* spp. were then isolated from soil samples 6–8 and 11, again by means of selection for antibiotic resistance using various combinations of DNA gyrase inhibitors/cell wall synthesis inhibitors and/or protein synthesis inhibitors (Table 1, #2). Eight of the isolates were *P. cryoconitis* and one was *P. lusitanus*.

**FIGURE 1:**
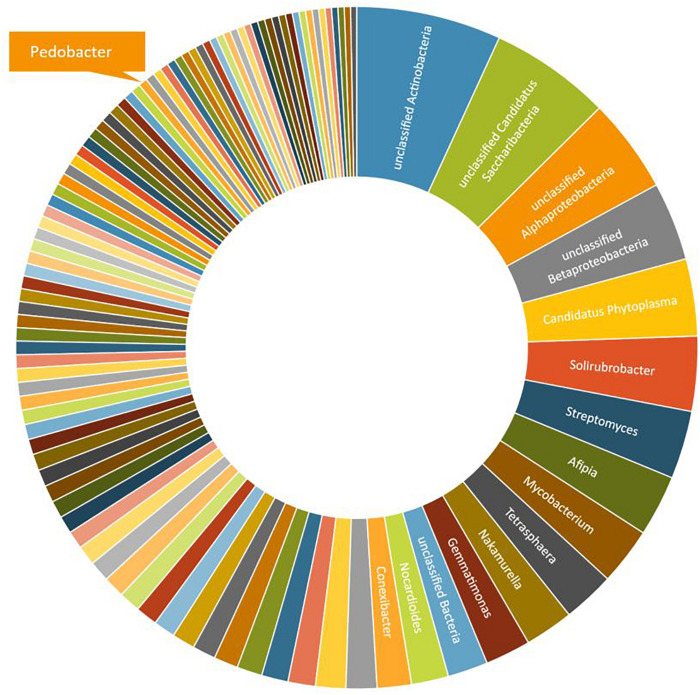
A graphical representation of the relative abundance of the one hundred most common microbial OTUs (operational taxonomic units) at genus level in sample 10 (Table 1). These OTUs correspond to 76% of the total number of reads. The indicated slice representing the genus *Pedobacter* represents 0.32% or 93 individual reads out of 29,122.

A third round of *Pedobacter* isolation, using additional combinations of antibiotics, was applied on soil samples 5, 10, and 12 (Table 1, #3). This isolation attempt resulted in eleven more isolates of *Pedobacter* (Table 1, #3), which was >30% of the 30 isolates obtained in total in this round, whereas no *Pedobacter* spp. isolates were obtained from control isolation plates. Eight of the isolates were *P. cryoconitis*, two *P. steynii*, and one *P. lusitanus* (Table 1, #3). NGS data from soil sample 10 showed that the genus *Pedobacter* was among the hundred most common genera (rank 69) with a relative abundance of 0.32% of the total number of bacterial sequences (Figure 1 and Supplementary Table 2). The two most common operational taxonomic units were unclassified *Actinobacteria* (5.21%) and unclassified *Candidatus Saccharibacteria* (4.36%).

**FIGURE 2:**
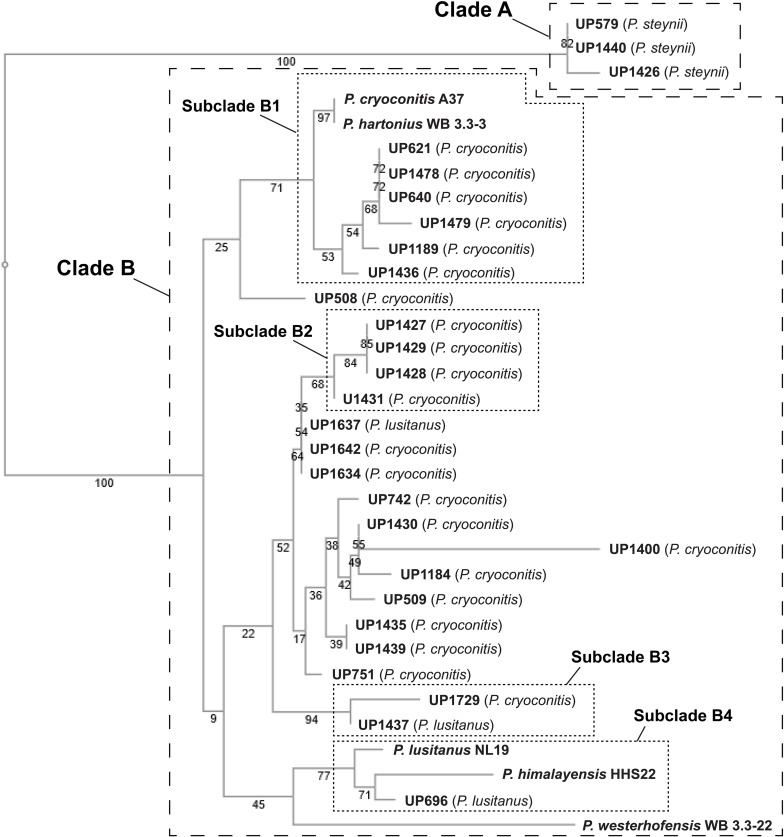
NJ tree (Jukes–Cantor/bootstrap 1000) based on 16S rRNA gene sequence analysis showing the phylogenetic relationship between the isolated *Pedobacter* strains and five closely related species of *Pedobacter* that are available from culture collections (GenBank accession numbers MW332350 to MW332382). The online multiple alignment program for amino acid or nucleotide sequences, MAFFT version 7, was used for the alignment and subsequent tree building (Katoh et al., 2019).

A phylogenetic tree was constructed based on 16S rRNA sequence data obtained from the isolated MDR *Pedobacter* strains, along with sequences of five purchased reference strains (Figure 2). The isolates UP579, UP1426, and UP1440, identified as closest to *P. steynii*, separate from the rest of the strains into clade A whereas all other isolates cluster in the major clade B, which in turn divides into several subclades, and four of these have bootstrap support ∼70 or better (subclade B1–B4). One of these subclades (B1) harbors UP621, UP640, UP1189, UP1189, UP1478, and UP1489, along with the reference isolates *P. cryoconitis* A37 and *P. hartonius* WB 3.3-3. Another subclade (B2) includes UP1427, UP1428, UP1429, and UP1431 (all closest to *P. cryoconitis*), and two further subclades (B3 and B4) contain UP1437 (*P. lusitanus*) and UP1729 (*P. cryoconitis*), and UP696 (*P. lusitanus*), *P. lusitanus* NL19 and *P. himalayensis* HHS22, respectively. The bootstrap support for the clustering of the remaining isolated *Pedobacter* strains (10 closest to *P. cryoconitis* and 1 closest to *P. lusitanus*) into subclades, is too weak for further detailed consideration.

### Metabolites Produced by Isolated MDR *Pedobacter* Strains

**FIGURE 3:**
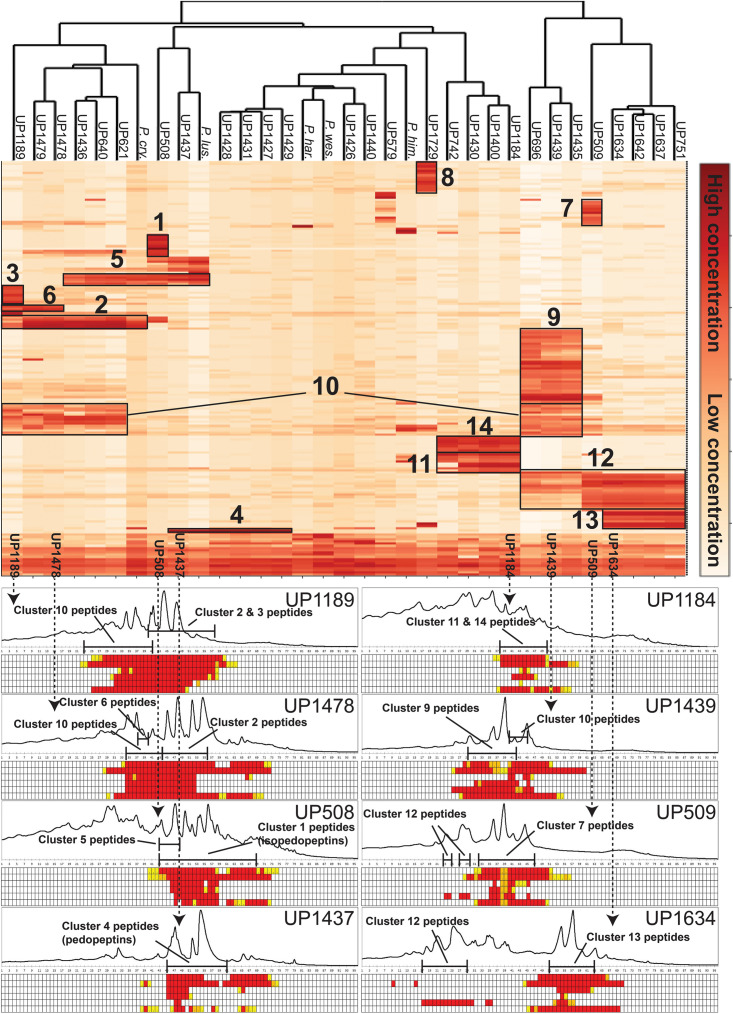
Top: Heat map generated by hierarchical clustering (Euclidean, complete) of major compounds (rows) produced by 33 strains of *Pedobacter* (columns) as analyzed by UHPLC-MS. Numbers indicate clustering of compounds discussed in the text. [UP-strains are from this work. *P. cry.*, *Pedobacter cryoconitis* A37 (LMG 21415^T^); *P. lus.*, *P. lusitanus* NL19 (LMG 29220^T^); *P. har.*, *P. hartonius* WB 3.3-3 (DSM 19033^T^); *P. wes.*, *P. westerhofensis* WB 3.3-22 (DSM 19036^T^); *P. him*., *P. himalayensis* HHS22 (JCM 12171^T^).] Bottom: Chromatograms (A210) from HPLC-fractionation of the indicated *Pedobacter* isolates, with data from bioassays against a panel of bacterial pathogens under each chromatogram. Bacteria are shown in rows (from top to bottom: *E. coli, A. baumannii*, *E. cloacae*, *K. pneumoniae*, *P. aeruginosa*, and *S. aureus*) and HPLC fractions in columns. Activity grading of fractions is ranging from red (full inhibition) to white (no visible inhibition). The presence of peptides from clusters 1–14 is indicated in each chromatogram.

Culture extracts of the 28 isolated MDR *Pedobacter* strains, along with extracts of five reference isolates, were analyzed by UHPLC-MS and –MSMS and HCA. The 33 strains were shown to have widely differing production of metabolites and many of the substances gave rise to ions with *m/z* values and charge states (+1 to +4) suggesting them to be peptides. A heat map (Figure 3) was constructed by HCA of the 187 major compounds (Supplementary Table 3), which resulted in several well-defined clusters of compounds (Figure 3). Approximately 50% of the 187 compounds gave no relevant hits in the databases Antibase or Dictionary of Natural Products, suggesting many compounds to be new chemical entities.

**FIGURE 4:**
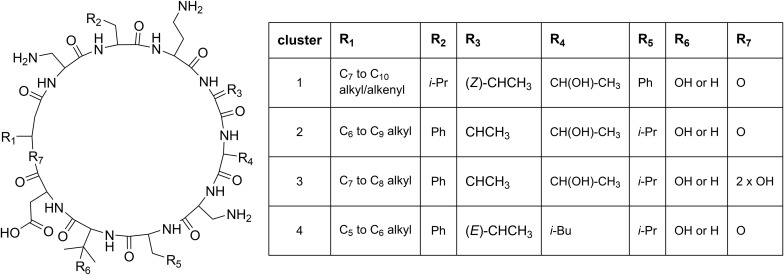
Proposed general structures of peptides in clusters 1–4 (Figure 3), based on MSMS-data obtained on peptides treated with NaOMe in MeOH. Peptides with R_7_ = 2 × OH are linear, and MSMS-data on these were obtained without NaOMe treatment. The cluster 1 peptides includes the previously published isopedopeptins (Nord et al., 2020) and the cluster 4 peptides the pedopeptins (Hirota-Takahata et al., 2014). Pedopeptin A and C were only found in cultures of two strains but are included in the cluster 4 entries.

Cluster 1 in the heat map (Figure 3) contains the recently described isopedopeptins A–F (Figure 4; Nord et al., 2020), along with four additional analogous peptides. Isopedopeptins were only found in the isolate *P. cryoconitis* UP508.

**FIGURE 5:**
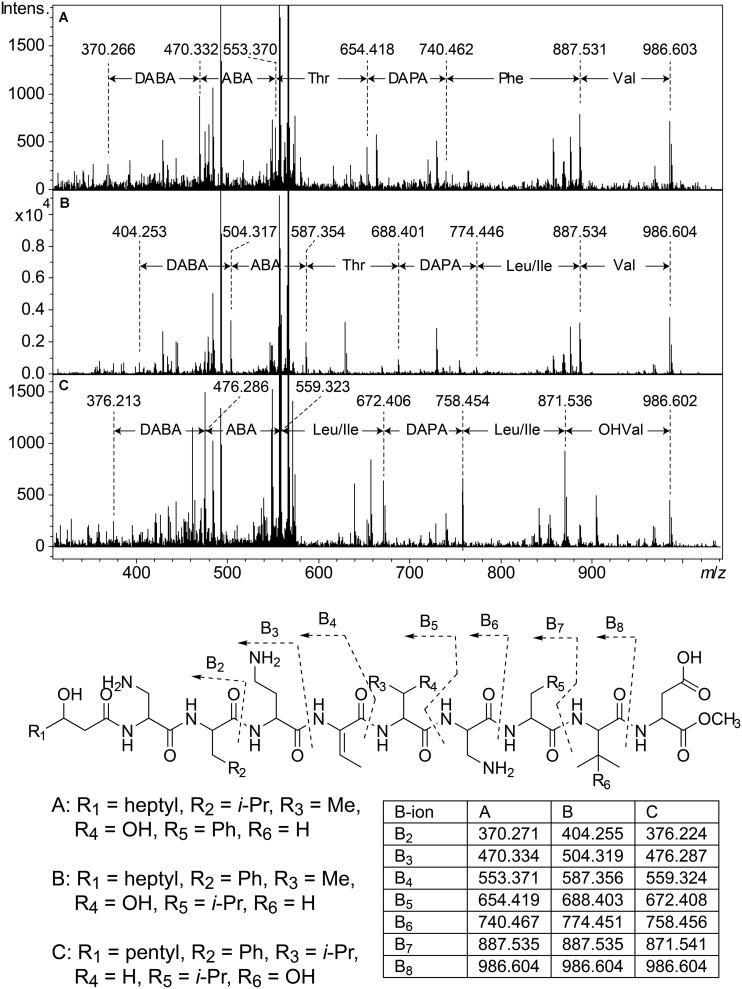
Top: MSMS spectra from analysis of three isobaric peptides from UP508 (**A**, cluster 1, isopedopeptin D), UP640 (**B**, cluster 2), and UP1437 (**C**, cluster 4, pedopeptin C), after treatment with NaOMe in MeOH. For all three peptides, the (M + 2H)^2+^ at *m/z* 567.332 was isolated and fragmented at 21.4 eV. Bottom: The resulting structures after ring-opening of the above peptides with NaOMe in MeOH, along with theoretical *m/z* values for B_2_–B_8_ fragment ions.

Cluster 2 (Figure 3) includes six substances produced by *P. cryoconitis* A37, UP621, UP640, UP1189, UP1436, UP1478, and UP1479, and five of these compounds had *m/z*-values identical to cluster 1 compounds (isopedopeptins A, B, D, E, and one uncharacterized isopedopeptin). Following lactone opening with NaOMe and MSMS analysis (Figure 5 and Supplementary Figure 2), it was concluded that these compounds indeed were similar to the isopedopeptins and the difference was that the Leu and Phe residues were on the opposite positions in the cluster 2 compounds compared to the isopedopeptins in cluster 1. Just as the isopedopeptins, these compounds contain either a Val residue or a 3-hydroxyvaline (OHVal) residue (Figure 4). Judging from bioassays made on fractionated extracts of UP1478, the cluster 2 peptides have similar antibacterial activity as the isopedopeptins (Figure 3).

The cluster 2 peptides were also produced by the strain UP1189, but in this isolate four peptides with *m/z* values in accord with cluster 2 peptides with one molecule of H_2_O added to the structures were much more abundant (Figure 3, cluster 3). These peptides, however, gave rise to abundant sequence ions in MSMS even without alkaline lactone opening, and the MSMS-data showed that these peptides were open chain variants of the cluster 2 peptides (Figure 4 and Supplementary Figure 3), explaining the observed mass difference compared to the cluster 2 peptides.

Cluster 4 in the heat map (Figure 3) encompasses a compound produced by the strains UP1427-1429, UP1431, UP1437, and *P. lusitanus* NL19 (LMG 29220 T), which produces *m/z* corresponding to a monoisotopic mass of 1098.645 Da, in line with the antibiotic cyclic lipodepsipeptide pedopeptin B (Figure 4) that previously has been shown to be produced by *Pedobacter* sp. SANK 72003 (Hirota-Takahata et al., 2014; Kozuma et al., 2014). Following ring-opening with NaOMe, the identity of pedopeptin B was verified by MSMS (Supplementary Figure 4). Pedopeptin B has a 3-hydroxy-7-methyloctanoic acid residue and a Val residue (Hirota-Takahata et al., 2014). UP1437 and *P. lusitanus* NL19 were also found to produce pedopeptin A, which is the OHVal analog of pedopeptin B, and in addition also pedopeptin C, which has a 3-hydroxyoctanoic acid residue instead of a methyl branched hydroxyfatty acid. Subsequently, cluster 4 was found to contain a pedopeptin B analog lacking a CH_2_ group in the hydroxyfatty acid part of the molecule. In analogy with pedopeptin A and C, the pedopeptin B analog was suggested to have a 3-hydroxyoctanoic acid residue instead of a 3-hydroxy-7-methyloctanoic acid residue, and was proposed the name pedopeptin D.

**FIGURE 6:**
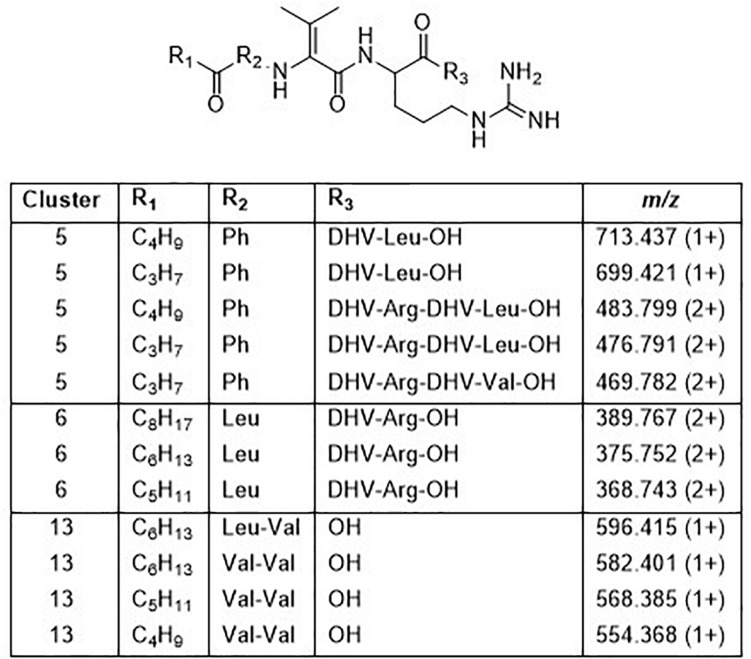
Structures of peptides in clusters 5, 6, and 13 based on MSMS and NMR analysis (Supplementary Figures 5–9 and Supplementary Tables 3, 4).

Cluster 5 contains compounds from seven isolates, and data from MSMS suggested these to be Pro containing peptides. However, NMR studies of two of these peptides showed that they did not contain Pro, but instead the isobaric amino acid dehydrovaline (DHV, 2-amino-3-methylhex-2-enoic acid, Supplementary Tables 3, 4 and Supplementary Figures 6, 7). The general structure of the cluster 5 peptides was thus concluded to be C_3_H_7_CO/C_4_H_9_CO-Phe-DHV-Arg-DHV-R, where R is different structure elements in the peptides (Figure 6 and Supplementary Figure 5). The presence of DHV in these peptides makes it likely that similar peptides from other *Pedobacter* strains, also contain this amino acid and not Pro (below). The isolates UP1189, UP1478, and UP1479, were found to produce analogous peptides, with the MSMS-derived structures C_5_H_11_CO/C_6_H_13_CO/C_8_H_17_CO-Leu/Ile-DHV-Arg-DHV-Arg (Figure 3, cluster 6; Figure 6 and Supplementary Figure 5). Further linear lipopeptides were present in cluster 13 and cluster 14. The cluster 13 peptides had *m/z* 554.368, 568.385, 582.401, and 596.415 (all 1+), which in MSMS produced complementary B- and Y-type fragment ions supporting the structures C_4_H_9_CO/C_5_H_11_CO/C_6_H_13_CO-Val-Val-DHV-Arg and C_7_H_15_CO-Leu/Ile-Val-DHV-Arg, respectively (Figure 6 and Supplementary Figure 8). The cluster 14 peptides gave in MSMS immonium ions that showed the presence of His in all peptides and Met in some of the peptides. Two of these compounds gave B-type fragment ions in line with the sequences C_5_H_11_CO/C_6_H_13_CO-His-DHV-Met-DHV-Met (Supplementary Figure 9), whereas in other peptides one or both Met residues was replaced by Leu/Ile/Val.

**FIGURE 7:**
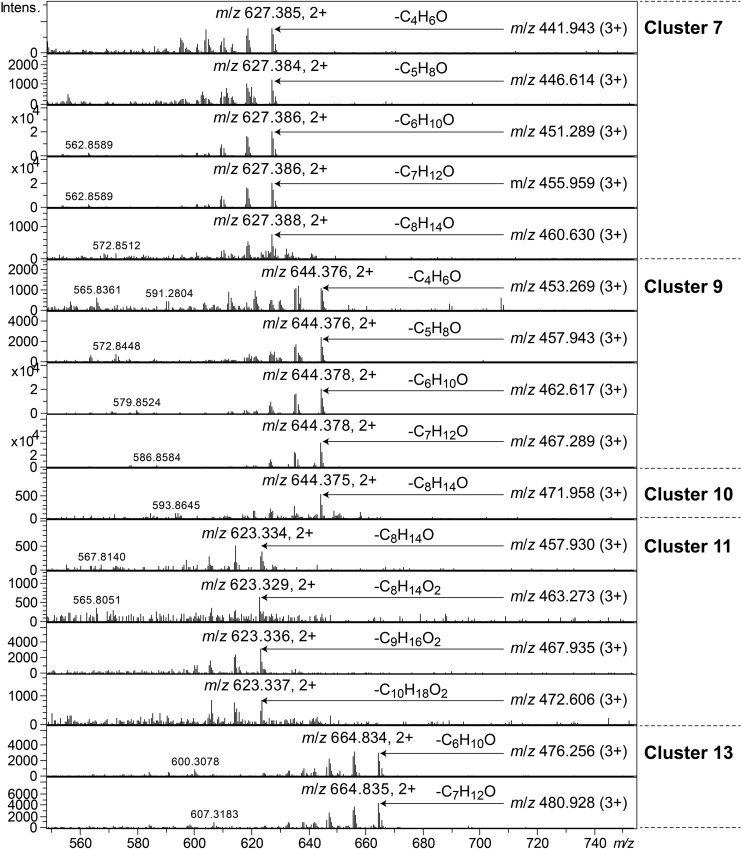
MSMS spectra of peptides from clusters 7, 9–11, and 13. The respective parent ions are shown in the top-right corner of the spectra.

Clusters 7, 9–11, and 13, were all found to contain peptides presumably similar to each other, producing 2+/3+/4+ ions corresponding to monoisotopic molecular masses in the range 1253–1440 Da. Just as the cyclic lipodepsipeptides in clusters 1, 2, and 4, these did not produce easily interpretable sequence ions in MSMS, but they did not ring-open with NaOMe treatment, which suggested them to be ring-closed by a peptide bond and not via an ester bond. The peptides of clusters 7, 9–11, and 13 all produced intense 2+ fragment ions in MSMS at *m/z* 627.387, 644.377, 644.377, 623.335, and 664.834 (Figure 7). These fragment ions are in line with elimination of fatty acyl residues (C_4__–__8_), or hydroxyl fatty acyl residues substituents (C_8__–__10_), from cyclic peptide cores (Figure 7). Peptides of these clusters were present in fractions with activity against all tested bacteria (Figure 3, bottom).

**FIGURE 8:**
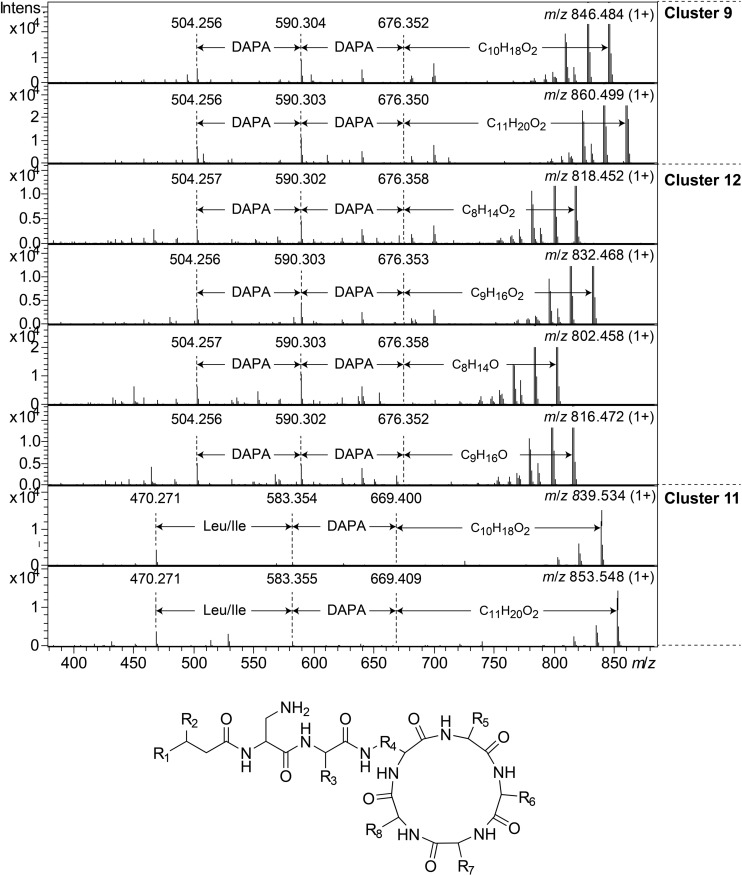
Top: MSMS spectra for representative peptides of clusters 9, 12, and 11. The respective parent ions are shown in the top-right corner of the spectra. Bottom: Suggested general structure for peptides of clusters 9, 12, and 11. R_1_, alkyl groups; R_2_, H or OH; R_3_, sidechain corresponding to either DAPA (clusters 9 and 12) or Leu/Ile (cluster 11); R_4_, sidechain corresponding to DAPA (or a homolog); R_5_–R_8_, sidechains corresponding to DAPA, ABA, Thr, and Phe (clusters 9 and 12) or DAPA, ABA, Thr, and Leu/Ile (cluster 11), in any sequence, or homologs thereof.

Clusters 9, 11, and 12, were found to include another type of peptides, presumably related to each other, with monoisotopic molecular masses in the range 785–860 Da, which gave 2+ and 3+ ions in MS. In MSMS, these peptides did not produce ions enabling facile determination of the full amino acid sequence, and they did not add methanol when treated with NaOMe, suggesting them to be ring-closed by peptide bonds. MSMS resulted in a few Y-type ions suggesting N-terminal fatty acyl groups for the peptides – C_10__–__11_ hydroxy fatty acids for clusters 9 and 11, and C_8__–__9_ fatty acids with and without hydroxyl groups for the cluster 12 (Figure 8). Judging from Y-type ions, the fatty acyl groups of the clusters 9 and 12 peptides were linked to two consecutive DAPA residues, whereas in the cluster 11 peptides the linkage was to DAPA – Leu/Ile (Figure 8). For neither of the peptides, no further Y-type ions were observed, suggesting these fatty acyl dipeptide moieties to be sidechains on cyclic peptide cores. The observed immonium ions formed by the peptides in MSMS showed the clusters 9 and 12 peptides to contain Phe, whereas the cluster 11 peptides instead contained Leu/Ile. Additionally, the clusters 9 and 12 peptides produced both 2+ and 3+ ions, whereas the cluster 11 peptides only produced 2+ ions, suggesting the presence of one additional basic amino acid, such as DAPA, in the clusters 9 and 12 peptides compared to the cluster 11 peptides. The presumed peptide core of the cluster 11 peptides gave a fragment at *m/z* 470.271 (1+), whereas the corresponding fragment for the clusters 9 and 12 peptides gave an ion at *m/z* 504.256 (1+) (Figure 8). As mentioned above, the cluster 11 peptides did not contain Phe, but instead Leu/Ile. By replacing the mass of a Leu/Ile residue in core of the cluster 11 peptides, with the mass of a Phe residue, identical masses are obtained for the core part of the structures. This strongly suggests that the cluster 11 peptides are analogs to the clusters 9 and 12 peptides with a central Phe replaced with a Leu/Ile and one DAPA in the sidechain replaced with another Leu/Ile. The only reasonable molecular formula within ±2 mDa for the fragment ions at *m/z* 470.271 (1+) and 504.256 (1+) is C_20_H_36_N_7_O_6_^+^ and C_23_H_34_N_7_O_6_^+^, respectively, which may correspond to peptide cores composed of 2 × DAPA, Thr, Leu/Ile, ABA, and 2 × DAPA, Thr, Phe, ABA, respectively, or various homologous combinations of amino acids (Figure 8). To account for the observation of 2+ and 3+ ions for the clusters 9 and 12 peptides, and 2+ ions for the cluster 11 peptides, the sidechain should be linked to one of the two DAPA residues (or homologs) in the central peptide core (Figure 8).

This type of peptides from clusters 9 and 12, was found to be prominent in fractions with activity against *P. aeruginosa*, and this was particularly apparent for cluster 12 peptides from the isolates UP509 and UP1634 (Figure 3, bottom). On the other hand, when fractions from UP1184 were screened, very little activity against *P. aeruginosa* was detected in fractions dominated by cluster 11 peptides of this type. The general difference between peptides of this type from clusters 9 and 12, compared to cluster 11, is the presence of two Leu/Ile residues in the cluster 11 peptides, instead of one DAPA and one Phe residue as in the peptides in clusters 9 and 12.

## Discussion

Twenty-two of the 28 isolated MDR *Pedobacter* strains were closest related to *P. cryoconitis*. Interestingly, similar *P. cryoconitis* isolates were obtained from soils sampled at different locations along a 1200 km line from south to north, i.e., Gotland County (sample 12 ⇒ UP1436) to Norrbotten County (soil sample 11 ⇒ UP1189), with Uppsala County (soil sample 3 ⇒ UP621 and UP640), and Gävleborg County (soil sample 8 ⇒ UP1478 and UP1479) situated in-between. These isolates all clustered in the same subclade (B1) of the 16S rRNA derived phylogenetic tree (Figure 2), and in the metabolite derived phylogenetic tree (Figure 3). This indicates that bacteria of the genus *Pedobacter* are rather widespread at levels where enrichment culturing is not needed for their detection although the actual numbers are quite low. The NGS data for soil sample 10 (0.32% *Pedobacter*) suggests it can be reasonable to expect to isolate roughly three *Pedobacter* for every thousand colonies investigated if there is no enrichment bias for antibiotic resistant *Pedobacter*. This is in sharp contrast to the eleven *Pedobacter* strains out of the 30 investigated isolates in isolation round three in this study (>30%), obtained by using certain combinations of antibiotics to increase selection pressure.

*Pedobacter cryoconitis* UP508, isolated from soil sample 1, was found to be resistant and able to grow in the presence of antibiotics from a broad range of chemical classes, and should thus be regarded as MDR. The resistance of *P. cryoconitis* A37 was earlier reported to cover most of the tested antibiotics/classes (Margesin et al., 2003; Covas et al., 2017; Viana et al., 2018). However, the resistance for *P. cryoconitis* A37 against CIP and ERY was only reported to be 4 mg/L, not 20 mg/L. Further, resistance against polymyxins was reported previously for some other species of *Pedobacter* but not for *P. cryoconitis* (Muurholm et al., 2007; Viana et al., 2018), and likewise, the resistance against LIN and NAL was not shown earlier for *P. cryoconitis*. The remainder of the *Pedobacter* sp. isolates were not tested separately for antibiotic resistance. However, just as UP508, these strains were isolated using combinations of antibiotics for selection (Table 1), and 24 of the isolates were obtained using three or more antibiotic drugs of different classes in combination and should be regarded as MDR, whereas four isolates were isolated using only two antibiotics in combination.

The rationale behind the antibiotic resistance-based isolation of *Pedobacter* strains in this study, was to find bacterial isolates producing novel antibiotic compounds, and specifically antibiotic metabolites similar to the antibiotics that these isolates are resistant to. Another resistance-based antibacterial discovery platform that harness the self-protection mechanism of antibiotic producers, but using only a single antibiotic at a time for screening, was previously described as a tool to increase the discovery rate of Actinomycetes producing novel antibiotics (Thaker et al., 2013, 2014).

Given the resistance against lipopeptides observed for *P. cryoconitis* UP508, it is not surprising that many of the isolated *Pedobacter* strains were found to produce lipopeptides. The isolate UP508 was recently described to produce isopedopeptins (Nord et al., 2020) and several other strains produced the pedopeptins (Hirota-Takahata et al., 2014), here identified by MSMS. However, a number of isolates also produced unknown compounds, including the isopedopeptin analogs present in clusters 2 and 3, here only characterized by MSMS, and linear lipopeptides (clusters 5, 6, 13, and 14), characterized by MSMS and partly by NMR (two peptides of cluster 5). The isopedopeptins, the pedopeptins and the cluster 2 peptides share some structural features with the last-resort antibiotics polymyxin B and E (colistin), i.e., they are cyclic peptides of similar size (ca 1100–1150 Da), they are cationic due to the presence of several basic amino acids, and they have a fatty acid tail. One major difference is the nature of the cyclic structure. The polymyxins have a 23-membered ring, involving seven of the ten amino acids, ring-closed via an amide bond to the γ-amino group of a 2,4-diaminobutanoic acid residue (DABA), compared to a 31-membered lactone, involving all nine amino acids and the 3-hydroxyfatty acid, for the *Pedobacter* peptides. Another difference is the presence of six DABA residues in the polymyxins, compared to one DABA and two 2,3-diaminopropanoic acid residues in the *Pedobacter* peptides, which makes the polymyxins more cationic than the *Pedobacter* peptides. With respect to antibacterial activity and mechanism of action, there are both similarities and differences between polymyxins and the *Pedobacter* peptides. Both pedopeptins and isopedopeptins have potent activity against Gram-negative bacteria (Kozuma et al., 2014; Nord et al., 2020) just as the polymyxins, which are used to treat Gram-negative bacterial infections, but the isopedopeptins have activity also against colistin resistant strains (Nord et al., 2020). Regarding the mechanism of action, the isopedopeptins were recently described to cause leakage of bacterial membranes (Nord et al., 2020), as the polymyxins and many other cationic peptides (Straus and Hancock, 2006). Additionally, the pedopeptins, as well as polymyxin B, have been described to selectively inhibit the binding of bacterial lipopolysaccharides to cellular receptors at nanomolar concentrations (Kozuma et al., 2014).

The cluster 5 peptides were shown by NMR to contain the amino acid residue DHV, which previously has been found in many non-ribosomal peptides (Sidołak, 2015). The presence of DHV in two peptides made it likely that also the other similar peptides contained DHV and not the isobaric Pro (Figure 6). Further novel lipopeptides were the cyclic peptides of clusters 7, 9–13, of which there were two different types: one larger with molecular mass 1253–1440 and one smaller with mass 785–860. These peptides were only partly characterized by MSMS and attempts to study their structures by NMR (data not shown) were not successful due to the broad NMR signals obtained from these compounds. Examples of these types of new lipopeptides were present in semi-pure antibacterial fractions (Figure 3), suggesting them to possess antibiotic properties. The discovery of several new and putative antibiotic lipopeptides from *Pedobacter* strains, isolated using combinations of several antibiotic drugs for selection, strengthens the hypothesis that this approach is a very powerful tool to obtain bacterial isolates that produce antibiotic compounds.

When comparing how the different *Pedobacter* isolates cluster in the 16S rRNA-derived phylogenetic tree (Figure 2) with the metabolite-derived HCA of the heat map (Figure 3), it is clear that the results from two different methods for systematization/comparison differ from each other. This is not surprising since one method is based on the calculations of genetic distance according to similarities in the evolutionary conserved 16S rRNA gene sequence and the other method is based on UHPLC-MS based snapshots of the phenotypic expression of the different isolates. There are several apparent similarities indicating that genetically related isolates often seem to share much of their phenotypic traits as well, but also striking differences.

Studying the phylogenetic positioning of the 22 isolated strains identified as closest to *P. cryoconitis*, gives that only six of these group in the same subclade (B1) as *P. cryoconitis* A37 (Figure 2). These isolates, UP621, UP640, UP1189, UP1436, UP1478, UP1479, and *P. cryoconitis* A37 (LMG 21415), also cluster phenotypically (Figure 3). MSMS suggests these isolates to produce cyclic lipodepsipeptides (Figure 3, cluster 2) similar to the isopedopeptins, but differing by having Phe_2_ and Leu_7_ in their structures instead of Leu_2_ and Phe_7_ as in the isopedopeptins. The only isolate producing isopedopeptins, UP508 (Figure 3, cluster 1), is on a separate branch in the phylogenetic tree (Figure 2).

In the 16S rRNA based phylogenetic tree (Figure 2), *P. hartonius* WB3.3-3 is placed in the same subclade (B1) as the clusters 2 and 3 peptide producing isolates, but it is more distant in the metabolite derived clustering of the heat map (Figure 3). Perhaps, the lipodepsipeptide biosynthetic gene complex (BGC) is present in *P. hartonius* WB3.3-3 as well, but is not active under the cultivation conditions of the study. Further, when closely inspecting this subclade (B1), the isolates UP621 and UP640 originate from the same soil sample and are indistinguishable from each other on the level of 16S rRNA gene (Figure 2), but there are still some differences when comparing their metabolite profiles (Figure 3). If these differences are based on an actual strain variation of two very closely related but distinct bacterial strains or just fluctuations in the metabolite expression levels from two genetically identical clones isolated from the same soil sample is difficult to establish without closer examination of these isolates.

The isolates UP1427, UP1428, UP1429, and UP1431 (all closest to *P. cryoconitis*), which form another subclade (B2) in the 16S rRNA derived phylogenetic tree (Figure 2), cluster also in the metabolite derived tree (Figure 3). These strains produce the related pedopeptins (Leu_5_ instead of Thr_5_ compared to the cluster 2 peptides, Figure 3), together with the isolates UP1437 (closest to *P. lusitanus*) and *P. lusitanus* NL19, which cluster in two different subclades (B3 and B4, respectively). However, UP1427, UP1428, UP1429, and UP1431 only produce pedopeptin B and D (both with Val_8_), whereas UP1437 and *P. lusitanus* NL19 in addition also synthesize pedopeptin A and C (both with OHVal_8_). Another difference between the pedopeptin producing isolates, is that UP1437 and *P. lusitanus* NL19 produce cluster 5 peptides (linear lipopeptides, Figure 6) in contrast to the other isolates.

There are also striking differences when the 16S rRNA and metabolite derived phylogenetic trees are compared. UP1437 and UP1729 cluster in the 16S rRNA tree (Figure 2, subclade B3), but the metabolite production appears to differ widely (Figure 3). Further, UP696 group with the reference isolates *P. lusitanus* NL19 and *P. himalayensis* HHS22 (Figure 2, subclade B4) but exhibit very different metabolite profiles (Figure 3). These differences can depend on the presence or absence of certain gene complexes and/or by the expression of such genes under the specific culturing conditions.

Phylogenetic positioning of the remaining isolates identified as *P. cryoconitis* and *P. lusitanus* follows, in general but not completely, their metabolic clustering. For example, the producers of clusters 11 and 14 peptides, i.e., UP742, UP1184, UP1400, and UP742 (Figure 3), cluster with UP509 in the 16S rRNA derived tree (Figure 2), albeit with rather low bootstrap support. However, UP509 appears to have a very different production of metabolites (Figure 3). Other examples are the clusters 12 and 13 peptide producers UP751, UP1634, UP1637, and UP1642 (Figure 3), of which the three latter cluster separated from UP751 in the 16S rRNA tree (Figure 2), and the cluster 9 peptide producers UP696, UP1435, and UP1439, of which UP696 cluster separated from the other isolates in the subclade B4 of the16S rRNA tree (Figure 2).

Another discrepancy between phylogenetic and metabolic clustering is the positioning of the isolates UP696, UP1437, and UP1637 identified as closest to *P. lusitanus* (Table 1) in comparison to the reference isolate *P. lusitanus* NL19. UP1437 and *P. lusitanus* NL19 produce very similar patterns of metabolites and cluster together in the metabolite based phylogenetic tree (Figure 3). In contrast, UP696 and UP1637 produce two different sets of compounds and cluster separated from each other and far from UP1437 and *P. lusitanus* NL19. UP696 clusters with UP1435 and UP1439 (both closest to *P. cryoconitis*) and UP1637 with UP1634 and UP1642 (both closest to *P. cryoconitis*). In the 16S rRNA derived tree, UP696 clusters with the reference strain *P. lusitanus* NL19 (subclade B4), whereas UP1437 is in subclade B3 together with UP1729 (closest to *P. cryoconitis*) and UP1637 is clustering (lower bootstrap support) with the metabolically similar UP1634 and UP1642 (both closest to *P. cryoconitis*). Sequencing of the whole genome of all *Pedobacter* isolates, combined with additional biochemical analysis as well as including more *Pedobacter* reference isolates, may be necessary to resolve some of the taxonomic ambiguities noted above.

The phylogenetic tree based on sequence comparisons of one gene only, here the 16S rRNA gene, is obviously a much blunter instrument than an examination of the expressed part of the whole genome. For example, isolates UP579, UP1426, and UP1440, identified as closest to *P. steynii*, taxonomically are all placed in the well-defined clade A (Figure 2), but phenotypically cluster close to the reference isolates of *P. hartonius* WB 3.3-3, *P. westerhofensis* WB 3.3-22, and *P. himalayensis* HHS22 (Figure 3) that all belong to the major phylogenetic clade B. A feature shared by all these isolates is the rather low production of metabolites under the conditions applied in our study (Figure 3), which is probably why these taxonomically distant species cluster together in the metabolite derived phylogenetic tree.

Bacterial peptides partly composed of non-proteinogenic amino acids, such as the isopedopeptins and pedopeptins, and the similar peptides of clusters 2 and 3, are likely to be products from multi-modular non-ribosomal peptide synthases (NRPSs), coded for by a BGC. Based on the phylogenetic tree, it is possible that a strain carrying an ancestral *Pedobacter* BGC was subjected to a mutation causing a change in specificity for one adenylation domain of the NRPS from Leu_5_ to Thr_5_ (or reversed), leading to the division of strains into the pedopeptin-type producers and the cluster 2 type peptide producers. Subsequently, it appears as if one strain of the cluster 2 type producers was subjected to a BGC module rearrangement leading to the production of peptides with Leu/Phe positional exchange, leading to isopedopeptins as observed in strain UP508 (cluster 1). The strain UP1189 produces cyclic lipodepsipeptides as the rest of the cluster 2 isolates, but also linear versions of the same peptides. UP1189 is on a separate branch of the 16S rRNA phylogenetic tree compared to most of the cluster 2 peptide producers. The BGC of UP1189 may have been subjected to a mutation in the thioesterase (TE) domain of the NRPS, leading to a less efficient cyclization accompanied by release of the peptides from the NRPS. Hypothetically, the TE of the UP1189 NRPS may allow water to a greater extent compete with the hydroxyl function of the 3-hydroxyfatty acid, for performing the nucleophilic addition to the NRPS linked carbonyl of the growing peptide, resulting in the release of linear peptides from the NRPS.

The pedopeptin B and D producing strains (UP1427, UP1428, UP1429, and UP1431) cluster (subclade B2) separated from the pedopeptin A–D producing UP1437 (subclade B3) and *P. lusitanus* NL19 (subclade B4). Pedopeptin B and D contain a Val_8_ residue which is replaced by OHVal_8_ in pedopeptin A and C, and the OHVal is likely to be formed by oxidation of Val after incorporation into the growing peptide chain in the NRPS. The observed clustering suggests there was an ancestral strain with the original pedopeptin producing BGC, which suffered mutations in a monooxygenase in this BGC. This resulted in strains incapable of oxidation of Val to OHVal and thus only producing pedopeptin B and D, in parallel to strains with the ability to produce pedopeptin A–D.

There is an apparent risk of isolating the same bacterial clone more than once from the same soil sample. Possible examples here are the isolates UP1427, UP1428, UP1429, and UP1431, which were isolated from the same soil sample (sample 10), and are very similar to each other both genetically and metabolically. To the contrary, there are also many isolates originating from the same soil sample that instead cluster with other isolates. For example, both UP508 and UP509 were derived from soil sample 1, but cluster far away from each other, and the same is true for UP742 and UP751 that originated from sample 9. Considering all the isolates coming from the same soil samples and that are noticeably similar to each other, both phylogenetically and metabolically, then a rough estimate is that around 25% of the isolates in this study may (or may not) be due to duplicate isolation events. This uncertainty may be settled by sequencing the whole genome of each isolate or by developing multi-locus sequence typing tools for this genus, but since this was not within the scope of our study we have thus chosen to include all of our isolates in the study of metabolite profiles of the *Pedobacter* isolates. However, what appears to be a general observation is that isolates show more variation in their metabolite profiles if they originate from distinct soil samples.

## Conclusion

The research described here shows the great potential of using an antibiotic selection approach for finding novel and interesting bacterial isolates, in extremely complex sample matrices full of non-interesting or previously known microorganisms, with the goal to find new antibiotic compounds. Many aspects of the here tentatively described *Pedobacter* peptides are awaiting deeper studies, but our study clearly shows that the members of this genus might be accessed from different environmental samples and that they are of high value in screening attempts for new antimicrobials originating from nature. As of yet, we have focused on the peptides of cluster 1, produced by *P. cryoconitis* UP508 (Nord et al., 2020), and these peptides are the basis for a recently granted Swedish Patent (Öberg et al., 2020) dealing with peptide antibiotics of medical interest. Furthermore, the similar but still different peptides found to be produced by different *Pedobacter* strains, make this genus a promising system for studying the evolution of antibacterial peptides.

## Data Availability Statement

The datasets presented in this study can be found in online repositories. The 16S rRNA sequences are deposited at GenBank (accession numbers MW332350 to MW332382), and the UHPLC-MS data is deposited at MetaboLights (Study Identifier MTBLS2325). Further enquires can be directed to the authors.

## Author Contributions

JB, JJL, CN, BG, BÖ, and AB designed the study. JB, JJL, CN, and AB did the laboratory work. JB and AB wrote the first manuscript draft. All authors contributed to the subsequent editing of the manuscript. All authors have given approval to the final version of the manuscript.

## Conflict of Interest

JB and JJL were employed by Ultupharma AB during the end of this study, and BÖ is CEO of Ultupharma AB. The authors declare that this study received funding from Ultupharma AB. The funder, via BÖ, JB, and JJL, was involved in the study design, laboratory work, and manuscript writing. All authors are shareholders of Ultupharma AB that owns the IP-rights to the clusters 1–3 peptides. Ultupharma AB approved the publication of the data.

## Footnotes

1 http://www.bacterio.net/pedobacter.html

2 https://blast.ncbi.nlm.nih.gov/Blast.cgi

3 https://mafft.cbrc.jp/alignment/server
