# Supplementary material for: Selective Isolation of Multidrug-Resistant Pedobacter spp., Producers of Novel Antibacterial Peptides
Source: Front Microbiol. 2021 Feb 25;12:642829. doi: 10.3389/fmicb.2021.642829 (PMC7947920; doi:10.3389/fmicb.2021.642829)
Supplement: Supplementary file 1 [file Data_Sheet_1.docx]

Supplementary Material

###

### Next generation sequencing and analysis of microbial community composition (Table 1).

### Next generation sequencing and microbial community composition was done by BaseClear BV (Leiden, The Netherlands). In short, single-end or paired-end sequence reads were generated using the Illumina NovaSeq 6000 or MiSeq system. FASTQ read sequence files were generated using bcl2fastq2 version 2.18. Initial quality assessment was based on data passing the Illumina Chastity filtering. Subsequently, reads containing PhiX control signal were removed using an in-house filtering protocol. In addition, reads containing (partial) adapters were clipped (up to a minimum read length of 50 bp). The second quality assessment was based on the remaining reads using the FASTQC quality control tool version 0.11.5. Paired-end sequence reads were collapsed into so-called pseudoreads using sequence overlap with USEARCH version 9.2.^[[1]](#footnote-1)^ Classification of these pseudoreads was performed based on the results of alignment with SNAP version 1.0.23^[[2]](#footnote-2)^ against the RDP database^[[3]](#footnote-3)^ for bacterial organisms, while fungal organisms are classified using the UNITE ITS gene database.^[[4]](#footnote-4)^

### Supplementary Table 1. A list of the relative abundance of the one hundred most common OTUs at genus level in sample 10. These one hundred OTUs correspond to 76 % of the total number of reads. *Pedobacter* is at 69^th^ place and represents 0.32 % or 93 individual reads of 29122.

|  | **Genus level of OTU** | **Number of reads** | **Relative abundance** |
| --- | --- | --- | --- |
| 1 | unclassified Actinobacteria | 1517 | 5.21% |
| 2 | unclassified Candidatus Saccharibacteria | 1269 | 4.36% |
| 3 | unclassified Alphaproteobacteria | 991 | 3.4% |
| 4 | unclassified Betaproteobacteria | 824 | 2.83% |
| 5 | Candidatus Phytoplasma | 808 | 2.77% |
| 6 | Solirubrobacter | 784 | 2.69% |
| 7 | Streptomyces | 714 | 2.45% |
| 8 | Afipia | 643 | 2.21% |
| 9 | Mycobacterium | 602 | 2.07% |
| 10 | Tetrasphaera | 543 | 1.86% |
| 11 | Nakamurella | 510 | 1.75% |
| 12 | Gemmatimonas | 476 | 1.63% |
| 13 | unclassified Bacteria | 422 | 1.45% |
| 14 | Nocardioides | 389 | 1.34% |
| 15 | Conexibacter | 359 | 1.23% |
| 16 | Bradyrhizobium | 323 | 1.11% |
| 17 | unclassified Nocardioidaceae | 317 | 1.09% |
| 18 | Marmoricola | 287 | 0.99% |
| 19 | Unclassified | 278 | 0.95% |
| 20 | Sphingomonas | 266 | 0.91% |
| 21 | Acidobacterium | 254 | 0.87% |
| 22 | Pseudomonas | 243 | 0.83% |
| 23 | Clostridium | 240 | 0.82% |
| 24 | Micromonospora | 229 | 0.79% |
| 25 | Gemmata | 229 | 0.79% |
| 26 | Dehalogenimonas | 226 | 0.78% |
| 27 | Rhizobacter | 223 | 0.77% |
| 28 | Paraburkholderia | 217 | 0.75% |
| 29 | unclassified Gammaproteobacteria | 207 | 0.71% |
| 30 | Rhizomicrobium | 188 | 0.65% |
| 31 | Parviterribacter | 184 | 0.63% |
| 32 | Devosia | 181 | 0.62% |
| 33 | Rhizobium | 178 | 0.61% |
| 34 | Kouleothrix | 177 | 0.61% |

| **Supplementary Table 1.** Continued. | | | |
| --- | --- | --- | --- |
|  | **Genus level of OTU** | **Number of reads** | **Relative abundance** |
| 35 | Hydrogenispora | 173 | 0.59% |
| 36 | Nitrobacter | 161 | 0.55% |
| 37 | Cellulomonas | 158 | 0.54% |
| 38 | Mucilaginibacter | 151 | 0.52% |
| 39 | Actinoplanes | 146 | 0.5% |
| 40 | Flavobacterium | 145 | 0.5% |
| 41 | Ferruginibacter | 144 | 0.49% |
| 42 | Labrys | 143 | 0.49% |
| 43 | Chthoniobacter | 143 | 0.49% |
| 44 | Salinibacterium | 139 | 0.48% |
| 45 | unclassified Planctomycetales | 137 | 0.47% |
| 46 | Zavarzinella | 135 | 0.46% |
| 47 | unclassified Deltaproteobacteria | 134 | 0.46% |
| 48 | Luteimicrobium | 134 | 0.46% |
| 49 | Holophaga | 133 | 0.46% |
| 50 | Ilumatobacter | 132 | 0.45% |
| 51 | Arthrobacter | 132 | 0.45% |
| 52 | Rhodococcus | 126 | 0.43% |
| 53 | Steroidobacter | 125 | 0.43% |
| 54 | Geobacter | 124 | 0.43% |
| 55 | unclassified Planctomycetaceae | 123 | 0.42% |
| 56 | Phenylobacterium | 123 | 0.42% |
| 57 | Variovorax | 122 | 0.42% |
| 58 | Pseudonocardia | 117 | 0.4% |
| 59 | Kineosporia | 116 | 0.4% |
| 60 | Candidatus Solibacter | 113 | 0.39% |
| 61 | Mesorhizobium | 113 | 0.39% |
| 62 | Pseudarthrobacter | 111 | 0.38% |
| 63 | unclassified Actinomycetales | 110 | 0.38% |
| 64 | Hyphomicrobium | 110 | 0.38% |
| 65 | Jatrophihabitans | 109 | 0.37% |
| 66 | Flavitalea | 106 | 0.36% |
| 67 | Rhodopseudomonas | 100 | 0.34% |
| 68 | Nitrosospira | 100 | 0.34% |
| 69 | Pedobacter | 93 | 0.32% |
| 70 | Acidimicrobium | 93 | 0.32% |
| 71 | Terrimonas | 89 | 0.31% |
| 72 | Leptothrix | 88 | 0.3% |
| 73 | Chloroflexus | 86 | 0.3% |
| 74 | Acidothermus | 84 | 0.29% |
| 75 | Gemmatirosa | 83 | 0.29% |
| 76 | Dactylosporangium | 83 | 0.29% |
| **Supplementary Table 1.** Continued. | | | |
|  | **Genus level of OTU** | **Number of reads** | **Relative abundance** |
| 77 | Aeromicrobium | 83 | 0.29% |
| 78 | Pedosphaera | 81 | 0.28% |
| 79 | Thermoflavimicrobium | 77 | 0.26% |
| 80 | Povalibacter | 77 | 0.26% |
| 81 | Frankia | 77 | 0.26% |
| 82 | Dongia | 76 | 0.26% |
| 83 | Phycisphaera | 75 | 0.26% |
| 84 | Granulicella | 75 | 0.26% |
| 85 | Natranaerobaculum | 75 | 0.26% |
| 86 | Dokdonella | 75 | 0.26% |
| 87 | Paenibacillus | 75 | 0.26% |
| 88 | Oligotropha | 74 | 0.25% |
| 89 | Rubrobacter | 73 | 0.25% |
| 90 | Massilia | 72 | 0.25% |
| 91 | Rudaea | 70 | 0.24% |
| 92 | Lysinimonas | 69 | 0.24% |
| 93 | Sphingobium | 69 | 0.24% |
| 94 | Gaiella | 68 | 0.23% |
| 95 | Ferrimicrobium | 67 | 0.23% |
| 96 | Kineococcus | 67 | 0.23% |
| 97 | Cryocola | 66 | 0.23% |
| 98 | Chondromyces | 66 | 0.23% |
| 99 | Aquicella | 66 | 0.23% |
| 100 | Glutamicibacter | 65 | 0.22% |

### Supplementary Table 2. UHPLC-MS data used for hierarchical clustering of the 33 *Pedobacter* strains. Peak-picking was done using XCMS. or each compound only the most dominant feature was used and additional adduct/isotope ions were manually removed. The 187 molecular features shown had peak area>200 000, and for each isolate, the peak area was normalized against the sum of peak areas and then 10-logarithmized (the “-“ has been omitted for all values in the table to save space). The cluster number (1-14) indicated for most molecular features refers to the cluster numbers of Figure 3.

| **Feature** | **Cluster** | **Charge** | ***Pedobacter* sp. strain** | | | | | | | | | | | | | | | | | | | | | | | | | | | | | | | | |
| --- | --- | --- | --- | --- | --- | --- | --- | --- | --- | --- | --- | --- | --- | --- | --- | --- | --- | --- | --- | --- | --- | --- | --- | --- | --- | --- | --- | --- | --- | --- | --- | --- | --- | --- | --- |
|  |  |  | **DSM19033** | **DSM19036** | **JCM2171** | **LMG21415** | **LMG29220** | **UP508** | **UP509** | **UP579** | **UP621** | **UP640** | **UP696** | **UP742** | **UP751** | **UP1184** | **UP1189** | **UP1400** | **UP1426** | **UP1427** | **UP1428** | **UP1429** | **UP1430** | **UP1431** | **UP1435** | **UP1436** | **UP1437** | **UP1439** | **UP1440** | **UP1478** | **UP1479** | **UP1634** | **UP1637** | **UP1642** | **UP1729** |
| mz409.73/20.1s | 12 | 2 | 4.71 | 5.20 | 5.10 | 4.94 | 5.32 | 5.38 | 1.93 | 5.08 | 5.88 | 5.62 | 2.50 | 5.37 | 2.24 | 5.32 | 5.93 | 5.45 | 5.04 | 5.14 | 4.85 | 5.13 | 5.34 | 5.41 | 3.07 | 4.93 | 5.51 | 2.73 | 4.72 | 5.38 | 5.01 | 1.94 | 1.92 | 1.89 | 5.03 |
| mz267.485/20.3s | 12 | 3 | 5.42 | 5.20 | 5.43 | 4.95 | 5.93 | 5.29 | 2.18 | 4.99 | 5.88 | 5.62 | 2.75 | 5.84 | 2.48 | 5.17 | 5.86 | 5.81 | 5.04 | 5.37 | 5.32 | 4.73 | 4.86 | 5.41 | 3.30 | 5.46 | 5.12 | 2.99 | 5.31 | 5.38 | 5.38 | 2.15 | 2.16 | 2.11 | 5.03 |
| mz453.344/20.3s | - | 1 | 2.02 | 1.30 | 1.74 | 1.38 | 2.17 | 1.78 | 2.09 | 1.31 | 2.07 | 2.01 | 2.21 | 1.56 | 1.82 | 1.40 | 2.11 | 1.80 | 1.37 | 1.54 | 1.24 | 1.46 | 1.03 | 1.53 | 2.09 | 1.90 | 1.99 | 2.12 | 1.00 | 1.91 | 1.87 | 2.00 | 1.95 | 2.19 | 2.06 |
| mz362.681/20.7s | 9 | 2 | 5.42 | 5.20 | 5.27 | 5.08 | 5.43 | 5.38 | 5.22 | 5.40 | 5.88 | 5.20 | 2.16 | 5.19 | 4.99 | 5.65 | 5.72 | 5.57 | 5.04 | 5.37 | 5.44 | 5.24 | 5.05 | 5.41 | 1.83 | 5.46 | 5.51 | 1.86 | 4.37 | 5.38 | 5.38 | 4.85 | 4.73 | 4.42 | 5.21 |
| mz633.313/20.8s | - | 1 | 4.13 | 3.90 | 4.18 | 3.13 | 5.28 | 4.67 | 5.28 | 5.14 | 3.66 | 3.47 | 5.47 | 4.76 | 4.58 | 4.20 | 0.81 | 4.54 | 3.82 | 4.96 | 4.06 | 4.55 | 4.22 | 4.38 | 5.33 | 3.36 | 4.64 | 5.92 | 4.26 | 3.11 | 3.22 | 5.04 | 4.87 | 4.98 | 4.93 |
| mz245.129/21.7s | - | 1 | 1.90 | 1.28 | 1.79 | 1.57 | 2.21 | 1.90 | 2.53 | 1.79 | 2.37 | 2.23 | 2.84 | 2.05 | 2.18 | 1.81 | 2.62 | 2.09 | 1.71 | 1.86 | 2.03 | 1.98 | 1.56 | 1.76 | 2.15 | 2.17 | 2.46 | 2.26 | 1.24 | 2.11 | 1.95 | 2.25 | 2.19 | 2.18 | 2.18 |
| mz408.213/21.8s | - | 1 | 3.31 | 2.71 | 1.88 | 1.71 | 2.59 | 2.29 | 2.39 | 2.41 | 3.50 | 3.55 | 2.70 | 2.24 | 2.60 | 1.91 | 3.44 | 2.20 | 1.58 | 1.99 | 1.99 | 1.86 | 1.53 | 1.85 | 2.12 | 2.93 | 2.30 | 2.43 | 1.86 | 3.06 | 4.17 | 2.64 | 2.50 | 2.56 | 2.27 |
| mz344.707/21.9s | 9 | 3 | 5.19 | 5.20 | 5.09 | 4.30 | 5.64 | 5.38 | 5.42 | 5.05 | 5.88 | 5.62 | 3.12 | 5.24 | 5.24 | 5.65 | 5.93 | 4.99 | 4.63 | 5.37 | 5.20 | 5.24 | 5.33 | 5.41 | 3.47 | 5.46 | 5.34 | 3.36 | 5.31 | 5.11 | 1.66 | 4.89 | 5.84 | 5.84 | 5.67 |
| mz265.192/22.2s | - | 1 | 3.13 | 2.46 | 4.57 | 2.48 | 4.79 | 4.19 | 4.41 | 3.86 | 5.02 | 4.80 | 4.95 | 2.85 | 4.63 | 3.24 | 5.13 | 3.56 | 1.47 | 3.56 | 3.09 | 3.12 | 2.97 | 2.75 | 3.89 | 4.40 | 4.17 | 4.01 | 1.30 | 4.75 | 4.91 | 4.10 | 4.29 | 3.71 | 3.33 |
| mz394.725/22.8s | 12 | 2 | 5.42 | 4.81 | 5.39 | 4.71 | 5.58 | 5.38 | 2.13 | 5.32 | 5.88 | 5.25 | 3.80 | 5.02 | 2.07 | 5.25 | 5.93 | 5.48 | 5.04 | 5.22 | 5.44 | 5.12 | 4.96 | 5.14 | 4.69 | 5.46 | 5.51 | 3.98 | 5.04 | 5.38 | 5.38 | 2.01 | 2.01 | 1.97 | 5.24 |
| mz188.129/23.1s | - | 1 | 2.00 | 1.26 | 4.02 | 2.90 | 4.24 | 3.51 | 4.67 | 3.61 | 4.32 | 3.89 | 4.93 | 3.77 | 3.96 | 3.26 | 3.99 | 3.49 | 2.99 | 3.59 | 3.60 | 3.61 | 3.50 | 3.02 | 4.11 | 4.11 | 4.02 | 4.29 | 3.48 | 3.90 | 3.89 | 4.12 | 3.93 | 4.00 | 4.31 |
| mz302.208/23.6s | 10 | 1 | 4.05 | 3.94 | 2.16 | 4.57 | 4.69 | 5.38 | 2.89 | 4.17 | 3.63 | 3.21 | 2.31 | 4.65 | 3.84 | 4.04 | 3.29 | 4.47 | 3.59 | 4.39 | 4.25 | 4.79 | 4.78 | 5.41 | 2.86 | 3.71 | 4.79 | 3.12 | 3.84 | 3.00 | 2.19 | 3.75 | 3.63 | 3.53 | 2.50 |
| mz132.102/23.9s | - | 1 | 2.21 | 1.38 | 2.66 | 2.20 | 3.04 | 2.84 | 3.66 | 2.75 | 3.16 | 3.01 | 3.70 | 2.98 | 3.26 | 2.85 | 3.31 | 3.04 | 2.32 | 2.61 | 2.65 | 2.43 | 3.20 | 2.59 | 3.15 | 2.80 | 2.62 | 3.76 | 2.55 | 2.77 | 2.79 | 3.25 | 3.07 | 3.07 | 3.03 |
| mz136.062/24.1s | - | 1 | 1.89 | 1.62 | 1.70 | 1.38 | 2.24 | 1.84 | 2.57 | 1.57 | 2.95 | 2.70 | 2.46 | 1.99 | 2.59 | 1.96 | 3.61 | 1.99 | 1.15 | 1.57 | 1.68 | 1.50 | 3.55 | 1.50 | 2.25 | 2.23 | 1.87 | 2.67 | 1.41 | 2.31 | 2.29 | 2.64 | 2.62 | 2.66 | 2.22 |
| mz421.707/24.6s | 8 | 2 | 5.38 | 4.43 | 5.43 | 5.10 | 5.93 | 5.32 | 6.17 | 5.40 | 5.21 | 5.37 | 6.33 | 5.84 | 5.86 | 4.99 | 5.09 | 5.81 | 5.04 | 5.37 | 5.44 | 4.95 | 5.44 | 5.20 | 5.78 | 5.46 | 5.51 | 6.20 | 4.74 | 5.38 | 5.38 | 5.81 | 5.84 | 5.84 | 1.94 |
| mz329.206/24.8s | 9 | 3 | 5.42 | 4.54 | 5.09 | 5.10 | 5.00 | 4.71 | 2.41 | 4.65 | 4.91 | 3.52 | 5.69 | 5.84 | 5.86 | 5.65 | 3.63 | 5.81 | 5.04 | 5.37 | 5.44 | 4.48 | 5.44 | 5.05 | 4.64 | 3.22 | 5.27 | 6.20 | 4.33 | 3.89 | 4.04 | 5.53 | 5.84 | 5.19 | 5.67 |
| mz318.198/25.4s | 8 | 2 | 5.42 | 4.59 | 4.42 | 4.53 | 5.33 | 4.68 | 5.69 | 5.40 | 5.59 | 5.62 | 6.07 | 5.84 | 5.86 | 5.65 | 5.93 | 5.81 | 5.04 | 4.78 | 5.44 | 4.71 | 5.44 | 5.41 | 5.78 | 5.06 | 4.81 | 5.50 | 5.31 | 5.38 | 5.38 | 5.07 | 5.84 | 5.36 | 1.40 |
| mz316.224/25.9s | 10 | 1 | 5.05 | 4.31 | 1.58 | 4.11 | 5.63 | 5.28 | 3.49 | 5.27 | 4.63 | 5.40 | 3.48 | 4.90 | 3.64 | 4.58 | 4.53 | 4.86 | 3.85 | 4.75 | 5.14 | 5.24 | 4.76 | 5.16 | 2.32 | 4.26 | 4.81 | 2.42 | 4.64 | 4.41 | 4.11 | 3.87 | 3.59 | 3.97 | 2.93 |
| **Supplementary Table 2.** Continued. | | | | | | | | | | | | | | | | | | | | | | | | | | | | | | | | | | | |
| **Feature** | **Cluster** | **Charge** | ***Pedobacter* sp. strain** | | | | | | | | | | | | | | | | | | | | | | | | | | | | | | | | |
|  |  |  | **DSM19033** | **DSM19036** | **JCM2171** | **LMG21415** | **LMG29220** | **UP508** | **UP509** | **UP579** | **UP621** | **UP640** | **UP696** | **UP742** | **UP751** | **UP1184** | **UP1189** | **UP1400** | **UP1426** | **UP1427** | **UP1428** | **UP1429** | **UP1430** | **UP1431** | **UP1435** | **UP1436** | **UP1437** | **UP1439** | **UP1440** | **UP1478** | **UP1479** | **UP1634** | **UP1637** | **UP1642** | **UP1729** |
| mz222.113/27.9s | - | 1 | 1.84 | 1.50 | 3.11 | 2.55 | 3.33 | 3.06 | 3.59 | 3.41 | 3.90 | 3.54 | 3.80 | 2.99 | 3.63 | 2.69 | 4.18 | 3.21 | 2.79 | 2.53 | 2.73 | 2.52 | 3.47 | 2.36 | 2.89 | 3.21 | 3.05 | 3.21 | 3.00 | 3.25 | 3.11 | 3.72 | 3.72 | 3.82 | 3.62 |
| mz429.592/27.9s | 9 | 3 | 5.42 | 5.18 | 5.43 | 5.10 | 5.93 | 5.38 | 6.17 | 5.40 | 5.88 | 5.60 | 2.21 | 5.29 | 5.86 | 5.65 | 5.46 | 5.81 | 5.04 | 5.37 | 5.44 | 5.24 | 5.44 | 5.41 | 4.45 | 5.46 | 5.51 | 3.80 | 5.24 | 5.25 | 4.81 | 5.81 | 5.84 | 5.84 | 5.67 |
| mz166.087/28.1s | - | 1 | 2.75 | 1.28 | 3.59 | 2.84 | 3.97 | 4.00 | 4.47 | 4.17 | 5.04 | 4.26 | 4.93 | 3.95 | 4.27 | 3.86 | 4.96 | 3.81 | 3.53 | 3.57 | 3.67 | 3.58 | 4.07 | 3.38 | 3.66 | 4.89 | 3.67 | 3.99 | 3.67 | 3.91 | 3.84 | 4.77 | 4.19 | 4.05 | 4.25 |
| mz468.618/28.2s | 9 | 3 | 5.42 | 5.20 | 5.43 | 5.10 | 5.93 | 5.38 | 5.52 | 5.40 | 5.88 | 5.62 | 2.18 | 5.26 | 5.79 | 5.65 | 5.01 | 5.44 | 5.04 | 5.37 | 5.44 | 5.24 | 5.44 | 5.41 | 3.86 | 5.46 | 5.04 | 3.23 | 5.31 | 4.94 | 4.86 | 5.81 | 5.84 | 5.71 | 5.67 |
| mz272.157/29.1s | 12 | 3 | 4.60 | 4.61 | 5.09 | 4.59 | 5.10 | 5.17 | 1.70 | 4.25 | 5.69 | 5.28 | 2.09 | 5.04 | 2.20 | 4.74 | 4.98 | 5.23 | 5.04 | 4.76 | 5.44 | 5.24 | 5.44 | 5.41 | 2.41 | 5.46 | 5.17 | 2.26 | 4.38 | 5.32 | 4.78 | 1.92 | 1.88 | 1.84 | 4.66 |
| mz278.16/29.1s | 12 | 3 | 5.42 | 5.20 | 5.26 | 5.06 | 5.93 | 5.38 | 2.66 | 4.86 | 5.88 | 5.62 | 3.03 | 5.81 | 3.24 | 5.65 | 5.93 | 5.81 | 5.04 | 5.37 | 5.44 | 5.24 | 5.37 | 5.38 | 3.68 | 5.46 | 5.51 | 3.22 | 5.31 | 5.38 | 5.23 | 3.12 | 2.89 | 2.93 | 5.59 |
| mz334.198/29.1s | 12 | 2 | 4.45 | 5.20 | 5.43 | 4.61 | 5.17 | 5.10 | 2.46 | 5.18 | 5.72 | 5.46 | 2.78 | 5.23 | 3.02 | 4.77 | 5.93 | 5.29 | 4.71 | 5.11 | 5.44 | 4.17 | 4.76 | 5.41 | 3.11 | 4.96 | 5.51 | 2.93 | 4.49 | 5.38 | 5.16 | 2.62 | 2.59 | 2.57 | 5.67 |
| mz340.534/29.1s | 9 | 3 | 5.21 | 5.20 | 5.43 | 5.10 | 5.93 | 5.24 | 3.71 | 5.01 | 5.88 | 5.55 | 2.53 | 5.84 | 5.86 | 5.65 | 5.84 | 5.35 | 5.04 | 5.37 | 5.44 | 5.24 | 5.38 | 5.41 | 3.08 | 5.46 | 5.51 | 2.91 | 5.31 | 5.17 | 5.31 | 5.81 | 5.84 | 5.84 | 5.67 |
| mz554.368/30.2s | 13 | 1 | 5.42 | 5.20 | 5.28 | 5.10 | 5.27 | 5.38 | 5.42 | 5.40 | 5.59 | 5.60 | 6.33 | 5.65 | 1.95 | 5.17 | 5.56 | 5.81 | 4.83 | 5.35 | 5.36 | 5.24 | 4.92 | 5.17 | 5.38 | 5.46 | 5.51 | 6.20 | 5.31 | 5.38 | 5.38 | 2.04 | 1.88 | 2.00 | 5.67 |
| mz368.745/31.8s | 6 | 2 | 5.42 | 5.16 | 5.43 | 5.10 | 5.93 | 5.38 | 5.92 | 5.40 | 5.88 | 5.62 | 6.33 | 5.84 | 5.86 | 5.65 | 2.36 | 5.81 | 5.04 | 5.37 | 5.44 | 5.24 | 5.44 | 5.41 | 5.78 | 5.46 | 5.42 | 6.20 | 5.31 | 1.67 | 1.56 | 5.81 | 5.84 | 5.84 | 5.50 |
| mz138.055/32.1s | - | 1 | 1.92 | 1.73 | 1.80 | 1.55 | 2.39 | 2.05 | 2.56 | 1.79 | 2.55 | 2.29 | 2.76 | 2.07 | 2.28 | 2.04 | 2.33 | 2.50 | 1.35 | 1.73 | 1.80 | 1.60 | 2.21 | 1.81 | 2.14 | 2.05 | 2.16 | 2.63 | 1.69 | 2.08 | 2.03 | 2.32 | 2.28 | 2.31 | 2.10 |
| mz333.879/33.6s | 9 | 3 | 5.42 | 5.20 | 5.43 | 5.10 | 5.93 | 5.38 | 2.27 | 3.20 | 4.18 | 4.37 | 6.33 | 5.84 | 5.86 | 5.65 | 3.65 | 5.81 | 5.04 | 5.37 | 5.44 | 5.24 | 5.44 | 5.41 | 5.59 | 3.58 | 5.51 | 6.16 | 5.31 | 3.83 | 3.48 | 5.81 | 5.84 | 5.84 | 5.67 |
| mz273.217/34.3s | - | 1 | 5.18 | 5.20 | 4.84 | 5.10 | 2.08 | 2.96 | 6.04 | 5.21 | 5.41 | 5.19 | 6.33 | 5.10 | 5.75 | 4.82 | 5.41 | 5.69 | 5.04 | 5.37 | 4.66 | 4.91 | 5.09 | 4.57 | 5.48 | 5.41 | 2.32 | 4.96 | 5.31 | 4.94 | 4.73 | 5.57 | 5.39 | 5.16 | 5.01 |
| mz275.198/34.9s | 3 | 1 | 5.42 | 4.92 | 5.05 | 3.58 | 5.59 | 5.02 | 5.78 | 5.40 | 4.15 | 4.58 | 5.63 | 5.07 | 5.74 | 4.67 | 1.23 | 4.60 | 4.79 | 4.93 | 5.23 | 4.73 | 4.49 | 5.20 | 3.87 | 3.74 | 4.82 | 4.60 | 4.64 | 4.08 | 4.22 | 5.76 | 5.84 | 5.77 | 5.67 |
| mz328.198/34.9s | 12 | 2 | 4.96 | 4.10 | 5.43 | 4.14 | 5.50 | 5.15 | 2.30 | 4.62 | 5.01 | 5.13 | 4.39 | 5.84 | 2.12 | 4.74 | 5.44 | 5.18 | 5.04 | 5.09 | 5.32 | 5.13 | 4.67 | 5.41 | 5.03 | 5.28 | 5.09 | 4.34 | 4.88 | 4.91 | 5.38 | 2.08 | 2.12 | 2.08 | 5.16 |
| mz329.675/34.9s | 12 | 2 | 5.42 | 5.20 | 5.11 | 4.68 | 5.82 | 5.36 | 2.43 | 4.46 | 5.88 | 5.62 | 4.32 | 5.84 | 2.12 | 4.93 | 5.65 | 5.45 | 5.01 | 5.37 | 5.29 | 5.10 | 4.78 | 5.20 | 4.84 | 5.46 | 5.19 | 4.64 | 5.03 | 5.38 | 5.38 | 2.10 | 2.12 | 2.08 | 5.67 |
| mz338.681/34.9s | 12 | 2 | 5.42 | 4.33 | 5.27 | 4.40 | 5.93 | 5.38 | 1.75 | 4.90 | 5.88 | 5.62 | 3.92 | 5.84 | 1.59 | 4.61 | 5.66 | 5.81 | 4.83 | 5.37 | 5.34 | 5.24 | 4.50 | 5.39 | 4.04 | 5.46 | 5.51 | 3.83 | 5.31 | 5.38 | 5.26 | 1.57 | 1.68 | 1.56 | 5.67 |
| mz268.158/35s | 12 | 3 | 5.42 | 5.20 | 5.34 | 5.10 | 5.93 | 5.26 | 1.60 | 4.43 | 5.63 | 5.18 | 3.21 | 5.31 | 1.43 | 5.22 | 5.75 | 5.81 | 5.04 | 5.08 | 5.44 | 5.21 | 5.44 | 5.41 | 3.96 | 5.46 | 5.00 | 4.27 | 4.94 | 5.38 | 4.98 | 1.40 | 1.43 | 1.40 | 4.89 |
| mz127.112/35.1s | 12 | 1 | 5.17 | 5.20 | 4.30 | 4.25 | 4.90 | 4.15 | 2.37 | 4.62 | 4.51 | 4.56 | 4.74 | 5.52 | 2.26 | 4.61 | 4.86 | 5.45 | 3.54 | 4.37 | 4.39 | 5.02 | 4.60 | 5.41 | 4.55 | 4.18 | 4.43 | 4.30 | 3.88 | 4.31 | 4.39 | 2.22 | 2.25 | 2.21 | 5.54 |
| mz435.927/35.1s | 9 | 3 | 5.42 | 5.20 | 4.35 | 4.59 | 4.82 | 5.20 | 5.10 | 5.12 | 4.75 | 4.79 | 2.20 | 5.04 | 5.14 | 5.55 | 4.53 | 5.37 | 4.59 | 5.22 | 5.44 | 4.98 | 5.40 | 5.32 | 3.57 | 5.17 | 4.98 | 3.32 | 5.31 | 4.53 | 4.64 | 5.81 | 5.82 | 5.35 | 5.67 |
| mz617.318/35.1s | - | 1 | 4.30 | 4.18 | 5.17 | 3.38 | 5.23 | 5.38 | 5.50 | 4.83 | 3.74 | 3.67 | 5.66 | 4.55 | 5.69 | 4.80 | 0.93 | 4.35 | 4.34 | 4.51 | 4.82 | 5.24 | 4.34 | 4.64 | 5.07 | 3.57 | 5.07 | 5.24 | 4.49 | 4.05 | 3.62 | 5.20 | 5.13 | 4.88 | 5.67 |
| mz503.298/35.2s | - | 1 | 4.62 | 4.84 | 4.97 | 3.82 | 1.96 | 3.31 | 6.17 | 5.40 | 3.89 | 3.97 | 6.33 | 5.84 | 5.75 | 5.65 | 5.93 | 5.81 | 4.65 | 5.37 | 5.18 | 5.24 | 5.04 | 4.79 | 5.69 | 4.06 | 2.23 | 5.74 | 5.31 | 5.01 | 4.89 | 5.36 | 5.74 | 5.56 | 5.33 |
| mz434.264/35.3s | 10 | 3 | 5.30 | 4.90 | 4.88 | 5.10 | 5.93 | 5.22 | 5.21 | 4.73 | 3.44 | 3.75 | 1.82 | 4.45 | 5.24 | 5.65 | 2.57 | 5.10 | 4.49 | 4.74 | 4.95 | 5.24 | 5.11 | 5.41 | 3.32 | 5.37 | 5.14 | 2.74 | 4.83 | 3.30 | 3.16 | 4.62 | 5.62 | 5.84 | 5.67 |
| **Supplementary Table 2.** Continued. | | | | | | | | | | | | | | | | | | | | | | | | | | | | | | | | | | | |
| **Feature** | **Cluster** | **Charge** | ***Pedobacter* sp. strain** | | | | | | | | | | | | | | | | | | | | | | | | | | | | | | | | |
|  |  |  | **DSM19033** | **DSM19036** | **JCM2171** | **LMG21415** | **LMG29220** | **UP508** | **UP509** | **UP579** | **UP621** | **UP640** | **UP696** | **UP742** | **UP751** | **UP1184** | **UP1189** | **UP1400** | **UP1426** | **UP1427** | **UP1428** | **UP1429** | **UP1430** | **UP1431** | **UP1435** | **UP1436** | **UP1437** | **UP1439** | **UP1440** | **UP1478** | **UP1479** | **UP1634** | **UP1637** | **UP1642** | **UP1729** |
| mz473.29/35.3s | 10 | 3 | 5.42 | 3.81 | 4.07 | 3.55 | 4.40 | 4.42 | 4.74 | 5.40 | 3.46 | 3.69 | 1.97 | 3.97 | 4.56 | 4.10 | 2.69 | 4.64 | 3.78 | 4.12 | 4.49 | 4.15 | 3.84 | 4.86 | 3.25 | 4.30 | 4.48 | 2.85 | 4.09 | 3.17 | 3.40 | 4.81 | 4.64 | 4.73 | 4.52 |
| mz316.224/35.4s | 10 | 1 | 5.03 | 4.47 | 1.61 | 4.47 | 5.29 | 4.81 | 3.25 | 3.62 | 3.70 | 3.50 | 2.30 | 4.88 | 4.45 | 5.56 | 3.77 | 4.93 | 4.39 | 5.00 | 5.19 | 4.84 | 5.06 | 5.14 | 2.48 | 4.06 | 4.87 | 2.57 | 4.01 | 3.10 | 2.06 | 4.29 | 3.91 | 3.83 | 1.82 |
| mz406.912/36.3s | 9 | 3 | 5.42 | 5.06 | 5.43 | 5.10 | 5.77 | 5.38 | 5.68 | 5.40 | 4.42 | 3.95 | 2.71 | 5.26 | 5.86 | 5.58 | 4.16 | 5.81 | 5.04 | 5.37 | 5.24 | 5.24 | 5.44 | 5.02 | 2.56 | 3.91 | 5.28 | 2.40 | 4.16 | 4.22 | 4.72 | 5.39 | 5.84 | 5.84 | 5.67 |
| mz413.193/36.3s | 8 | 2 | 4.41 | 5.20 | 4.58 | 3.86 | 5.18 | 4.35 | 6.09 | 4.48 | 5.03 | 5.36 | 5.73 | 4.68 | 5.02 | 4.98 | 4.96 | 5.22 | 3.97 | 4.36 | 5.19 | 4.15 | 4.41 | 4.48 | 5.78 | 5.46 | 5.06 | 5.29 | 4.26 | 4.88 | 5.38 | 5.41 | 5.03 | 4.76 | 1.91 |
| mz344.534/37.1s | 10 | 3 | 5.42 | 5.04 | 5.37 | 5.10 | 5.93 | 5.23 | 6.17 | 5.09 | 2.41 | 2.57 | 4.23 | 5.84 | 5.81 | 5.65 | 3.30 | 5.76 | 5.04 | 5.37 | 5.41 | 5.24 | 5.44 | 5.41 | 5.11 | 4.70 | 5.51 | 4.55 | 5.31 | 2.02 | 1.40 | 5.73 | 5.84 | 5.84 | 5.67 |
| mz138.055/37.7s | - | 1 | 2.29 | 2.04 | 2.17 | 2.11 | 2.85 | 2.43 | 2.94 | 2.10 | 2.88 | 2.63 | 3.16 | 2.73 | 2.64 | 1.83 | 2.73 | 2.63 | 1.75 | 2.11 | 2.13 | 1.92 | 2.55 | 2.13 | 2.49 | 2.39 | 2.50 | 2.97 | 2.06 | 2.45 | 2.44 | 2.64 | 2.69 | 2.67 | 2.48 |
| mz351.715/38.9s | 9 | 2 | 5.42 | 4.64 | 5.43 | 4.97 | 5.73 | 5.02 | 4.26 | 4.96 | 5.41 | 5.24 | 2.50 | 5.16 | 5.45 | 4.81 | 5.46 | 5.81 | 4.95 | 5.37 | 5.41 | 5.24 | 5.44 | 5.16 | 2.88 | 5.36 | 5.51 | 2.62 | 4.57 | 5.32 | 4.74 | 5.34 | 5.23 | 5.08 | 5.15 |
| mz285.218/39.7s | - | 1 | 5.42 | 5.20 | 4.99 | 4.90 | 1.98 | 2.82 | 6.17 | 5.40 | 5.35 | 5.45 | 6.33 | 5.25 | 5.86 | 5.03 | 5.38 | 5.53 | 5.04 | 5.37 | 5.28 | 5.24 | 5.28 | 5.41 | 5.04 | 5.46 | 2.28 | 5.62 | 4.73 | 5.38 | 5.38 | 5.34 | 5.84 | 5.84 | 5.67 |
| mz469.783/40.9s | 5 | 2 | 5.42 | 5.20 | 4.90 | 2.45 | 2.41 | 2.09 | 6.17 | 5.40 | 1.81 | 1.94 | 5.69 | 5.68 | 5.86 | 5.65 | 5.93 | 5.81 | 5.04 | 5.37 | 5.44 | 5.24 | 5.39 | 5.41 | 5.78 | 2.02 | 3.74 | 6.20 | 5.31 | 5.38 | 5.38 | 5.81 | 5.79 | 5.84 | 5.67 |
| mz267.497/43.6s | 9 | 3 | 5.30 | 5.07 | 5.03 | 5.02 | 5.93 | 5.38 | 6.17 | 5.40 | 5.88 | 5.62 | 2.63 | 5.84 | 5.86 | 5.58 | 5.93 | 5.81 | 5.04 | 5.37 | 5.44 | 5.20 | 5.44 | 5.41 | 2.94 | 5.46 | 5.51 | 2.67 | 5.31 | 5.38 | 5.38 | 5.81 | 5.84 | 5.84 | 5.67 |
| mz239.144/43.7s | 9 | 2 | 4.67 | 5.20 | 4.92 | 4.45 | 4.75 | 4.49 | 6.17 | 4.30 | 4.97 | 4.80 | 2.61 | 5.84 | 4.88 | 5.65 | 5.04 | 4.63 | 4.28 | 4.50 | 4.80 | 4.22 | 4.43 | 4.42 | 2.83 | 4.92 | 5.51 | 2.64 | 3.85 | 4.59 | 4.39 | 5.01 | 4.75 | 4.64 | 5.67 |
| mz270.825/43.7s | 9 | 3 | 5.42 | 5.20 | 5.43 | 5.10 | 5.93 | 5.38 | 5.63 | 5.40 | 5.88 | 5.62 | 2.45 | 5.84 | 5.86 | 5.65 | 5.93 | 5.81 | 5.04 | 5.37 | 5.44 | 5.24 | 5.44 | 5.41 | 2.63 | 5.46 | 5.51 | 2.52 | 5.31 | 5.38 | 5.38 | 4.91 | 5.39 | 5.42 | 5.67 |
| mz341.206/43.7s | 9 | 2 | 5.42 | 4.54 | 4.83 | 5.00 | 5.93 | 5.38 | 5.34 | 5.40 | 5.88 | 4.87 | 2.32 | 4.82 | 4.93 | 5.15 | 5.93 | 4.90 | 4.44 | 5.08 | 4.89 | 4.96 | 4.99 | 4.99 | 2.48 | 4.84 | 5.51 | 2.38 | 4.85 | 5.38 | 5.35 | 5.72 | 5.12 | 5.10 | 5.67 |
| mz423.748/43.7s | 9 | 2 | 5.42 | 5.20 | 5.43 | 5.10 | 5.93 | 5.38 | 5.18 | 5.40 | 5.88 | 5.62 | 1.52 | 5.55 | 5.76 | 5.46 | 5.88 | 5.51 | 4.69 | 5.37 | 5.44 | 5.24 | 5.44 | 5.41 | 1.76 | 5.46 | 5.51 | 1.59 | 5.26 | 5.38 | 5.38 | 4.61 | 5.03 | 4.66 | 5.67 |
| mz120.081/43.9s | - | 1 | 2.94 | 1.91 | 2.74 | 2.88 | 4.12 | 3.79 | 2.49 | 3.78 | 4.56 | 5.62 | 2.41 | 3.53 | 2.30 | 3.96 | 4.54 | 3.62 | 3.06 | 3.51 | 3.72 | 4.14 | 4.21 | 3.41 | 2.68 | 4.22 | 3.69 | 2.42 | 2.99 | 3.90 | 3.94 | 2.27 | 2.20 | 2.49 | 3.99 |
| mz412.702/44.1s | 8 | 2 | 5.42 | 5.20 | 5.41 | 5.06 | 5.93 | 5.38 | 5.75 | 5.40 | 5.86 | 5.62 | 5.96 | 5.76 | 5.86 | 5.56 | 5.38 | 5.81 | 4.83 | 5.25 | 4.85 | 5.24 | 5.44 | 4.96 | 5.78 | 5.46 | 5.51 | 5.95 | 5.21 | 5.38 | 5.38 | 5.81 | 5.84 | 5.84 | 1.68 |
| mz375.754/44.8s | 6 | 2 | 5.42 | 5.09 | 5.43 | 5.10 | 5.93 | 5.38 | 6.17 | 5.40 | 5.88 | 5.62 | 6.33 | 5.49 | 5.80 | 5.65 | 1.70 | 5.81 | 4.97 | 5.37 | 5.44 | 5.24 | 5.44 | 5.41 | 5.58 | 5.46 | 5.51 | 6.20 | 5.31 | 1.34 | 1.19 | 5.81 | 5.84 | 5.84 | 5.67 |
| mz439.934/44.8s | 9 | 3 | 5.42 | 5.20 | 5.43 | 5.10 | 5.93 | 5.38 | 4.81 | 5.40 | 5.47 | 5.01 | 3.09 | 5.50 | 5.84 | 5.65 | 4.03 | 5.81 | 5.04 | 5.37 | 5.44 | 5.07 | 5.44 | 5.41 | 2.68 | 4.84 | 5.41 | 2.49 | 4.75 | 3.96 | 4.48 | 5.50 | 5.84 | 5.84 | 5.00 |
| mz123.117/45.5s | 12 | 1 | 4.54 | 4.82 | 4.42 | 4.70 | 4.68 | 3.95 | 2.20 | 4.05 | 4.57 | 4.36 | 3.92 | 4.54 | 2.11 | 4.45 | 5.03 | 4.64 | 3.90 | 5.37 | 4.22 | 4.23 | 4.57 | 4.68 | 4.29 | 4.30 | 4.29 | 4.24 | 4.86 | 4.27 | 4.02 | 2.08 | 2.08 | 2.08 | 5.67 |
| mz329.675/45.5s | 12 | 2 | 4.89 | 5.05 | 5.43 | 5.10 | 5.93 | 5.38 | 2.28 | 4.89 | 5.68 | 5.62 | 3.47 | 5.82 | 2.18 | 5.65 | 5.93 | 5.29 | 5.04 | 5.37 | 4.84 | 5.13 | 5.44 | 5.10 | 3.72 | 5.33 | 5.51 | 3.57 | 5.31 | 5.38 | 5.38 | 2.15 | 2.21 | 2.18 | 5.67 |
| mz338.681/45.6s | 12 | 2 | 5.42 | 5.20 | 5.43 | 4.98 | 5.68 | 5.38 | 1.71 | 4.77 | 5.72 | 5.62 | 2.73 | 5.78 | 1.63 | 5.65 | 5.55 | 5.54 | 4.59 | 5.13 | 5.44 | 5.24 | 5.44 | 5.41 | 3.59 | 5.46 | 5.51 | 3.61 | 4.66 | 5.38 | 5.38 | 1.31 | 1.61 | 1.61 | 5.17 |
| mz272.829/45.7s | 12 | 3 | 5.42 | 5.20 | 5.40 | 5.10 | 5.86 | 5.38 | 3.16 | 5.40 | 5.88 | 5.62 | 5.17 | 5.84 | 3.10 | 5.65 | 5.93 | 5.77 | 5.04 | 4.99 | 5.44 | 5.24 | 5.44 | 4.66 | 4.90 | 5.46 | 5.51 | 4.78 | 5.31 | 5.38 | 5.38 | 3.12 | 3.05 | 3.15 | 5.67 |
| mz335.206/45.7s | 12 | 2 | 5.33 | 5.09 | 5.39 | 5.10 | 5.82 | 5.29 | 2.73 | 5.40 | 5.88 | 5.62 | 4.21 | 5.84 | 2.65 | 5.65 | 5.93 | 5.24 | 5.04 | 5.37 | 5.44 | 5.24 | 4.96 | 5.41 | 4.49 | 5.46 | 5.51 | 4.56 | 5.28 | 5.29 | 5.38 | 2.65 | 2.66 | 2.66 | 5.67 |
| **Supplementary Table 2.** Continued. | | | | | | | | | | | | | | | | | | | | | | | | | | | | | | | | | | | |
| **Feature** | **Cluster** | **Charge** | ***Pedobacter* sp. strain** | | | | | | | | | | | | | | | | | | | | | | | | | | | | | | | | |
|  |  |  | **DSM19033** | **DSM19036** | **JCM2171** | **LMG21415** | **LMG29220** | **UP508** | **UP509** | **UP579** | **UP621** | **UP640** | **UP696** | **UP742** | **UP751** | **UP1184** | **UP1189** | **UP1400** | **UP1426** | **UP1427** | **UP1428** | **UP1429** | **UP1430** | **UP1431** | **UP1435** | **UP1436** | **UP1437** | **UP1439** | **UP1440** | **UP1478** | **UP1479** | **UP1634** | **UP1637** | **UP1642** | **UP1729** |
| mz502.289/45.9s | 3 | 1 | 5.42 | 4.95 | 4.91 | 5.10 | 5.71 | 5.38 | 5.74 | 5.31 | 5.12 | 3.89 | 5.75 | 4.76 | 5.86 | 5.37 | 1.52 | 5.13 | 4.63 | 4.89 | 5.44 | 5.24 | 5.44 | 5.21 | 5.78 | 3.90 | 5.51 | 6.20 | 5.16 | 3.95 | 3.70 | 5.22 | 5.78 | 5.84 | 5.67 |
| mz355.219/46.5s | 9 | 4 | 4.79 | 5.08 | 5.43 | 5.10 | 5.93 | 5.38 | 4.95 | 5.40 | 4.72 | 4.15 | 3.12 | 5.57 | 5.86 | 5.65 | 5.23 | 5.81 | 5.04 | 5.37 | 5.44 | 5.24 | 5.44 | 5.41 | 3.28 | 4.71 | 5.51 | 2.90 | 4.86 | 4.59 | 4.71 | 5.81 | 5.84 | 5.84 | 5.67 |
| mz568.385/47.1s | 13 | 1 | 5.42 | 4.77 | 5.43 | 5.01 | 5.93 | 5.38 | 6.17 | 5.40 | 5.88 | 5.62 | 6.33 | 5.84 | 1.16 | 5.16 | 4.75 | 5.53 | 5.04 | 5.37 | 5.44 | 5.24 | 5.44 | 5.41 | 5.78 | 5.46 | 5.51 | 6.20 | 5.12 | 5.38 | 5.38 | 1.28 | 1.21 | 1.28 | 5.48 |
| mz446.251/47.2s | 9 | 2 | 5.42 | 4.38 | 3.36 | 4.86 | 5.93 | 5.07 | 4.52 | 5.40 | 5.01 | 5.62 | 2.71 | 5.55 | 5.59 | 5.65 | 4.97 | 5.81 | 4.44 | 5.31 | 5.44 | 5.22 | 4.52 | 4.94 | 2.56 | 5.46 | 5.51 | 2.46 | 5.00 | 5.33 | 5.38 | 5.16 | 5.05 | 5.41 | 5.54 |
| mz120.044/47.6s | - | 1 | 1.71 | 1.50 | 1.85 | 1.70 | 2.51 | 1.86 | 2.43 | 1.59 | 2.37 | 2.06 | 2.59 | 2.13 | 2.11 | 1.85 | 2.39 | 2.26 | 1.31 | 1.57 | 1.65 | 1.43 | 1.89 | 1.64 | 1.97 | 1.93 | 1.94 | 2.51 | 1.64 | 1.85 | 1.83 | 2.10 | 2.17 | 2.23 | 2.01 |
| mz462.943/48.3s | 9 | 3 | 5.42 | 5.20 | 5.43 | 5.10 | 5.93 | 5.38 | 5.28 | 5.40 | 5.05 | 5.14 | 2.50 | 5.06 | 5.86 | 5.65 | 5.86 | 5.81 | 4.63 | 5.37 | 5.44 | 5.24 | 5.41 | 5.41 | 4.03 | 5.37 | 5.51 | 4.19 | 5.31 | 4.68 | 4.97 | 5.81 | 5.84 | 5.84 | 5.67 |
| mz473.278/48.6s | - | 3 | 4.67 | 5.20 | 1.65 | 4.45 | 5.93 | 5.23 | 5.59 | 5.25 | 5.88 | 5.62 | 6.33 | 5.48 | 5.86 | 4.59 | 5.23 | 5.81 | 5.04 | 5.37 | 5.44 | 5.24 | 5.44 | 5.41 | 5.78 | 5.46 | 5.51 | 6.20 | 5.31 | 5.38 | 4.52 | 5.81 | 5.84 | 5.27 | 5.67 |
| mz261.668/49.9s | 3 | 2 | 5.42 | 5.20 | 5.43 | 5.10 | 5.85 | 5.38 | 6.17 | 5.18 | 4.36 | 4.34 | 6.33 | 5.84 | 5.86 | 5.65 | 2.04 | 5.81 | 5.04 | 5.29 | 5.44 | 5.24 | 5.44 | 5.38 | 4.93 | 4.55 | 5.51 | 5.29 | 5.31 | 4.28 | 5.38 | 5.81 | 5.84 | 5.84 | 5.67 |
| mz476.791/50s | 5 | 2 | 5.05 | 5.20 | 4.58 | 1.85 | 1.64 | 1.82 | 6.17 | 5.40 | 1.67 | 1.84 | 5.87 | 5.77 | 5.86 | 5.43 | 5.93 | 5.76 | 5.04 | 5.37 | 5.44 | 5.24 | 5.44 | 5.41 | 5.78 | 2.12 | 1.94 | 6.17 | 4.89 | 5.38 | 5.38 | 5.81 | 5.70 | 5.68 | 5.61 |
| mz121.117/51s | - | 2 | 5.42 | 5.20 | 5.43 | 5.10 | 2.93 | 3.81 | 6.17 | 5.40 | 5.88 | 5.62 | 6.33 | 5.84 | 5.86 | 5.65 | 5.93 | 5.81 | 5.04 | 5.37 | 5.44 | 5.24 | 5.44 | 5.41 | 5.78 | 5.46 | 3.37 | 6.20 | 5.31 | 5.38 | 5.38 | 5.81 | 5.84 | 5.84 | 5.67 |
| mz287.233/51.1s | - | 1 | 5.31 | 5.20 | 5.43 | 4.82 | 1.28 | 1.95 | 6.17 | 5.21 | 5.88 | 5.62 | 6.19 | 5.69 | 5.69 | 5.65 | 5.93 | 5.81 | 5.04 | 5.37 | 5.44 | 5.24 | 5.30 | 5.41 | 5.73 | 4.96 | 1.63 | 6.15 | 5.31 | 5.38 | 5.38 | 5.81 | 5.84 | 5.82 | 5.65 |
| mz443.232/52.1s | 8 | 3 | 4.68 | 4.94 | 5.26 | 4.12 | 5.36 | 4.84 | 5.39 | 5.40 | 5.35 | 4.85 | 5.49 | 5.84 | 5.64 | 4.72 | 5.49 | 5.18 | 4.34 | 4.78 | 5.05 | 5.21 | 4.54 | 5.41 | 5.64 | 5.41 | 5.51 | 5.75 | 4.36 | 4.60 | 5.21 | 5.09 | 5.58 | 5.42 | 1.87 |
| mz482.259/52.1s | 8 | 3 | 5.42 | 5.20 | 5.43 | 4.44 | 5.70 | 5.38 | 6.17 | 5.40 | 5.88 | 5.62 | 6.09 | 5.84 | 5.37 | 4.99 | 5.93 | 5.43 | 4.64 | 5.37 | 5.28 | 5.24 | 4.93 | 5.41 | 5.78 | 5.46 | 5.51 | 6.05 | 5.31 | 5.38 | 4.04 | 5.00 | 5.67 | 5.74 | 1.66 |
| mz216.161/52.6s | 9 | 1 | 0.55 | 3.94 | 5.43 | 5.10 | 5.93 | 4.37 | 4.91 | 4.55 | 5.88 | 4.92 | 4.90 | 4.18 | 5.86 | 5.65 | 5.27 | 5.81 | 5.04 | 4.18 | 5.44 | 4.71 | 5.44 | 4.62 | 4.47 | 5.46 | 4.47 | 6.20 | 5.31 | 5.38 | 4.08 | 4.97 | 4.12 | 4.45 | 4.40 |
| mz333.707/52.6s | - | 4 | 5.12 | 5.20 | 5.43 | 5.10 | 5.93 | 5.38 | 5.40 | 5.40 | 4.83 | 4.49 | 3.09 | 5.66 | 5.86 | 5.65 | 4.83 | 5.17 | 5.04 | 5.37 | 5.44 | 5.24 | 5.44 | 5.41 | 2.18 | 5.05 | 5.51 | 2.07 | 4.37 | 5.21 | 4.93 | 5.81 | 5.84 | 5.84 | 5.29 |
| mz378.229/53.2s | 10 | 3 | 5.08 | 5.20 | 4.73 | 5.10 | 5.36 | 5.38 | 5.12 | 5.05 | 3.12 | 2.82 | 3.06 | 5.20 | 5.05 | 5.30 | 2.81 | 5.21 | 4.68 | 5.02 | 5.44 | 5.24 | 5.00 | 5.41 | 2.28 | 3.11 | 5.14 | 2.23 | 4.34 | 3.09 | 2.98 | 5.19 | 5.84 | 5.22 | 5.12 |
| mz441.943/53.3s | 7 | 3 | 5.42 | 5.20 | 5.43 | 5.10 | 5.93 | 5.38 | 2.02 | 4.01 | 5.88 | 5.03 | 6.16 | 5.84 | 5.86 | 5.65 | 5.93 | 5.72 | 5.04 | 5.22 | 5.44 | 5.24 | 5.44 | 5.41 | 5.78 | 5.46 | 5.51 | 6.20 | 5.31 | 5.30 | 5.38 | 5.81 | 5.64 | 5.45 | 5.67 |
| mz120.063/53.7s | - | 1 | 5.42 | 5.20 | 5.43 | 5.10 | 5.93 | 5.38 | 6.17 | 5.40 | 5.88 | 2.16 | 2.50 | 5.84 | 5.86 | 5.65 | 5.93 | 5.81 | 5.04 | 5.37 | 5.44 | 5.24 | 5.44 | 5.41 | 5.78 | 5.46 | 5.51 | 6.20 | 5.31 | 5.38 | 5.38 | 5.81 | 5.84 | 5.84 | 5.67 |
| mz557.367/53.7s | 9 | 1 | 5.42 | 5.20 | 2.39 | 5.10 | 5.93 | 5.38 | 3.75 | 4.22 | 4.79 | 4.58 | 3.49 | 5.84 | 5.83 | 5.65 | 4.35 | 5.81 | 5.04 | 5.37 | 5.44 | 5.24 | 5.35 | 5.01 | 2.24 | 4.34 | 5.51 | 2.19 | 5.31 | 4.42 | 5.38 | 5.81 | 5.35 | 5.84 | 5.34 |
| mz275.497/54s | 9 | 3 | 5.42 | 5.20 | 5.43 | 5.10 | 5.93 | 5.38 | 6.17 | 5.40 | 5.88 | 5.62 | 2.62 | 5.84 | 5.86 | 5.65 | 5.93 | 5.81 | 5.04 | 5.37 | 5.44 | 5.24 | 5.44 | 5.41 | 2.90 | 5.46 | 5.51 | 2.69 | 5.31 | 5.38 | 5.38 | 5.81 | 5.84 | 5.84 | 5.67 |
| mz646.394/54.1s | 14 | 1 | 5.13 | 5.20 | 5.43 | 5.06 | 5.20 | 5.38 | 6.17 | 5.37 | 5.88 | 5.62 | 6.33 | 1.95 | 5.86 | 2.10 | 5.93 | 2.12 | 5.04 | 5.37 | 5.44 | 5.24 | 2.15 | 5.41 | 5.62 | 5.46 | 4.56 | 6.20 | 5.19 | 5.38 | 5.38 | 5.81 | 5.84 | 5.84 | 5.62 |
| mz281.501/54.3s | 9 | 3 | 5.42 | 5.20 | 5.43 | 5.10 | 5.93 | 5.38 | 4.91 | 5.40 | 5.88 | 5.62 | 1.52 | 5.04 | 5.86 | 5.49 | 5.69 | 5.81 | 5.04 | 5.37 | 5.44 | 5.24 | 5.22 | 5.41 | 1.77 | 5.34 | 5.51 | 1.63 | 4.19 | 5.38 | 5.38 | 4.97 | 5.72 | 5.03 | 5.67 |
| mz430.756/54.3s | 9 | 2 | 5.42 | 5.20 | 5.43 | 5.10 | 5.93 | 5.38 | 5.03 | 5.40 | 5.40 | 5.62 | 1.38 | 5.56 | 5.43 | 5.39 | 5.34 | 5.81 | 4.86 | 5.37 | 5.44 | 5.24 | 5.44 | 5.41 | 1.69 | 5.46 | 5.51 | 1.46 | 4.20 | 5.38 | 5.38 | 5.22 | 5.39 | 5.80 | 5.67 |
| **Supplementary Table 2.** Continued. | | | | | | | | | | | | | | | | | | | | | | | | | | | | | | | | | | | |
| **Feature** | **Cluster** | **Charge** | ***Pedobacter* sp. strain** | | | | | | | | | | | | | | | | | | | | | | | | | | | | | | | | |
|  |  |  | **DSM19033** | **DSM19036** | **JCM2171** | **LMG21415** | **LMG29220** | **UP508** | **UP509** | **UP579** | **UP621** | **UP640** | **UP696** | **UP742** | **UP751** | **UP1184** | **UP1189** | **UP1400** | **UP1426** | **UP1427** | **UP1428** | **UP1429** | **UP1430** | **UP1431** | **UP1435** | **UP1436** | **UP1437** | **UP1439** | **UP1440** | **UP1478** | **UP1479** | **UP1634** | **UP1637** | **UP1642** | **UP1729** |
| mz393.718/54.4s | 9 | 2 | 5.42 | 5.20 | 5.09 | 4.88 | 5.93 | 5.38 | 5.43 | 5.40 | 5.88 | 5.62 | 2.19 | 5.31 | 5.49 | 5.54 | 5.93 | 5.78 | 5.04 | 5.37 | 5.44 | 5.24 | 5.11 | 5.34 | 2.43 | 5.46 | 5.51 | 2.28 | 5.31 | 5.38 | 5.38 | 5.29 | 5.48 | 5.28 | 4.86 |
| mz267.485/54.5s | 9 | 3 | 5.42 | 5.20 | 5.43 | 5.10 | 5.93 | 5.38 | 5.02 | 5.40 | 5.88 | 5.62 | 2.39 | 5.84 | 4.73 | 5.65 | 5.93 | 5.81 | 5.04 | 5.37 | 5.44 | 5.24 | 5.28 | 5.41 | 2.62 | 5.46 | 5.51 | 2.48 | 5.10 | 5.38 | 5.38 | 5.81 | 4.75 | 5.26 | 5.67 |
| mz348.214/54.5s | 9 | 2 | 5.42 | 4.60 | 5.27 | 5.10 | 5.76 | 5.38 | 5.20 | 5.09 | 5.88 | 5.62 | 2.47 | 5.06 | 5.69 | 4.67 | 5.29 | 5.81 | 5.04 | 5.37 | 4.75 | 5.24 | 5.12 | 5.41 | 2.66 | 5.46 | 5.51 | 2.55 | 5.03 | 5.38 | 5.38 | 5.60 | 5.67 | 4.89 | 5.67 |
| mz467.615/56.3s | 10 | 3 | 5.42 | 5.20 | 5.43 | 5.10 | 5.93 | 5.27 | 5.26 | 5.04 | 2.98 | 3.11 | 2.30 | 5.40 | 5.86 | 5.34 | 3.45 | 5.81 | 5.04 | 5.37 | 5.44 | 5.24 | 5.44 | 5.41 | 3.42 | 4.21 | 5.51 | 3.07 | 5.09 | 2.70 | 3.07 | 5.81 | 5.80 | 5.84 | 5.41 |
| mz453.269/56.6s | 9 | 3 | 5.42 | 5.20 | 5.43 | 5.10 | 5.93 | 5.38 | 4.25 | 5.40 | 5.76 | 5.05 | 2.31 | 5.84 | 5.86 | 5.65 | 5.56 | 5.49 | 5.00 | 5.37 | 5.17 | 5.24 | 5.44 | 5.33 | 2.54 | 4.94 | 5.51 | 2.57 | 5.31 | 5.16 | 5.11 | 5.81 | 5.76 | 5.44 | 5.45 |
| mz678.367/56.6s | 14 | 1 | 5.42 | 5.20 | 5.43 | 5.10 | 5.93 | 5.38 | 6.17 | 5.40 | 5.88 | 5.62 | 6.21 | 1.65 | 5.86 | 1.64 | 5.93 | 1.66 | 5.04 | 5.37 | 5.44 | 5.24 | 1.49 | 5.41 | 5.78 | 5.46 | 5.51 | 6.05 | 5.31 | 5.38 | 5.38 | 5.81 | 5.84 | 5.84 | 5.67 |
| mz503.304/58s | - | 2 | 5.32 | 5.16 | 5.21 | 3.24 | 2.21 | 3.38 | 5.95 | 5.40 | 3.44 | 3.41 | 5.74 | 5.35 | 5.67 | 5.65 | 5.93 | 5.66 | 5.04 | 5.28 | 5.44 | 5.21 | 5.10 | 5.33 | 5.49 | 3.86 | 2.10 | 6.18 | 4.91 | 4.84 | 5.38 | 5.42 | 5.84 | 5.43 | 5.67 |
| mz449.567/59.1s | 8 | 3 | 5.03 | 4.62 | 4.79 | 4.73 | 5.93 | 4.79 | 5.17 | 5.40 | 5.87 | 5.60 | 6.33 | 5.31 | 5.51 | 5.56 | 5.54 | 5.43 | 5.04 | 5.37 | 5.44 | 5.24 | 5.37 | 5.41 | 5.72 | 5.46 | 5.40 | 5.92 | 4.69 | 4.85 | 5.29 | 5.10 | 4.80 | 5.16 | 1.87 |
| mz333.706/59.5s | 9 | 4 | 5.42 | 5.16 | 5.43 | 5.10 | 5.86 | 5.25 | 5.13 | 5.40 | 3.94 | 4.10 | 3.20 | 5.72 | 5.11 | 5.65 | 3.85 | 5.81 | 5.04 | 5.37 | 5.44 | 5.24 | 5.44 | 5.41 | 2.55 | 3.94 | 5.51 | 2.41 | 4.81 | 3.94 | 4.43 | 5.35 | 5.16 | 5.62 | 5.51 |
| mz447.904/59.5s | 8 | 3 | 5.40 | 5.20 | 5.17 | 4.23 | 5.76 | 5.38 | 6.17 | 5.40 | 5.88 | 5.54 | 6.33 | 5.82 | 5.86 | 5.65 | 5.93 | 5.81 | 5.04 | 5.37 | 5.44 | 5.24 | 5.44 | 5.41 | 5.78 | 5.46 | 5.51 | 6.20 | 5.27 | 5.38 | 5.38 | 5.81 | 5.84 | 5.60 | 1.37 |
| mz486.932/59.5s | 8 | 3 | 5.42 | 5.18 | 5.43 | 4.33 | 5.93 | 5.38 | 6.17 | 5.40 | 5.88 | 5.52 | 6.33 | 5.50 | 4.94 | 5.48 | 5.74 | 5.81 | 5.04 | 5.37 | 5.44 | 5.24 | 5.38 | 5.41 | 5.78 | 5.36 | 5.51 | 6.20 | 5.31 | 5.20 | 5.38 | 5.81 | 5.04 | 4.90 | 1.18 |
| mz710.339/59.5s | 14 | 1 | 5.39 | 5.20 | 5.43 | 5.10 | 5.93 | 5.38 | 6.17 | 5.40 | 5.80 | 5.62 | 6.33 | 2.10 | 5.86 | 1.79 | 5.93 | 1.85 | 5.01 | 5.37 | 5.44 | 5.24 | 1.48 | 5.41 | 5.78 | 5.46 | 5.51 | 6.20 | 5.31 | 5.38 | 5.38 | 5.81 | 5.84 | 5.84 | 5.67 |
| mz483.799/59.6s | 5 | 2 | 4.53 | 5.20 | 4.28 | 1.66 | 1.17 | 1.69 | 6.17 | 5.03 | 2.04 | 2.03 | 5.41 | 5.84 | 5.86 | 5.65 | 5.67 | 5.81 | 5.04 | 5.37 | 5.44 | 4.74 | 5.28 | 5.41 | 5.11 | 2.56 | 1.22 | 5.51 | 4.49 | 5.38 | 5.38 | 5.81 | 5.84 | 5.84 | 5.67 |
| mz250.144/60.4s | - | 1 | 1.51 | 2.45 | 3.71 | 3.91 | 3.88 | 4.14 | 4.58 | 3.20 | 4.72 | 4.19 | 4.80 | 3.98 | 4.35 | 3.81 | 3.58 | 4.38 | 2.50 | 4.00 | 4.29 | 3.93 | 2.09 | 3.66 | 4.32 | 4.79 | 3.82 | 4.52 | 2.39 | 4.14 | 4.68 | 5.31 | 4.39 | 4.52 | 4.41 |
| mz415.748/60.9s | 12 | 2 | 5.42 | 5.20 | 5.43 | 5.10 | 5.89 | 5.38 | 3.12 | 5.01 | 5.84 | 5.62 | 2.52 | 5.84 | 2.63 | 5.47 | 5.83 | 5.62 | 4.89 | 5.23 | 5.44 | 4.95 | 5.44 | 5.41 | 2.72 | 5.32 | 5.51 | 2.46 | 4.71 | 5.23 | 5.38 | 2.61 | 2.70 | 2.75 | 5.67 |
| mz446.614/62.7s | 7 | 3 | 5.42 | 5.20 | 5.43 | 5.10 | 5.89 | 5.38 | 2.55 | 4.35 | 5.88 | 5.62 | 6.33 | 5.84 | 5.86 | 5.65 | 5.93 | 5.71 | 5.04 | 5.37 | 5.44 | 5.24 | 5.44 | 5.41 | 5.60 | 5.46 | 5.00 | 6.20 | 5.31 | 5.38 | 5.38 | 5.81 | 5.84 | 5.84 | 5.67 |
| mz268.676/63.7s | 3 | 2 | 5.42 | 5.13 | 5.43 | 4.53 | 5.26 | 5.38 | 6.17 | 5.40 | 4.64 | 5.62 | 6.33 | 5.84 | 5.86 | 5.65 | 2.00 | 5.72 | 5.04 | 5.37 | 5.44 | 5.24 | 5.44 | 5.41 | 5.78 | 4.19 | 5.51 | 6.20 | 5.15 | 4.60 | 4.93 | 5.81 | 5.84 | 5.84 | 5.67 |
| mz582.401/64.5s | 13 | 1 | 5.36 | 5.20 | 5.43 | 5.10 | 5.83 | 5.38 | 6.17 | 5.40 | 5.88 | 5.62 | 6.33 | 5.84 | 1.01 | 5.35 | 5.93 | 5.81 | 5.04 | 5.22 | 5.44 | 5.24 | 5.44 | 5.41 | 5.78 | 5.46 | 5.51 | 6.20 | 5.31 | 5.38 | 5.38 | 1.19 | 1.11 | 1.15 | 5.17 |
| mz457.943/66s | 9 | 3 | 5.42 | 5.20 | 5.43 | 5.10 | 5.93 | 5.38 | 6.17 | 5.32 | 4.19 | 3.62 | 2.36 | 5.49 | 5.45 | 5.65 | 5.07 | 5.81 | 5.04 | 5.37 | 5.44 | 5.23 | 5.44 | 5.41 | 3.00 | 3.48 | 5.51 | 2.67 | 5.31 | 4.16 | 4.73 | 5.81 | 5.76 | 5.78 | 5.67 |
| mz413.263/70s | 11 | 2 | 4.20 | 3.89 | 4.08 | 4.24 | 4.70 | 4.24 | 4.94 | 4.26 | 4.58 | 4.37 | 5.45 | 4.84 | 4.57 | 2.16 | 4.91 | 2.08 | 3.87 | 4.19 | 4.10 | 4.02 | 2.28 | 4.05 | 4.74 | 4.24 | 4.22 | 5.02 | 4.16 | 3.97 | 4.14 | 4.66 | 4.73 | 4.67 | 4.32 |
| mz418.595/70.1s | 7 | 3 | 5.42 | 5.20 | 5.43 | 4.88 | 5.93 | 5.38 | 1.77 | 3.56 | 5.88 | 5.62 | 6.28 | 5.84 | 5.86 | 5.65 | 5.93 | 5.63 | 5.04 | 5.37 | 5.44 | 5.24 | 5.25 | 5.41 | 5.78 | 5.46 | 5.43 | 6.20 | 5.31 | 5.38 | 5.38 | 5.81 | 5.84 | 5.84 | 5.67 |
| mz451.289/70.1s | 7 | 3 | 4.90 | 5.20 | 5.43 | 4.93 | 5.48 | 5.38 | 1.09 | 4.73 | 5.88 | 5.62 | 5.95 | 5.68 | 5.86 | 5.57 | 5.93 | 5.81 | 5.04 | 5.37 | 5.44 | 5.24 | 4.85 | 5.41 | 5.28 | 5.46 | 5.51 | 5.48 | 5.05 | 5.38 | 5.38 | 5.81 | 5.84 | 5.84 | 5.67 |
| mz660.41/70.3s | 14 | 1 | 5.42 | 5.20 | 5.43 | 5.10 | 5.75 | 5.38 | 6.17 | 5.40 | 5.88 | 5.62 | 6.33 | 1.48 | 5.86 | 1.62 | 5.93 | 1.55 | 5.04 | 5.34 | 5.44 | 5.14 | 1.71 | 5.17 | 5.78 | 5.46 | 5.51 | 6.20 | 5.31 | 5.38 | 5.38 | 5.81 | 5.84 | 5.84 | 5.67 |
| **Supplementary Table 2.** Continued. | | | | | | | | | | | | | | | | | | | | | | | | | | | | | | | | | | | |
| **Feature** | **Cluster** | **Charge** | ***Pedobacter* sp. strain** | | | | | | | | | | | | | | | | | | | | | | | | | | | | | | | | |
|  |  |  | **DSM19033** | **DSM19036** | **JCM2171** | **LMG21415** | **LMG29220** | **UP508** | **UP509** | **UP579** | **UP621** | **UP640** | **UP696** | **UP742** | **UP751** | **UP1184** | **UP1189** | **UP1400** | **UP1426** | **UP1427** | **UP1428** | **UP1429** | **UP1430** | **UP1431** | **UP1435** | **UP1436** | **UP1437** | **UP1439** | **UP1440** | **UP1478** | **UP1479** | **UP1634** | **UP1637** | **UP1642** | **UP1729** |
| mz467.287/71.4s | 10 | 3 | 5.42 | 5.01 | 5.43 | 4.86 | 5.93 | 5.38 | 6.17 | 5.21 | 2.32 | 2.68 | 3.17 | 4.56 | 5.86 | 5.36 | 1.90 | 5.50 | 4.64 | 4.73 | 5.44 | 5.24 | 5.44 | 4.85 | 3.24 | 3.09 | 5.07 | 2.73 | 5.31 | 4.07 | 4.34 | 5.46 | 5.84 | 5.84 | 5.61 |
| mz132.102/71.5s | - | 1 | 2.50 | 2.13 | 2.48 | 2.00 | 2.81 | 2.46 | 3.37 | 2.53 | 2.35 | 2.63 | 3.71 | 2.01 | 3.13 | 2.30 | 2.96 | 2.06 | 2.13 | 2.50 | 2.32 | 2.36 | 2.77 | 2.53 | 2.94 | 2.30 | 2.47 | 3.34 | 2.27 | 2.56 | 2.48 | 3.01 | 2.98 | 2.94 | 2.62 |
| mz282.173/71.6s | 9 | 3 | 4.94 | 5.15 | 5.06 | 5.10 | 5.93 | 5.03 | 4.25 | 4.88 | 5.88 | 5.07 | 2.32 | 5.84 | 4.16 | 5.65 | 5.93 | 5.53 | 5.04 | 5.37 | 4.88 | 5.24 | 5.44 | 5.27 | 2.42 | 4.99 | 5.51 | 2.24 | 4.33 | 5.38 | 5.38 | 3.97 | 4.31 | 4.00 | 5.67 |
| mz451.286/72.1s | 7 | 3 | 5.42 | 5.20 | 5.43 | 5.10 | 5.47 | 5.38 | 2.11 | 2.60 | 5.76 | 5.62 | 5.20 | 5.20 | 5.73 | 5.65 | 5.93 | 5.81 | 5.04 | 5.37 | 5.44 | 5.24 | 5.20 | 5.41 | 5.78 | 5.23 | 5.51 | 5.04 | 5.31 | 5.38 | 5.38 | 5.81 | 5.84 | 5.84 | 5.67 |
| mz699.421/72.5s | 5 | 1 | 5.42 | 5.20 | 5.43 | 2.59 | 2.02 | 2.83 | 6.17 | 5.17 | 2.26 | 2.32 | 5.56 | 5.26 | 5.86 | 5.65 | 5.93 | 5.43 | 5.04 | 5.37 | 5.44 | 5.24 | 5.33 | 5.41 | 5.78 | 3.05 | 2.29 | 5.97 | 5.31 | 5.34 | 5.38 | 5.81 | 5.84 | 5.84 | 5.67 |
| mz429.924/73.2s | 9 | 3 | 5.42 | 5.09 | 5.43 | 5.10 | 5.93 | 5.38 | 5.38 | 5.40 | 5.07 | 5.13 | 2.01 | 5.84 | 5.86 | 5.65 | 5.84 | 5.81 | 5.04 | 5.22 | 5.44 | 5.24 | 5.44 | 5.28 | 2.88 | 5.15 | 5.51 | 2.08 | 4.70 | 5.38 | 5.38 | 5.78 | 5.42 | 5.84 | 5.44 |
| mz692.382/73.4s | 14 | 1 | 5.42 | 5.14 | 5.26 | 5.10 | 5.93 | 5.38 | 6.17 | 5.40 | 5.88 | 5.62 | 6.33 | 1.48 | 5.86 | 1.47 | 5.93 | 1.39 | 5.04 | 5.37 | 5.44 | 5.24 | 1.47 | 5.41 | 5.78 | 5.46 | 5.51 | 6.20 | 5.07 | 5.38 | 5.38 | 5.74 | 5.44 | 5.84 | 5.67 |
| mz462.617/73.9s | 9 | 3 | 5.32 | 5.20 | 5.19 | 5.10 | 5.42 | 5.38 | 2.37 | 4.72 | 3.91 | 3.81 | 1.25 | 4.80 | 5.86 | 5.65 | 4.76 | 5.50 | 5.04 | 5.37 | 5.44 | 5.24 | 5.44 | 5.41 | 1.34 | 3.68 | 5.51 | 1.28 | 4.23 | 3.62 | 4.86 | 5.59 | 5.84 | 5.84 | 5.67 |
| mz150.059/74s | 11 | 1 | 4.72 | 4.35 | 4.69 | 4.13 | 3.39 | 4.21 | 6.09 | 4.90 | 4.35 | 4.88 | 6.33 | 2.16 | 5.25 | 2.08 | 5.36 | 2.06 | 4.96 | 5.37 | 5.44 | 4.38 | 2.38 | 4.56 | 4.98 | 5.07 | 3.88 | 6.10 | 4.72 | 5.14 | 5.38 | 5.57 | 5.15 | 5.61 | 5.54 |
| mz724.354/74.3s | 14 | 1 | 5.42 | 5.20 | 5.43 | 5.10 | 5.93 | 5.38 | 6.17 | 5.40 | 5.88 | 5.62 | 6.33 | 2.11 | 5.86 | 2.15 | 5.93 | 1.98 | 5.04 | 5.37 | 5.44 | 5.24 | 2.08 | 5.41 | 5.78 | 5.46 | 5.51 | 6.20 | 5.31 | 5.38 | 5.38 | 5.81 | 5.84 | 5.84 | 5.64 |
| mz389.769/74.8s | 6 | 2 | 5.42 | 5.20 | 5.43 | 5.01 | 5.93 | 5.38 | 5.99 | 5.40 | 5.88 | 5.62 | 6.33 | 5.84 | 5.86 | 5.52 | 1.79 | 5.27 | 5.04 | 5.37 | 5.44 | 5.24 | 5.44 | 5.41 | 5.78 | 5.46 | 5.51 | 6.20 | 5.31 | 1.94 | 1.77 | 5.81 | 5.84 | 5.84 | 5.67 |
| mz463.273/74.9s | 11 | 3 | 5.42 | 5.05 | 4.73 | 4.63 | 5.71 | 5.38 | 2.91 | 5.40 | 5.23 | 5.10 | 1.75 | 1.23 | 5.86 | 1.53 | 5.47 | 1.46 | 5.04 | 5.37 | 5.44 | 5.24 | 1.87 | 5.41 | 1.87 | 5.46 | 5.51 | 1.83 | 4.78 | 4.40 | 4.66 | 5.50 | 5.71 | 5.60 | 5.48 |
| mz285.181/75.6s | 13 | 1 | 5.42 | 4.64 | 4.45 | 4.10 | 5.62 | 5.38 | 5.90 | 5.40 | 5.88 | 4.94 | 6.06 | 5.09 | 2.16 | 5.65 | 5.93 | 5.81 | 5.04 | 4.82 | 4.38 | 4.26 | 5.44 | 4.14 | 5.78 | 5.46 | 5.12 | 5.82 | 4.65 | 5.38 | 5.38 | 2.68 | 2.63 | 2.68 | 5.56 |
| mz451.619/76.4s | 7 | 3 | 5.42 | 5.20 | 5.43 | 5.10 | 5.93 | 5.38 | 2.36 | 4.00 | 5.56 | 5.62 | 6.33 | 5.84 | 5.86 | 5.65 | 5.90 | 5.81 | 5.04 | 5.37 | 5.44 | 5.24 | 5.44 | 5.41 | 5.78 | 5.46 | 5.51 | 6.20 | 5.31 | 5.38 | 5.38 | 5.81 | 5.84 | 5.84 | 5.67 |
| mz467.287/76.8s | 10 | 3 | 5.23 | 5.09 | 4.78 | 4.34 | 5.93 | 4.78 | 2.88 | 4.38 | 1.79 | 1.60 | 2.93 | 4.49 | 4.85 | 4.69 | 2.49 | 4.86 | 4.31 | 4.57 | 5.11 | 5.24 | 4.97 | 5.41 | 2.07 | 1.57 | 4.71 | 2.71 | 3.43 | 1.81 | 3.08 | 5.00 | 5.43 | 5.15 | 5.51 |
| mz596.415/77s | 13 | 1 | 5.42 | 5.20 | 5.43 | 5.10 | 5.93 | 5.38 | 6.17 | 5.40 | 5.88 | 5.62 | 6.33 | 5.84 | 2.05 | 5.65 | 5.93 | 5.81 | 5.04 | 5.37 | 5.44 | 5.24 | 5.44 | 5.38 | 5.78 | 5.40 | 5.51 | 6.20 | 5.31 | 5.38 | 5.38 | 2.16 | 2.11 | 2.17 | 5.67 |
| mz462.614/77.2s | 9 | 3 | 5.42 | 5.20 | 5.43 | 5.10 | 5.93 | 5.38 | 6.17 | 5.40 | 5.88 | 5.62 | 2.04 | 5.84 | 5.86 | 5.65 | 5.93 | 5.81 | 5.04 | 5.37 | 5.44 | 5.24 | 5.44 | 5.41 | 3.69 | 5.46 | 5.51 | 3.33 | 5.31 | 5.38 | 5.38 | 5.81 | 5.84 | 5.84 | 5.67 |
| mz455.961/77.6s | 7 | 3 | 5.42 | 5.08 | 5.43 | 5.10 | 5.71 | 5.38 | 2.88 | 5.31 | 5.33 | 5.62 | 5.81 | 5.84 | 5.86 | 5.21 | 5.93 | 5.35 | 5.04 | 5.37 | 5.44 | 5.24 | 4.57 | 5.41 | 5.78 | 5.46 | 5.51 | 6.05 | 5.31 | 5.00 | 5.32 | 5.81 | 5.76 | 5.84 | 5.67 |
| mz481.256/77.6s | 8 | 3 | 5.42 | 5.20 | 5.43 | 5.07 | 5.93 | 5.38 | 5.59 | 5.40 | 5.88 | 5.62 | 5.47 | 5.84 | 4.80 | 5.65 | 5.93 | 5.81 | 5.04 | 5.37 | 5.44 | 5.24 | 5.44 | 5.41 | 5.78 | 5.46 | 5.51 | 6.20 | 5.31 | 5.29 | 5.28 | 4.29 | 4.28 | 4.85 | 1.58 |
| mz418.595/77.8s | 7 | 3 | 5.24 | 5.20 | 5.43 | 5.10 | 5.93 | 5.38 | 1.69 | 3.05 | 5.88 | 5.62 | 6.33 | 5.84 | 5.86 | 5.65 | 5.93 | 5.81 | 5.04 | 5.37 | 5.44 | 5.24 | 5.44 | 5.41 | 5.78 | 5.46 | 5.51 | 6.20 | 5.31 | 5.38 | 5.38 | 5.81 | 5.76 | 5.84 | 5.67 |
| mz471.959/79.4s | 3 | 3 | 5.42 | 5.20 | 5.43 | 5.10 | 5.93 | 5.18 | 5.57 | 5.40 | 3.68 | 3.88 | 6.33 | 5.20 | 5.69 | 5.65 | 1.98 | 5.81 | 5.04 | 5.37 | 5.44 | 5.24 | 5.44 | 5.16 | 5.01 | 3.66 | 4.83 | 5.11 | 4.96 | 4.21 | 4.61 | 5.14 | 5.37 | 5.37 | 5.56 |
| mz420.272/79.6s | 11 | 2 | 5.42 | 5.10 | 5.31 | 5.10 | 5.93 | 5.38 | 5.81 | 5.40 | 5.88 | 5.62 | 6.33 | 5.84 | 5.86 | 1.34 | 5.73 | 1.29 | 4.57 | 5.37 | 5.38 | 5.13 | 1.50 | 5.41 | 5.78 | 5.46 | 5.51 | 6.20 | 5.22 | 5.38 | 5.38 | 5.38 | 5.84 | 5.62 | 5.67 |
| mz455.959/79.7s | 7 | 3 | 4.95 | 5.20 | 5.43 | 5.10 | 5.93 | 5.38 | 2.10 | 1.64 | 5.88 | 5.58 | 6.02 | 5.84 | 5.86 | 5.17 | 5.81 | 5.81 | 5.04 | 5.37 | 4.95 | 5.24 | 5.28 | 5.41 | 4.58 | 5.31 | 5.51 | 4.76 | 5.31 | 5.38 | 5.22 | 5.66 | 5.84 | 5.84 | 5.67 |
| **Supplementary Table 2.** Continued. | | | | | | | | | | | | | | | | | | | | | | | | | | | | | | | | | | | |
| **Feature** | **Cluster** | **Charge** | ***Pedobacter* sp. strain** | | | | | | | | | | | | | | | | | | | | | | | | | | | | | | | | |
|  |  |  | **DSM19033** | **DSM19036** | **JCM2171** | **LMG21415** | **LMG29220** | **UP508** | **UP509** | **UP579** | **UP621** | **UP640** | **UP696** | **UP742** | **UP751** | **UP1184** | **UP1189** | **UP1400** | **UP1426** | **UP1427** | **UP1428** | **UP1429** | **UP1430** | **UP1431** | **UP1435** | **UP1436** | **UP1437** | **UP1439** | **UP1440** | **UP1478** | **UP1479** | **UP1634** | **UP1637** | **UP1642** | **UP1729** |
| mz429.923/80.4s | 9 | 3 | 5.07 | 4.79 | 5.26 | 4.88 | 5.43 | 5.38 | 3.32 | 5.40 | 4.48 | 4.38 | 1.57 | 4.91 | 5.77 | 4.93 | 4.68 | 5.39 | 4.61 | 5.03 | 5.07 | 4.78 | 5.38 | 5.29 | 1.45 | 4.54 | 5.36 | 1.64 | 3.55 | 4.51 | 4.52 | 5.50 | 5.09 | 5.04 | 4.72 |
| mz95.086/80.6s | 10 | 1 | 4.35 | 4.13 | 4.40 | 4.02 | 4.88 | 4.75 | 2.56 | 3.08 | 3.52 | 3.93 | 2.56 | 4.69 | 4.68 | 4.67 | 3.27 | 4.74 | 4.28 | 4.83 | 4.18 | 4.00 | 4.34 | 4.36 | 2.22 | 3.69 | 3.99 | 2.38 | 3.88 | 3.71 | 3.81 | 4.69 | 4.52 | 4.57 | 4.07 |
| mz467.289/80.8s | 9 | 3 | 5.42 | 5.13 | 5.43 | 4.74 | 5.93 | 5.31 | 2.57 | 4.81 | 5.88 | 5.62 | 1.18 | 5.84 | 5.40 | 5.28 | 4.72 | 5.44 | 4.72 | 5.30 | 5.44 | 5.24 | 4.89 | 5.22 | 0.89 | 5.46 | 5.42 | 1.02 | 4.35 | 5.38 | 5.38 | 4.80 | 5.52 | 5.84 | 5.67 |
| mz467.935/83.6s | 11 | 3 | 5.42 | 5.20 | 4.00 | 5.10 | 5.93 | 5.38 | 3.11 | 5.40 | 5.88 | 5.62 | 6.33 | 1.08 | 5.36 | 1.35 | 5.93 | 1.29 | 4.41 | 5.37 | 5.44 | 5.24 | 1.71 | 5.29 | 5.78 | 5.46 | 5.51 | 6.20 | 5.31 | 5.38 | 5.38 | 5.81 | 5.84 | 5.84 | 5.61 |
| mz713.437/85.3s | 5 | 1 | 5.42 | 5.18 | 5.43 | 2.01 | 1.48 | 1.85 | 5.99 | 5.40 | 2.11 | 2.17 | 5.89 | 4.99 | 5.86 | 5.65 | 5.93 | 5.61 | 5.04 | 5.33 | 5.44 | 5.24 | 5.35 | 5.41 | 5.78 | 2.56 | 1.55 | 6.20 | 5.16 | 5.34 | 5.38 | 5.81 | 5.84 | 5.84 | 5.67 |
| mz354.22/86.1s | 10 | 4 | 5.34 | 5.20 | 5.43 | 4.60 | 5.54 | 5.38 | 5.28 | 4.92 | 2.13 | 2.01 | 2.97 | 4.96 | 5.41 | 4.81 | 3.09 | 5.81 | 4.56 | 5.28 | 5.29 | 4.46 | 4.94 | 4.95 | 3.49 | 1.90 | 4.38 | 3.33 | 4.31 | 2.31 | 3.35 | 5.42 | 5.46 | 5.72 | 5.62 |
| mz674.426/86.3s | 14 | 1 | 5.42 | 5.20 | 5.43 | 5.10 | 5.93 | 5.36 | 5.87 | 5.40 | 5.88 | 5.62 | 6.33 | 1.36 | 5.86 | 1.52 | 5.93 | 1.33 | 5.04 | 5.37 | 5.44 | 5.24 | 1.67 | 5.41 | 5.78 | 5.46 | 5.51 | 6.20 | 5.31 | 5.38 | 5.38 | 5.81 | 5.84 | 5.84 | 5.67 |
| mz132.102/86.4s | - | 1 | 2.88 | 2.42 | 2.60 | 2.09 | 2.03 | 2.49 | 3.59 | 2.85 | 2.64 | 2.99 | 3.70 | 1.98 | 3.59 | 1.98 | 3.06 | 1.88 | 2.34 | 2.73 | 2.66 | 2.51 | 3.81 | 2.61 | 3.09 | 2.47 | 1.90 | 3.57 | 2.53 | 2.79 | 2.72 | 3.55 | 3.01 | 3.31 | 2.88 |
| mz471.958/87s | 10 | 3 | 5.04 | 5.20 | 4.83 | 5.10 | 5.93 | 5.38 | 4.85 | 4.33 | 1.93 | 1.79 | 2.85 | 2.83 | 5.73 | 5.65 | 2.91 | 5.74 | 5.04 | 5.37 | 5.44 | 5.24 | 5.44 | 5.41 | 3.30 | 1.73 | 5.07 | 2.94 | 4.64 | 2.03 | 3.22 | 5.81 | 5.61 | 5.84 | 5.67 |
| mz706.398/87.7s | 8 | 1 | 5.42 | 5.20 | 5.38 | 5.10 | 5.80 | 5.38 | 6.17 | 5.40 | 5.75 | 5.62 | 6.33 | 5.84 | 5.86 | 5.41 | 5.93 | 5.69 | 4.91 | 5.22 | 5.44 | 5.24 | 5.42 | 5.41 | 5.78 | 5.43 | 5.51 | 5.77 | 5.08 | 5.38 | 5.38 | 5.81 | 5.84 | 5.84 | 2.50 |
| mz379.217/88.2s | 3 | 3 | 5.42 | 5.20 | 5.27 | 4.06 | 5.00 | 4.34 | 5.44 | 5.40 | 5.88 | 4.71 | 5.86 | 5.84 | 5.59 | 5.65 | 1.98 | 5.81 | 4.13 | 4.75 | 5.21 | 4.42 | 5.44 | 5.41 | 5.72 | 4.51 | 5.21 | 5.60 | 5.26 | 5.10 | 5.38 | 5.54 | 5.55 | 5.20 | 5.16 |
| mz460.63/89.7s | 7 | 3 | 5.32 | 5.20 | 5.29 | 5.10 | 5.93 | 5.38 | 3.45 | 1.56 | 5.88 | 5.11 | 6.33 | 5.84 | 5.86 | 5.65 | 5.93 | 5.81 | 4.49 | 5.28 | 5.44 | 5.24 | 5.42 | 5.41 | 5.78 | 5.46 | 5.51 | 6.17 | 5.31 | 4.98 | 5.38 | 5.69 | 5.71 | 5.84 | 5.25 |
| mz427.28/89.8s | 11 | 2 | 5.42 | 5.14 | 5.43 | 5.10 | 5.93 | 5.38 | 6.17 | 5.30 | 5.66 | 5.62 | 6.33 | 5.59 | 5.86 | 1.05 | 5.60 | 1.05 | 4.31 | 5.31 | 5.44 | 5.24 | 1.32 | 5.41 | 5.57 | 5.46 | 5.51 | 6.13 | 5.31 | 5.24 | 4.93 | 5.81 | 5.84 | 5.80 | 5.67 |
| mz471.958/91.5s | 10 | 3 | 4.88 | 5.16 | 4.35 | 5.02 | 5.31 | 4.27 | 4.27 | 3.71 | 1.80 | 1.65 | 2.55 | 4.68 | 4.53 | 4.92 | 2.00 | 4.99 | 4.87 | 5.37 | 4.95 | 5.24 | 4.70 | 4.89 | 2.86 | 1.60 | 4.04 | 2.69 | 4.30 | 1.88 | 2.63 | 4.80 | 4.73 | 5.00 | 4.51 |
| mz368.541/93.4s | 2 | 3 | 5.42 | 5.20 | 5.43 | 2.88 | 5.16 | 2.10 | 6.16 | 5.05 | 1.76 | 2.09 | 4.94 | 4.96 | 5.66 | 5.60 | 2.94 | 5.81 | 5.04 | 5.00 | 4.63 | 5.19 | 5.41 | 5.38 | 5.63 | 2.17 | 4.97 | 5.71 | 5.22 | 1.82 | 1.84 | 5.16 | 5.84 | 5.84 | 5.67 |
| mz457.93/94.7s | 11 | 3 | 5.33 | 5.20 | 2.82 | 4.93 | 5.93 | 4.97 | 6.17 | 5.40 | 5.11 | 5.62 | 6.33 | 2.01 | 5.86 | 2.07 | 5.54 | 2.04 | 4.77 | 5.37 | 5.38 | 5.24 | 2.46 | 5.41 | 5.14 | 5.46 | 5.51 | 6.20 | 5.31 | 5.38 | 5.38 | 5.81 | 5.70 | 5.54 | 5.67 |
| mz472.606/95.2s | 11 | 3 | 5.29 | 5.20 | 3.85 | 5.10 | 5.93 | 5.38 | 6.17 | 5.40 | 5.88 | 5.62 | 6.33 | 2.04 | 5.68 | 2.27 | 5.93 | 2.24 | 4.77 | 5.37 | 5.44 | 5.24 | 3.12 | 5.41 | 5.78 | 5.46 | 5.51 | 6.20 | 5.31 | 5.38 | 5.38 | 5.55 | 5.84 | 5.68 | 5.49 |
| mz335.19/96.2s | 8 | 2 | 4.69 | 5.08 | 4.87 | 4.56 | 5.86 | 5.31 | 6.02 | 4.90 | 5.43 | 5.62 | 5.87 | 5.25 | 5.36 | 5.65 | 5.21 | 5.03 | 4.50 | 5.23 | 5.22 | 4.67 | 4.93 | 5.14 | 5.13 | 4.97 | 5.51 | 5.21 | 5.17 | 5.38 | 5.00 | 5.75 | 5.84 | 5.57 | 1.84 |
| mz418.595/96.3s | 7 | 3 | 5.40 | 5.20 | 5.43 | 4.97 | 5.93 | 5.38 | 4.26 | 1.50 | 5.88 | 5.62 | 5.51 | 5.84 | 5.86 | 5.65 | 5.93 | 5.81 | 5.04 | 5.28 | 5.44 | 5.24 | 5.44 | 5.41 | 5.69 | 5.46 | 5.51 | 6.20 | 5.31 | 5.38 | 5.38 | 5.81 | 5.84 | 5.84 | 5.22 |
| mz465.304/96.3s | - | 3 | 5.42 | 5.20 | 5.43 | 5.10 | 5.93 | 5.38 | 6.17 | 0.79 | 5.88 | 5.62 | 6.33 | 5.84 | 5.86 | 5.65 | 5.93 | 5.79 | 5.04 | 5.37 | 5.44 | 5.24 | 5.44 | 5.41 | 5.78 | 5.46 | 5.51 | 6.05 | 5.15 | 5.38 | 5.38 | 5.81 | 5.84 | 5.84 | 5.67 |
| mz383.889/97.6s | 3 | 3 | 5.42 | 5.20 | 5.43 | 4.23 | 5.93 | 4.26 | 6.13 | 5.37 | 4.27 | 4.09 | 6.33 | 5.84 | 5.86 | 5.65 | 1.81 | 5.81 | 5.04 | 5.37 | 5.44 | 5.03 | 5.44 | 5.41 | 5.78 | 4.39 | 5.51 | 6.20 | 5.31 | 4.01 | 5.38 | 5.81 | 5.84 | 5.84 | 5.67 |
| mz373.886/99.8s | 3 | 3 | 5.42 | 5.06 | 5.43 | 4.43 | 5.89 | 4.47 | 6.17 | 5.38 | 4.78 | 4.71 | 6.29 | 5.47 | 5.71 | 5.65 | 1.76 | 5.81 | 5.04 | 5.37 | 5.44 | 5.24 | 5.44 | 5.41 | 5.78 | 5.08 | 5.39 | 6.17 | 5.31 | 4.95 | 5.38 | 5.81 | 5.67 | 5.84 | 5.67 |
| **Supplementary Table 2.** Continued. | | | | | | | | | | | | | | | | | | | | | | | | | | | | | | | | | | | |
| **Feature** | **Cluster** | **Charge** | ***Pedobacter* sp. strain** | | | | | | | | | | | | | | | | | | | | | | | | | | | | | | | | |
|  |  |  | **DSM19033** | **DSM19036** | **JCM2171** | **LMG21415** | **LMG29220** | **UP508** | **UP509** | **UP579** | **UP621** | **UP640** | **UP696** | **UP742** | **UP751** | **UP1184** | **UP1189** | **UP1400** | **UP1426** | **UP1427** | **UP1428** | **UP1429** | **UP1430** | **UP1431** | **UP1435** | **UP1436** | **UP1437** | **UP1439** | **UP1440** | **UP1478** | **UP1479** | **UP1634** | **UP1637** | **UP1642** | **UP1729** |
| mz669.373/99.8s | 8 | 1 | 5.42 | 5.20 | 5.43 | 4.93 | 5.93 | 5.38 | 5.84 | 5.40 | 5.88 | 5.62 | 6.33 | 4.88 | 5.46 | 5.13 | 5.93 | 4.77 | 5.04 | 5.37 | 5.44 | 5.24 | 5.44 | 5.41 | 5.78 | 5.46 | 5.51 | 6.20 | 5.31 | 5.38 | 5.38 | 5.81 | 5.50 | 5.84 | 1.96 |
| mz462.603/100.1s | - | 3 | 5.42 | 5.20 | 1.18 | 5.10 | 5.93 | 5.38 | 5.70 | 5.40 | 5.88 | 5.62 | 6.33 | 2.66 | 5.86 | 3.63 | 5.93 | 4.55 | 5.04 | 5.37 | 5.44 | 5.24 | 5.44 | 5.41 | 5.78 | 5.46 | 5.51 | 6.20 | 5.31 | 5.38 | 5.38 | 5.81 | 5.84 | 5.84 | 5.67 |
| mz132.102/100.6s | - | 1 | 3.21 | 3.00 | 3.04 | 2.52 | 3.36 | 3.40 | 3.92 | 3.14 | 3.75 | 4.08 | 4.05 | 2.03 | 5.86 | 3.48 | 3.52 | 2.04 | 2.70 | 3.30 | 3.11 | 3.41 | 5.44 | 2.95 | 3.56 | 2.99 | 3.06 | 3.67 | 3.57 | 3.05 | 3.46 | 3.52 | 4.07 | 3.38 | 3.20 |
| mz688.442/100.6s | - | 1 | 5.42 | 5.20 | 5.43 | 5.10 | 5.93 | 5.38 | 6.17 | 5.40 | 5.88 | 5.62 | 6.33 | 1.59 | 5.86 | 1.96 | 5.93 | 1.60 | 5.04 | 5.37 | 5.44 | 5.24 | 2.12 | 5.41 | 5.78 | 5.46 | 5.51 | 6.20 | 5.31 | 5.38 | 5.38 | 5.81 | 5.84 | 5.84 | 5.67 |
| mz138.055/102.5s | - | 1 | 2.02 | 1.84 | 2.07 | 1.85 | 2.61 | 2.33 | 2.76 | 2.00 | 2.81 | 2.48 | 2.91 | 2.56 | 2.15 | 2.24 | 2.55 | 2.59 | 1.86 | 1.89 | 1.97 | 1.80 | 2.21 | 2.02 | 2.37 | 2.40 | 2.43 | 2.94 | 1.89 | 2.28 | 2.33 | 2.35 | 2.45 | 2.48 | 2.34 |
| mz367.882/103.1s | 4 | 3 | 5.40 | 5.20 | 5.43 | 5.10 | 1.81 | 4.81 | 6.03 | 5.40 | 5.88 | 5.62 | 6.33 | 5.51 | 5.86 | 5.65 | 5.93 | 5.81 | 5.04 | 5.37 | 4.67 | 5.13 | 5.40 | 5.04 | 5.78 | 5.46 | 2.73 | 6.20 | 5.31 | 5.38 | 5.38 | 5.75 | 5.84 | 5.84 | 5.67 |
| mz462.602/103.3s | 11 | 3 | 5.42 | 5.20 | 1.50 | 5.10 | 5.93 | 5.38 | 6.14 | 5.13 | 5.85 | 5.62 | 6.33 | 2.21 | 5.86 | 2.40 | 5.93 | 2.30 | 4.60 | 5.37 | 5.44 | 5.24 | 2.91 | 5.41 | 5.68 | 5.19 | 5.51 | 6.20 | 5.31 | 5.38 | 5.38 | 5.81 | 5.84 | 5.84 | 5.67 |
| mz373.214/104.8s | 2 | 3 | 5.28 | 4.88 | 5.38 | 1.64 | 5.93 | 5.38 | 5.82 | 5.40 | 0.90 | 1.00 | 4.38 | 4.73 | 5.67 | 5.65 | 2.38 | 5.44 | 5.04 | 5.37 | 5.44 | 4.80 | 5.35 | 5.41 | 5.78 | 1.17 | 4.45 | 6.09 | 4.30 | 0.97 | 1.14 | 4.39 | 5.78 | 5.06 | 5.60 |
| mz476.256/107.4s | 13 | 3 | 5.42 | 5.20 | 5.43 | 5.10 | 5.93 | 5.38 | 6.11 | 5.40 | 5.88 | 5.62 | 6.33 | 5.84 | 1.42 | 5.65 | 5.93 | 5.81 | 5.04 | 5.37 | 5.44 | 5.11 | 5.44 | 5.41 | 5.78 | 5.37 | 5.51 | 6.20 | 5.31 | 5.38 | 5.38 | 1.40 | 1.40 | 1.37 | 1.42 |
| mz467.277/107.9s | - | 3 | 5.42 | 5.20 | 0.59 | 5.10 | 4.92 | 4.85 | 5.67 | 5.22 | 5.88 | 5.62 | 6.33 | 4.66 | 5.86 | 5.11 | 5.93 | 5.81 | 5.04 | 5.37 | 5.44 | 5.04 | 5.44 | 5.41 | 5.78 | 5.46 | 5.51 | 6.20 | 5.17 | 4.63 | 5.07 | 5.81 | 5.84 | 5.84 | 5.23 |
| mz342.199/108.3s | 8 | 2 | 5.31 | 5.20 | 5.43 | 4.94 | 5.93 | 5.28 | 6.17 | 5.24 | 5.88 | 5.62 | 5.84 | 5.35 | 5.86 | 5.65 | 5.72 | 5.23 | 4.90 | 5.08 | 5.01 | 5.24 | 5.44 | 5.41 | 5.50 | 5.46 | 5.47 | 6.20 | 5.31 | 5.36 | 5.38 | 5.73 | 5.84 | 5.21 | 1.28 |
| mz373.214/108.7s | 1 | 3 | 5.36 | 5.00 | 5.43 | 5.10 | 5.93 | 1.16 | 6.17 | 5.40 | 5.88 | 5.62 | 6.33 | 5.84 | 5.67 | 5.65 | 5.93 | 5.68 | 4.93 | 5.37 | 5.44 | 5.24 | 5.39 | 5.41 | 5.78 | 5.46 | 5.51 | 6.20 | 5.17 | 5.38 | 5.38 | 5.81 | 5.84 | 5.84 | 5.52 |
| mz378.558/108.9s | 3 | 3 | 5.40 | 5.20 | 5.43 | 5.10 | 5.93 | 4.46 | 6.17 | 5.40 | 5.88 | 5.33 | 5.64 | 5.84 | 5.86 | 5.65 | 1.56 | 5.81 | 5.04 | 5.37 | 5.32 | 4.73 | 5.44 | 5.41 | 5.78 | 4.43 | 5.51 | 6.20 | 5.31 | 4.13 | 5.38 | 5.81 | 5.84 | 5.76 | 5.67 |
| mz301.212/109.4s | - | 1 | 5.42 | 5.20 | 2.36 | 1.54 | 5.93 | 5.38 | 6.15 | 5.18 | 5.32 | 5.62 | 6.33 | 5.84 | 1.84 | 5.65 | 5.93 | 5.67 | 2.83 | 3.19 | 3.31 | 3.58 | 5.34 | 3.21 | 5.78 | 4.81 | 5.32 | 6.20 | 2.31 | 5.38 | 5.25 | 2.35 | 2.40 | 2.47 | 5.28 |
| mz372.554/113.5s | 4 | 3 | 5.42 | 5.17 | 5.11 | 5.10 | 1.06 | 5.38 | 6.17 | 5.40 | 5.72 | 4.94 | 6.33 | 5.84 | 5.57 | 5.54 | 4.97 | 5.81 | 5.04 | 4.54 | 5.20 | 5.19 | 5.44 | 4.54 | 5.78 | 5.03 | 5.51 | 6.08 | 5.15 | 5.04 | 5.38 | 5.81 | 5.79 | 5.75 | 5.67 |
| mz377.886/114.5s | 2 | 3 | 5.42 | 5.15 | 5.24 | 1.43 | 5.93 | 5.38 | 5.26 | 5.40 | 0.84 | 0.89 | 4.70 | 4.95 | 5.86 | 4.87 | 2.43 | 5.76 | 5.04 | 5.37 | 4.99 | 5.07 | 5.25 | 5.41 | 5.18 | 1.08 | 4.88 | 5.99 | 4.64 | 0.85 | 0.91 | 4.65 | 5.76 | 5.84 | 5.67 |
| mz480.928/115.9s | 13 | 3 | 5.42 | 5.20 | 5.24 | 4.45 | 5.93 | 5.38 | 6.17 | 5.40 | 5.83 | 5.62 | 6.33 | 5.84 | 1.41 | 5.62 | 5.93 | 5.38 | 5.04 | 5.37 | 5.44 | 5.24 | 4.88 | 5.41 | 5.78 | 5.46 | 5.40 | 6.20 | 5.31 | 5.38 | 5.38 | 1.40 | 1.36 | 1.36 | 0.78 |
| mz377.886/118.5s | 1 | 3 | 5.38 | 5.14 | 5.21 | 5.10 | 5.93 | 1.07 | 6.17 | 4.67 | 5.88 | 5.62 | 6.33 | 5.23 | 5.86 | 5.65 | 5.93 | 5.77 | 4.92 | 5.37 | 5.44 | 5.24 | 5.25 | 5.11 | 5.78 | 5.46 | 5.51 | 6.00 | 5.02 | 5.38 | 5.38 | 5.81 | 5.84 | 5.84 | 5.67 |
| mz381.886/124.3s | 1 | 3 | 5.30 | 5.20 | 5.43 | 5.10 | 5.57 | 1.65 | 5.42 | 5.22 | 5.88 | 5.62 | 6.27 | 5.70 | 5.86 | 5.19 | 5.93 | 5.74 | 5.04 | 5.21 | 5.15 | 5.00 | 5.44 | 5.41 | 5.78 | 5.46 | 5.51 | 6.20 | 5.12 | 5.38 | 5.38 | 5.81 | 5.84 | 5.84 | 5.67 |
| mz362.551/127.5s | 4 | 3 | 5.42 | 5.20 | 4.74 | 5.10 | 1.28 | 5.38 | 6.17 | 5.40 | 5.88 | 5.62 | 6.33 | 5.84 | 5.86 | 5.65 | 5.93 | 5.81 | 5.04 | 1.14 | 1.07 | 1.26 | 5.44 | 1.09 | 5.78 | 5.46 | 1.94 | 6.12 | 5.12 | 4.97 | 5.38 | 5.81 | 5.84 | 5.84 | 5.67 |
| mz382.557/128.9s | 2 | 3 | 5.42 | 5.20 | 5.43 | 3.38 | 5.93 | 2.13 | 5.08 | 5.40 | 1.94 | 2.23 | 6.33 | 5.84 | 5.86 | 5.65 | 3.68 | 5.81 | 5.04 | 5.27 | 5.21 | 5.24 | 5.44 | 5.41 | 5.78 | 2.53 | 5.49 | 6.20 | 5.31 | 2.11 | 2.36 | 5.67 | 5.84 | 5.71 | 5.67 |
| mz367.883/129.8s | 2 | 3 | 5.42 | 5.20 | 5.43 | 1.88 | 5.93 | 1.67 | 5.72 | 4.89 | 1.20 | 1.15 | 5.05 | 5.51 | 5.81 | 5.65 | 3.87 | 5.81 | 5.04 | 5.37 | 5.44 | 5.24 | 5.44 | 5.41 | 5.74 | 1.04 | 4.02 | 6.13 | 4.68 | 1.33 | 1.37 | 5.54 | 5.47 | 5.80 | 5.67 |
| mz367.882/132.4s | 1 | 3 | 5.42 | 5.20 | 5.43 | 5.10 | 5.93 | 1.55 | 5.73 | 4.88 | 5.88 | 5.62 | 5.62 | 5.81 | 5.86 | 5.65 | 5.93 | 5.81 | 4.73 | 5.37 | 5.44 | 5.24 | 5.44 | 5.41 | 5.78 | 5.46 | 5.51 | 6.13 | 5.14 | 5.38 | 5.38 | 5.53 | 5.47 | 5.81 | 5.67 |
| **Supplementary Table 2.** Continued. | | | | | | | | | | | | | | | | | | | | | | | | | | | | | | | | | | | |
| **Feature** | **Cluster** | **Charge** | ***Pedobacter* sp. strain** | | | | | | | | | | | | | | | | | | | | | | | | | | | | | | | | |
|  |  |  | **DSM19033** | **DSM19036** | **JCM2171** | **LMG21415** | **LMG29220** | **UP508** | **UP509** | **UP579** | **UP621** | **UP640** | **UP696** | **UP742** | **UP751** | **UP1184** | **UP1189** | **UP1400** | **UP1426** | **UP1427** | **UP1428** | **UP1429** | **UP1430** | **UP1431** | **UP1435** | **UP1436** | **UP1437** | **UP1439** | **UP1440** | **UP1478** | **UP1479** | **UP1634** | **UP1637** | **UP1642** | **UP1729** |
| mz382.558/133s | 1 | 3 | 5.42 | 5.20 | 5.43 | 5.10 | 5.93 | 1.31 | 6.17 | 5.40 | 5.88 | 5.62 | 6.28 | 5.63 | 5.86 | 5.65 | 5.93 | 5.81 | 5.04 | 5.26 | 5.38 | 5.07 | 5.44 | 5.41 | 5.78 | 5.46 | 5.32 | 6.20 | 5.31 | 5.38 | 5.38 | 5.81 | 5.84 | 5.84 | 5.67 |
| mz367.225/135.1s | 4 | 3 | 4.81 | 5.20 | 4.96 | 5.10 | 0.77 | 5.38 | 5.81 | 5.40 | 5.88 | 5.62 | 6.33 | 5.84 | 5.86 | 5.63 | 5.93 | 5.81 | 5.04 | 0.44 | 0.43 | 0.54 | 4.63 | 0.41 | 5.45 | 5.46 | 0.71 | 5.48 | 5.31 | 5.38 | 5.02 | 5.81 | 5.84 | 5.51 | 5.67 |
| mz372.554/139.2s | 2 | 3 | 5.42 | 5.18 | 5.43 | 1.66 | 4.06 | 5.84 | 5.26 | 4.80 | 1.02 | 0.96 | 5.49 | 5.11 | 5.82 | 5.05 | 3.75 | 5.81 | 5.04 | 5.37 | 4.98 | 4.37 | 5.44 | 4.93 | 5.78 | 0.87 | 3.70 | 5.60 | 5.31 | 1.16 | 1.16 | 4.88 | 5.84 | 5.52 | 5.67 |
| mz372.554/141.6s | 1 | 3 | 5.81 | 5.81 | 5.81 | 5.81 | 5.81 | 0.00 | 5.84 | 5.84 | 5.84 | 5.84 | 5.84 | 5.84 | 5.84 | 5.81 | 5.81 | 5.81 | 5.81 | 5.81 | 5.81 | 5.81 | 5.81 | 5.81 | 5.81 | 5.81 | 5.81 | 5.81 | 5.81 | 5.81 | 5.81 | 5.81 | 5.81 | 5.81 | 5.81 |
| mz387.23/143s | 1 | 3 | 5.27 | 5.08 | 5.36 | 5.10 | 5.25 | 1.26 | 6.17 | 5.40 | 3.98 | 5.62 | 6.22 | 5.84 | 5.55 | 5.65 | 5.93 | 5.81 | 5.04 | 5.37 | 5.10 | 5.24 | 5.44 | 4.77 | 5.54 | 5.46 | 5.34 | 6.20 | 5.31 | 5.38 | 5.38 | 5.81 | 5.84 | 5.84 | 5.55 |
| mz376.553/148.4s | 1 | 3 | 5.42 | 5.20 | 5.43 | 4.24 | 5.93 | 1.61 | 5.25 | 5.14 | 2.76 | 2.85 | 6.28 | 5.84 | 5.86 | 5.65 | 5.93 | 5.81 | 5.04 | 5.37 | 5.44 | 5.24 | 5.44 | 5.41 | 5.78 | 2.90 | 4.94 | 6.20 | 5.31 | 3.41 | 2.96 | 5.81 | 5.84 | 5.84 | 5.67 |
| mz377.225/156.8s | 1 | 3 | 5.36 | 5.20 | 5.43 | 4.52 | 5.93 | 1.65 | 5.51 | 4.89 | 2.46 | 2.74 | 6.33 | 5.72 | 5.71 | 5.65 | 5.37 | 5.66 | 5.04 | 5.37 | 4.85 | 4.29 | 5.42 | 5.41 | 5.78 | 2.67 | 4.55 | 6.20 | 5.31 | 2.83 | 3.47 | 5.81 | 5.34 | 5.84 | 5.67 |
| mz381.898/167s | 1 | 3 | 5.33 | 5.20 | 5.43 | 4.30 | 5.93 | 1.50 | 6.02 | 4.91 | 4.07 | 3.85 | 5.80 | 5.84 | 5.86 | 5.33 | 5.67 | 5.81 | 5.04 | 5.37 | 4.85 | 5.11 | 5.44 | 5.41 | 5.78 | 3.58 | 5.51 | 6.20 | 5.31 | 4.10 | 4.87 | 5.81 | 5.77 | 5.56 | 5.67 |
| mz352.252/167.6s | - | 1 | 4.31 | 3.01 | 3.10 | 2.43 | 4.29 | 3.78 | 3.38 | 1.65 | 5.27 | 4.46 | 4.02 | 3.18 | 4.92 | 3.79 | 4.16 | 3.26 | 2.38 | 2.04 | 1.95 | 2.19 | 3.49 | 2.04 | 3.57 | 4.44 | 5.34 | 3.28 | 1.29 | 4.25 | 4.43 | 4.35 | 4.12 | 4.11 | 4.35 |


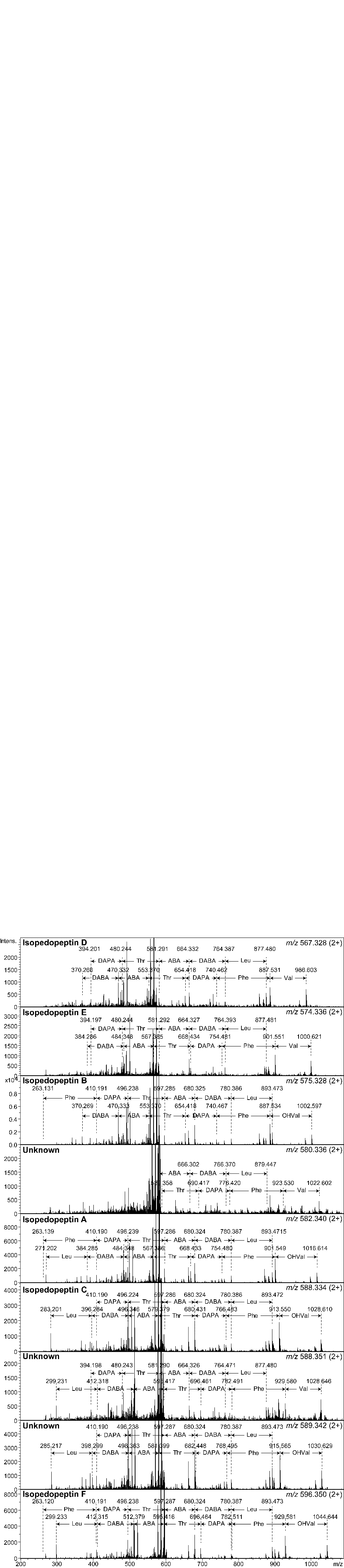


### Supplementary Figure 1. MSMS spectra from analysis of isopedopeptins A-F and three unknown isopedopeptins from UP508 (cluster 1) after ring-opening with NaOMe in MeOH. In each spectrum, B-ions with corresponding amino acid sequence are shown on bottom, and Y-ions with corresponding amino acid sequence are shown on top. Precursor ions are shown in the top right corner in each spectrum.


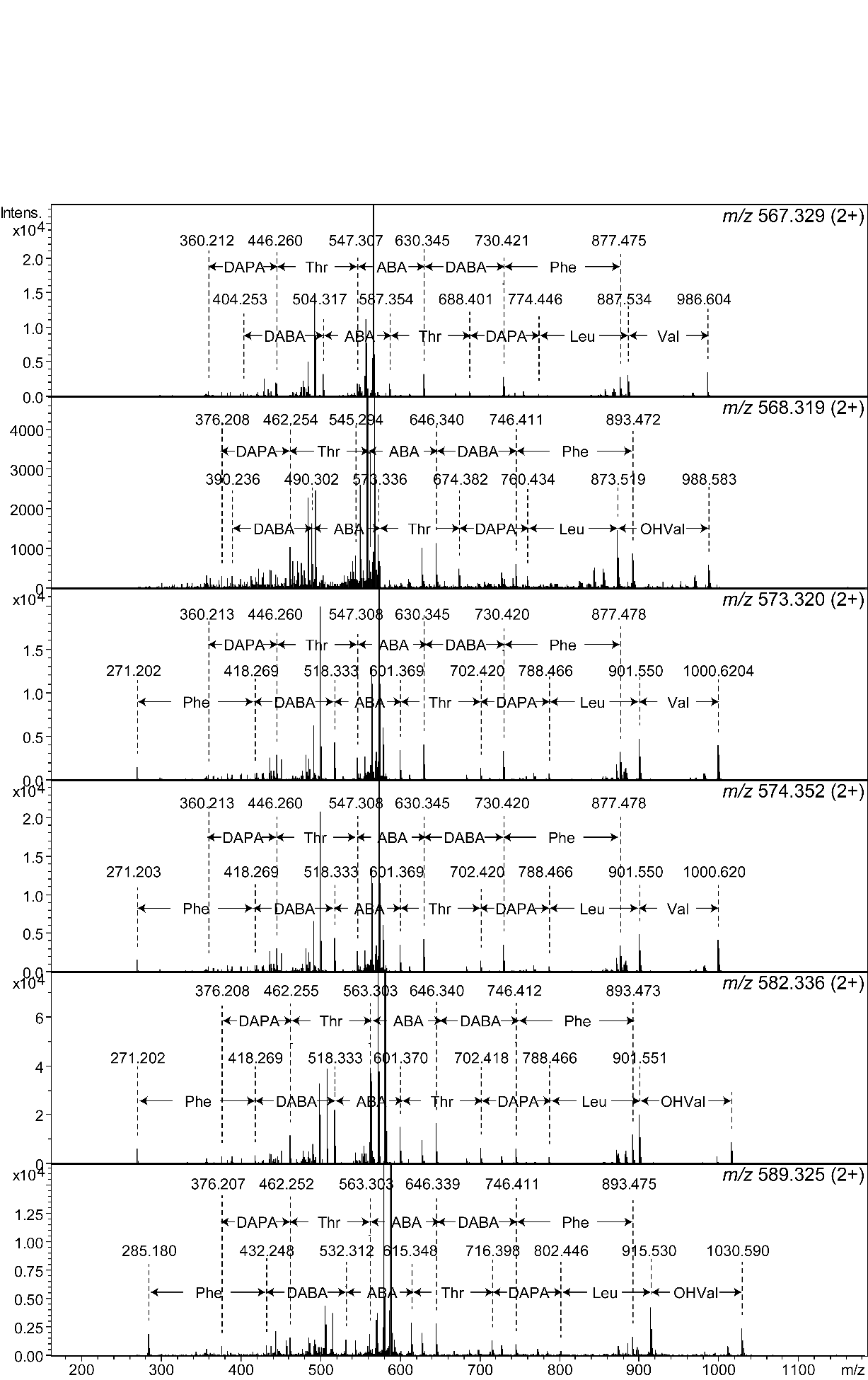


### Supplementary Figure 2. MSMS spectra from analysis of six peptides from UP640 (cluster 2) after ring-opening with NaOMe in MeOH. In each spectrum, B-ions with corresponding amino acid sequence are shown on bottom, and Y-ions with corresponding amino acid sequence are shown on top. Precursor ions are shown in the top right corner in each spectrum.


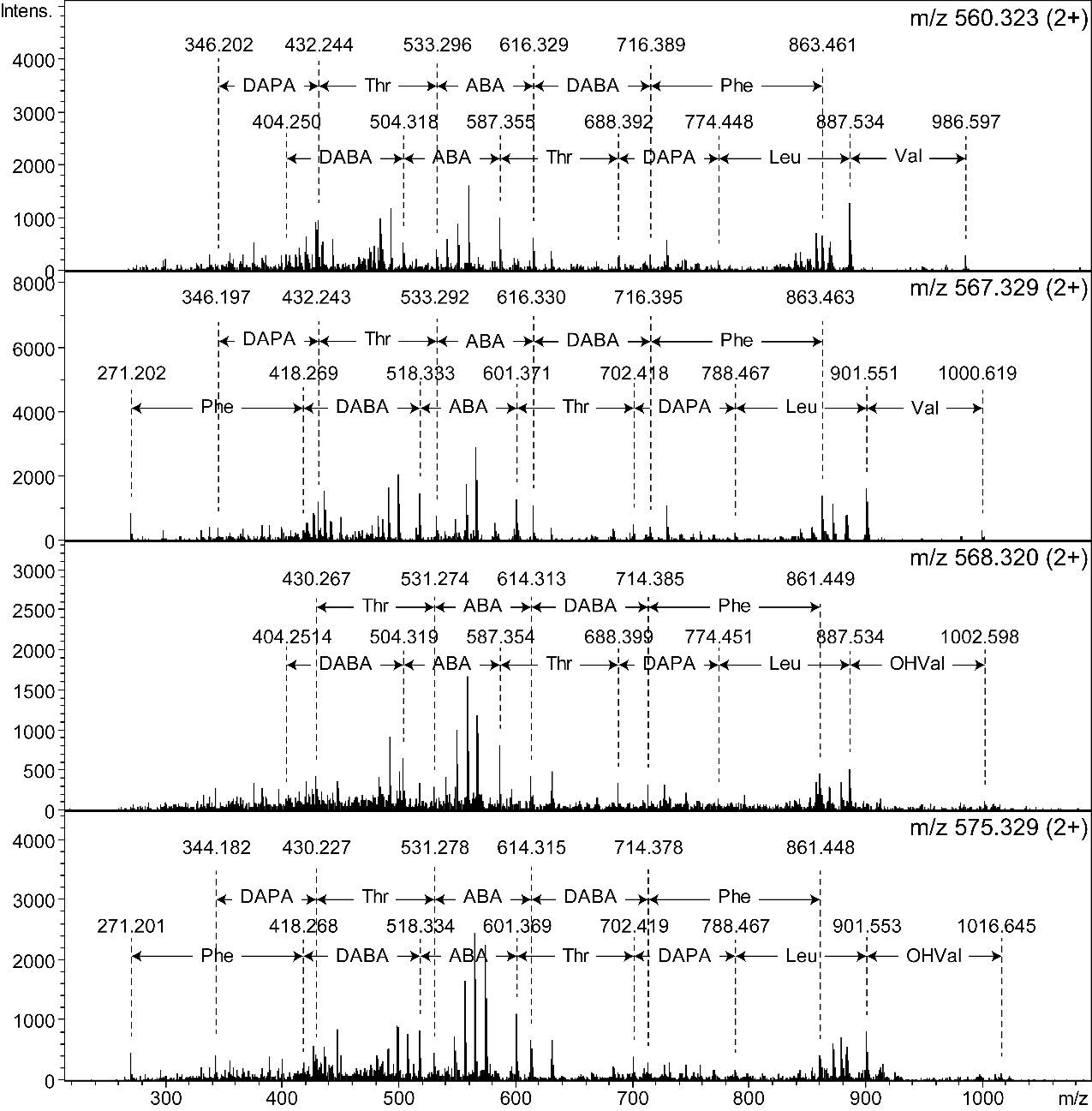


### Supplementary Figure 3. MSMS spectra from analysis of four peptides from UP1189 (cluster 3) without ring-opening with NaOMe in MeOH. In each spectrum, B-ions with corresponding amino acid sequence are shown on bottom, and Y-ions with corresponding amino acid sequence are shown on top. Precursor ions are shown in the top right corner in each spectrum.


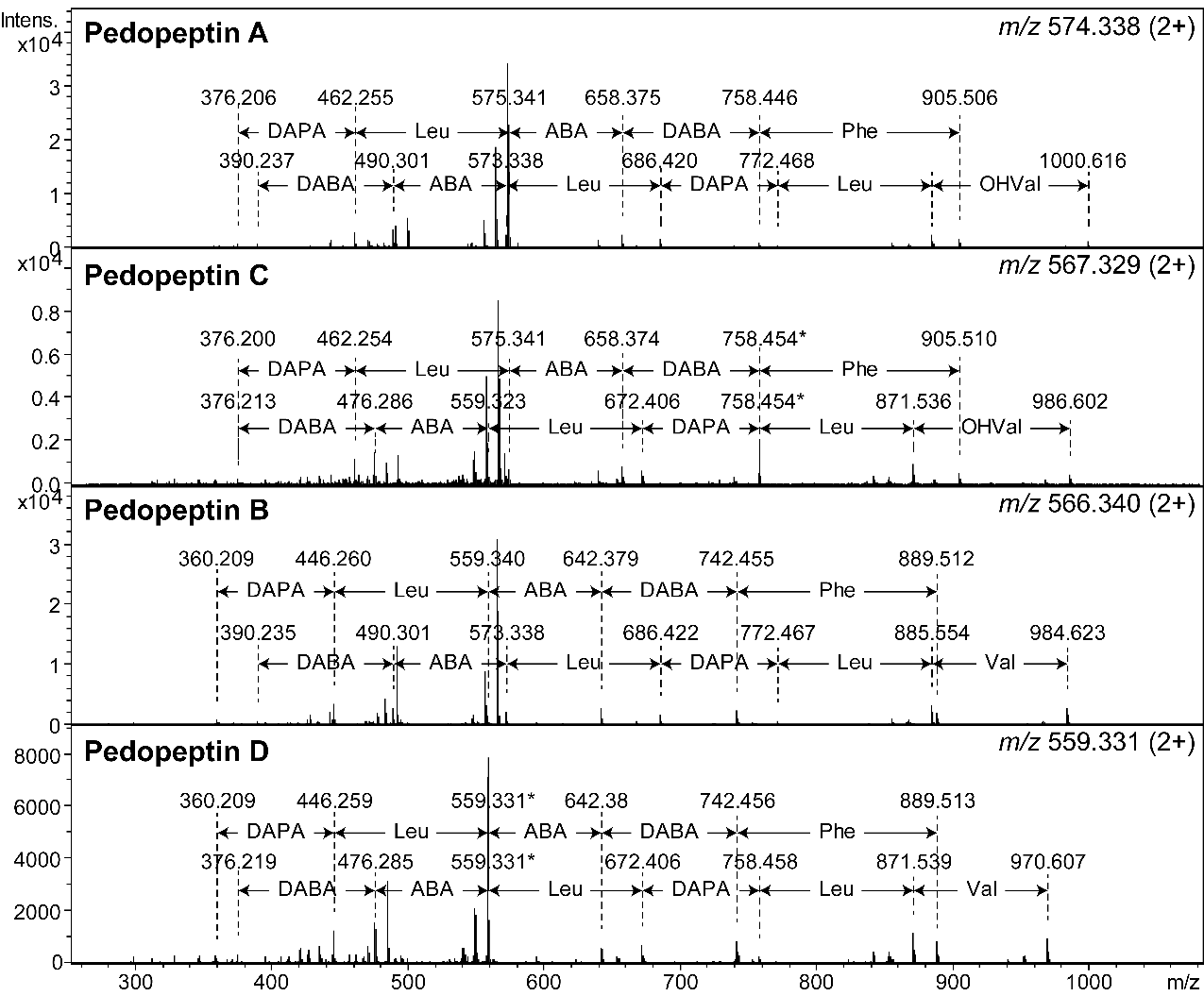


### Supplementary Figure 4. MSMS spectra from analysis of pedopeptin A-D (cluster 4) after ring-opening with NaOMe in MeOH. In each spectrum, B-ions with corresponding amino acid sequence are shown on bottom, and Y-ions with corresponding amino acid sequence are shown on top. Precursor ions are shown in the top right corner in each spectrum.


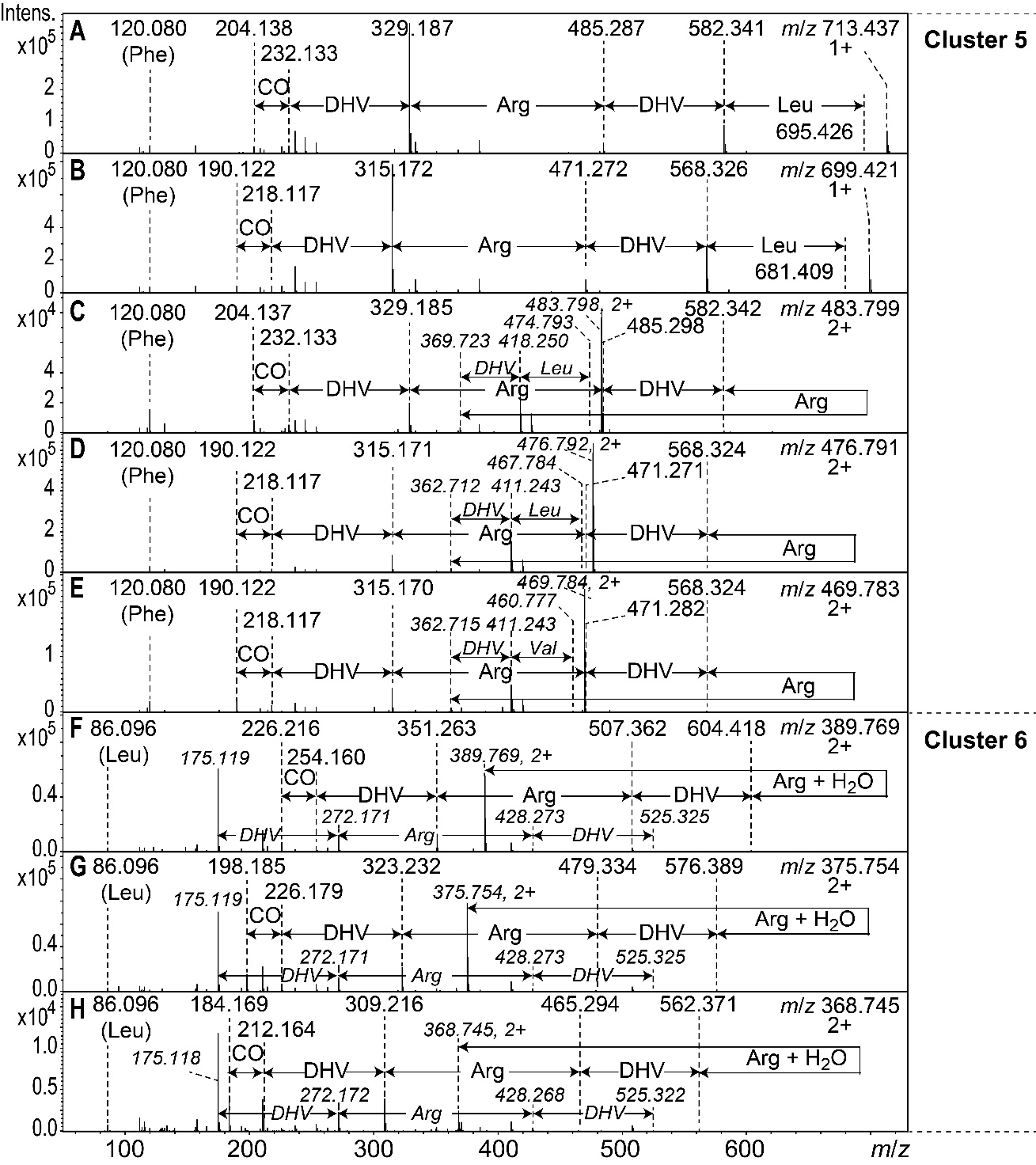


### Supplementary Figure 5. MSMS spectra of peptides in cluster 5 and 6. Only B-type ions are shown in spectra A-E along with proposed amino acid sequences, and double charged ions are shown in italic typeface. In spectra F-H both B-type and Y-type ions are shown, with Y-type ions shown in italic typeface. The precursor ions are shown in the top-right corners of the spectra. (DHV = dehydrovaline)

### **Isolation and characterization of two cluster 5 peptides (A: *m/z* 699.421/t_R_ 72.5 s and B: *m/z* 713.437/t_R_ 85.3 s).**

The peptide with *m/z* 699.421/t_R_ 72.5 s was isolated from cultures of UP508. Extracts of adsorbent bags from 23 cultures (as in main text) was dried, reconstituted in 10 mL 50% MeCN, and fractionated by reversed-phase HPLC (Luna Omega PS C18 column, 21.2 × 100 mm, 5 μm, 10 × 1 mL injected) using a gradient of MeCN in H_2_O with 0.2% formic acid (10-95% MeCN in 10 min followed by a hold at 95% for 10 minutes), with collection of 2-mL fractions. Fractions 52-53 were collected, and rechromatographed (same column as above) with a gradient of MeCN in H_2_O with 0.2% formic acid (15-30% MeCN in 23 min followed by 30-95% MeCN in 1 min and a hold on 95% MeCN for 5 min). The resulting fractions 52-55 were collected and were subjected to one more round of HPLC (Hypercarb, 21.2 × 100 mm, 5 μm, 1 mL injected) using a gradient of MeCN in H_2_O with 0.2% formic acid (25-55% MeCN in 15 min followed by 55-95% MeCN in 2 min followed by a hold on 95% MeCN in 7 min) yielding fractions 64-70, which were dried to yield 2.5 mg. The peptide with *m/z* 713.437/t_R_ 85.3 s was isolated similarly from cultures of UP1437 to yield 0.6 mg.

Both compounds were analysed by NMR in DMSO-*d*_6_ on a Bruker Avance-III 600 MHz NMR spectrometer at 298 K, using a 5-mm cryoprobe. 1D ^1^H and ^13^C NMR spectra were recorded along with 2D COSY, TOCSY, HSQC and HMBC experiments. Chemical shifts (Supplementary Table 3 and 4) were referenced against the residual solvent signal from DMSO-*d*_5_ (δ_C_ 39.51; δ_H_ 2.50).


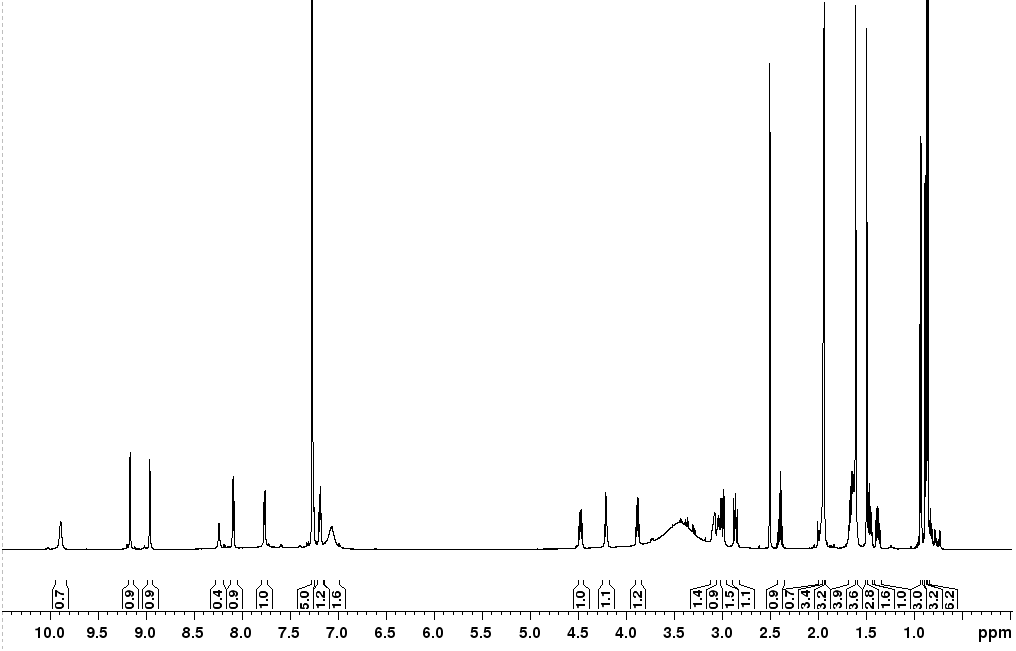


**Supplementary Figure 6.** 1D ^1^H NMR spectra for the cluster 5 peptide *m/z* 699.421/t_R_ 72.5 s, recorded at 600 MHz (298 K) in DMSO-*d*_6_.


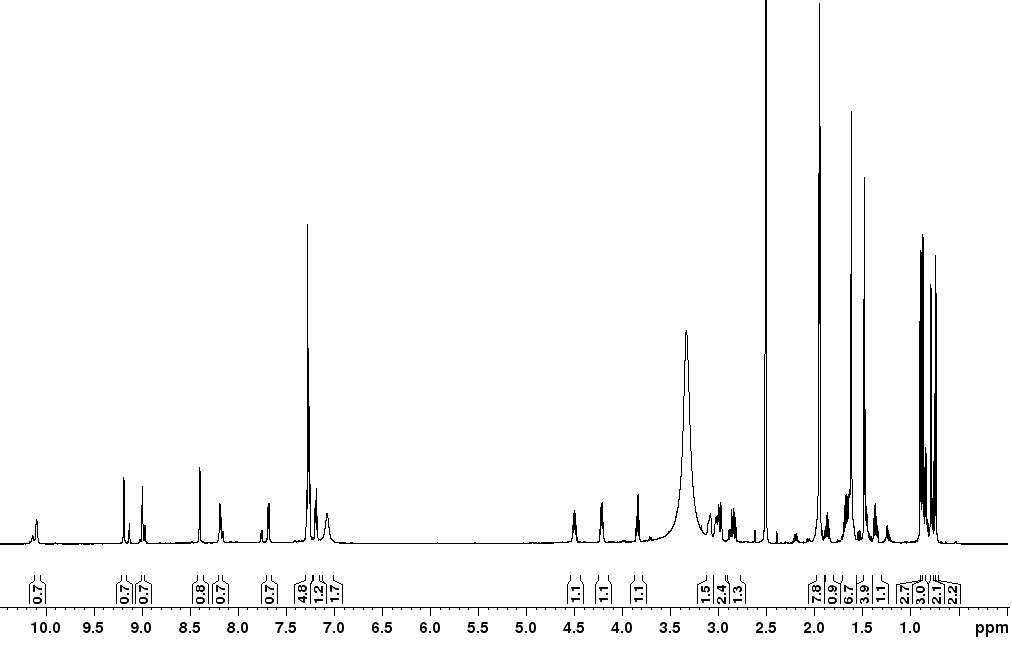


### Supplementary Figure 7. 1D ^1^H NMR spectra for the cluster 5 peptide *m/z* 713.437/t_R_ 85.3 s, recorded at 600 MHz (298 K) in DMSO-*d*_6_.

### Supplementary Table 3. NMR data for the peptide with *m/z* 699.421/t_R_ 72.5 s of cluster 5, recorded at 150/600 MHz (298 K) in DMSO-*d*_6_.

| **Res.** | **Pos.** | **δ_C_** | **δ_H_** | **mult (*J*, Hz)** | **Res.** | **Pos.** | **δ_C_** | **δ_H_** | **mult (*J*, Hz)** |
| --- | --- | --- | --- | --- | --- | --- | --- | --- | --- |
| *FA^a^* | 1 | 176.6 | - |  | *Arg* | NH | - | 7.76 | d (6.8) |
|  | 2 | 33.5 | 2.39 | sept (5.9) |  | 1 | 171.2 | - |  |
|  | 3 | 19.5 | 0.86 | d (6.8) |  | 2 | 52.9 | 4.20 | q (6.5) |
|  | 4 | 19.1 | 0.93 | d (6.8) |  | 3 | 29.1 | 1.96  1.63 | m  m |
| *Phe* | NH | - | 8.09 | d (7.5) |  | 4 | 24.4 | 1.63 | m |
|  | 1 | 170.6 | - |  |  | 5 | 40.7 | 3.08  3.04 | m  m |
|  | 2 | 54.3 | 4.47 | m |  | NH | - | 9.89 | bs |
|  | 3 | 37.0 | 2.99  2.86 | dd (13.7, 5.6)  dd (13.7, 9.3) |  | 6 | 157.3 | - |  |
|  | 4 | 137.6 | - |  |  | NH | - | 8.24 | bs |
|  | 5/9 | 128.0 | 7.27 | m |  | NH2 | - | 7.07 | bs |
|  | 6/8 | 129.2 | 7.27 | m | *DHV2* | NH | - | 8.96 | s |
|  | 7 | 126.3 | 7.18 | m |  | 1 | 164.4 | - |  |
| *DHV1^b^* | NH | - | 9.16 | s |  | 2 | 138.5 | - |  |
|  | 1 | 165.0 | - |  |  | 3 | 124.5 | - |  |
|  | 2 | 136.5 | - |  |  | 4 | 20.1 | 1.94 | s |
|  | 3 | 124.2 | - |  |  | 5 | 20.8 | 1.61 | s |
|  | 4 | 20.3 | 1.93 | s | *Leu* | NH | - | 7.26 | m |
|  | 5 | 20.9 | 1.49 | s |  | 1 | 176.0 | - |  |
|  |  |  |  |  |  | 2 | 52.7 | 3.88 | q (6.7) |
|  |  |  |  |  |  | 3 | 42.7 | 1.46  1.37 | m  m |
|  |  |  |  |  |  | 4 | 24.5 | 1.66 | m |
|  |  |  |  |  |  | 5 | 22.7 | 0.88 | d (6.8) |
|  |  |  |  |  |  | 6 | 23.0 | 0.86 | d (6.8) |

a) Fatty acid. b) Dehydrovaline (2-amino-3-methylbut-2-enoic acid)

### Supplementary Table 4. NMR data for the peptide with *m/z* 713.437 and t_R_ 85.3 s of cluster 5, recorded at 150/600 MHz (298 K) in DMSO-*d*_6_.

| **Res.** | **Pos.** | **^13^C** | **^1^H** | **mult (*J*, Hz)** | **Res.** | **Pos.** | **^13^C** | **^1^H** | **mult (*J*, Hz)** |
| --- | --- | --- | --- | --- | --- | --- | --- | --- | --- |
| *FA^a^* | 1 | 172.1 | - |  | *Arg* | NH | - | 7.68 | d (6.9) |
|  | 2 | 44.2 | 1.94 | m |  | 1 | 171.3 | - |  |
|  | 3 | 25.4 | 1.86 | sept (6.8) |  | 2 | 52.9 | 4.21 | m |
|  | 4 | 22.2 | 0.78 | d (6.5) |  | 3 | 29.2 | 1.96  1.61 | m  m |
|  | 5 | 22.1 | 0.73 | d (6.5) |  | 4 | 24.3 | 1.63 | m |
| *Phe* | NH | - | 8.18 | d (6.9) |  | 5 | 40.6 | 3.09  3.02 | m  m |
|  | 1 | 170.7 | - |  |  | NH | - | 10.10 | bs |
|  | 2 | 54.5 | 4.50 | m |  | 6 | 157.4 | - |  |
|  | 3 | 36.9 | 2.98  2.83 | dd (13.7, 5.6)  dd (13.7, 9.2) |  | NH | - | n.d. | bs |
|  | 4 | 137.6 | - |  |  | NH2 | - | 7.07 | bs |
|  | 5/9 | 128.1 | 7.26 | m | *DHV2* | NH | - | 8.99 | s |
|  | 6/8 | 129.1 | 7.26 | m |  | 1 | 164.3 | - |  |
|  | 7 | 126.3 | 7.18 | m |  | 2 | 138.6 | - |  |
| *DHV1^b^* | NH | - | 9.19 | s |  | 3 | 124.6 | - |  |
|  | 1 | 164.9 | - |  |  | 4 | 20.0 | 1.94 | s |
|  | 2 | 136.9 | - |  |  | 5 | 20.8 | 1.61 | s |
|  | 3 | 124.1 | - |  | *Leu* | NH | - | 7.26 | m |
|  | 4 | 20.3 | 1.94 | s |  | 1 | 176.1 | - |  |
|  | 5 | 20.9 | 1.47 | s |  | 2 | 52.9 | 3.83 | m |
|  |  |  |  |  |  | 3 | 43.0 | 1.45  1.36 | m  m |
|  |  |  |  |  |  | 4 | 24.6 | 1.66 | m |
|  |  |  |  |  |  | 5 | 22.8 | 0.89 | d (6.5) |
|  |  |  |  |  |  | 6 | 23.0 | 0.86 | d (6.5) |

a) Fatty acid. b) Dehydrovaline (2-amino-3-methylbut-2-enoic acid)


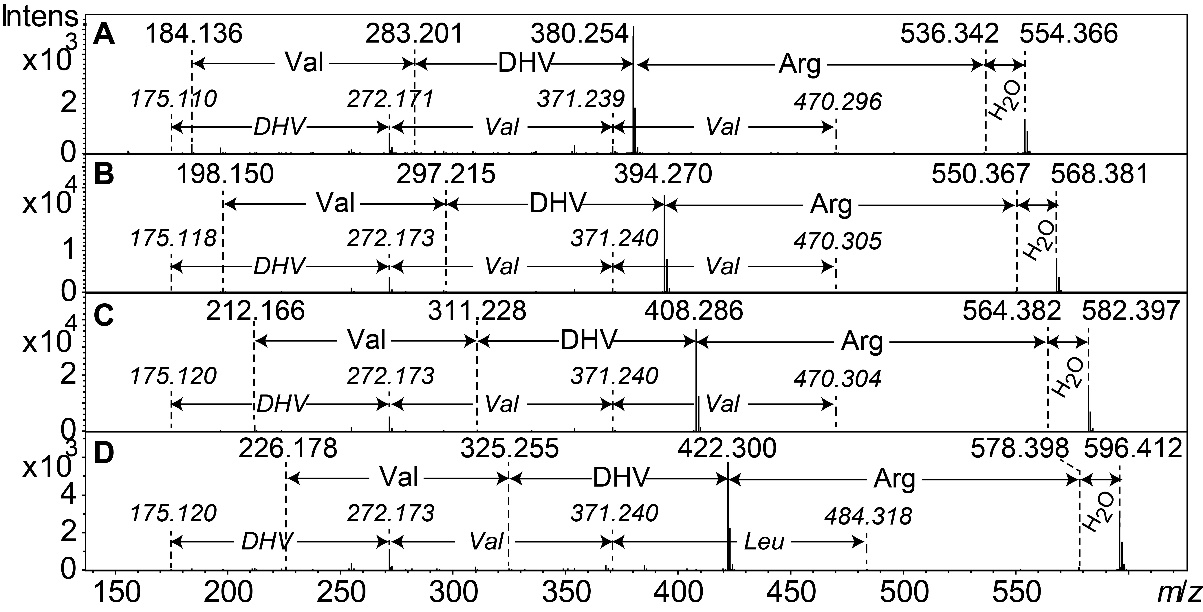


### Supplementary Figure 8. MSMS spectra of four peptides in cluster 13. Both B-type and Y-type ions are shown, with Y-type ions in italic typeface. Parent ions are shown in the top-right corner of the spectra. (DHV = dehydrovaline)


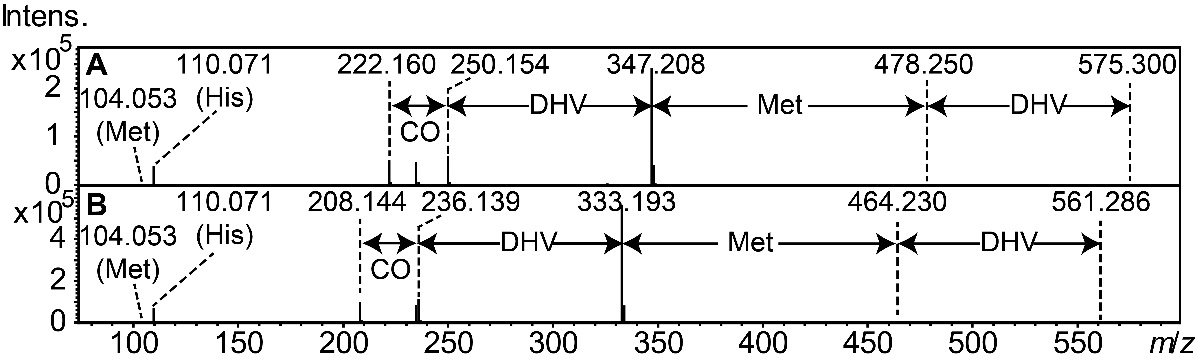


### Supplementary Figure 9. MSMS spectra of two peptides in cluster 14. B-type ions are shown together with suggested amino acid sequences. Parent ions are shown in the top-right corner of the spectra. (DHV = dehydrovaline)

1. ### Edgar R.C. 2010: Search and clustering orders of magnitude faster than BLAST. Bioinformatics 26: 2460-2461

   [↑](#footnote-ref-1)
2. Zaharia, M., Bolosky, W. J., Curtis, K., Fox, A., Patterson, D., Shenker, S., Sittler, T. (2011). Faster and more accurate sequence alignment with SNAP. arXiv:1111.5572, 1–10. [Google Scholar]. [↑](#footnote-ref-2)
3. ### Cole1, J.R., Wang Q., Fish, J.A., Chai, B., McGarrell, D.M., Sun, Y., Brown, C.T., Porras-Alfaro, A., Kuske C.R., Tiedje, J.M., 2014: Ribosomal Database Project: data and tools for high throughput rRNA analysis. Nucleic Acid Research. 42: D633-D642.

   [↑](#footnote-ref-3)
4. ### Abarenkov, K., Nilsson, R.H., Larsson, K-H., Alexander, I.J., Eberhardt, U., Erland, S., Høiland, K., Kjøller, R., Larsson, E., Pennanen, T., Sen, R., Taylor, A. F. S., Tedersoo, L., Ursing, B.M., Vrålstad, T., Liimatainen, K., Peintner U., Kõljalg, U., 2010: The UNITE database for molecular identification of fungi – recent updates and future perspectives. New Phutologist 186: 281–285Supplementary Table 1. A list of the relative abundance of the one hundred most common

   [↑](#footnote-ref-4)
